# Identification of Candidate Growth Promoting Genes in Ovarian Cancer through Integrated Copy Number and Expression Analysis

**DOI:** 10.1371/journal.pone.0009983

**Published:** 2010-04-08

**Authors:** Manasa Ramakrishna, Louise H. Williams, Samantha E. Boyle, Jennifer L. Bearfoot, Anita Sridhar, Terence P. Speed, Kylie L. Gorringe, Ian G. Campbell

**Affiliations:** 1 VBCRC Cancer Genetics Laboratory, Peter MacCallum Cancer Centre, East Melbourne, Victoria, Australia; 2 Department of Pathology, University of Melbourne, Parkville, Victoria, Australia; 3 Genetic Hearing Research, Murdoch Children's Research Institute, Royal Children's Hospital, Parkville, Victoria, Australia; 4 Bioinformatics Division, Walter and Eliza Hall Institute for Medical Research, Parkville, Victoria, Australia; Duke-NUS Graduate Medical School, Singapore

## Abstract

Ovarian cancer is a disease characterised by complex genomic rearrangements but the majority of the genes that are the target of these alterations remain unidentified. Cataloguing these target genes will provide useful insights into the disease etiology and may provide an opportunity to develop novel diagnostic and therapeutic interventions. High resolution genome wide copy number and matching expression data from 68 primary epithelial ovarian carcinomas of various histotypes was integrated to identify genes in regions of most frequent amplification with the strongest correlation with expression and copy number. Regions on chromosomes 3, 7, 8, and 20 were most frequently increased in copy number (>40% of samples). Within these regions, 703/1370 (51%) unique gene expression probesets were differentially expressed when samples with gain were compared to samples without gain. 30% of these differentially expressed probesets also showed a strong positive correlation (r≥0.6) between expression and copy number. We also identified 21 regions of high amplitude copy number gain, in which 32 known protein coding genes showed a strong positive correlation between expression and copy number. Overall, our data validates previously known ovarian cancer genes, such as *ERBB2*, and also identified novel potential drivers such as *MYNN*, *PUF60* and *TPX2*.

## Introduction

While progress has been made in elucidating the molecular events that underlie the development of ovarian cancer, the identity of the majority of genes which drive the development of this disease remain elusive. Numerous gene expression studies have identified lists of genes with significantly altered expression, but disappointingly there is little consensus between studies [1]. While gene expression studies are useful in identifying broad categories of pathways altered in cancer and clinically important subtypes [2], on their own they may not be able to distinguish the genetically altered key driver genes. An alterative strategy used to identify driver genes has been annotation of recurrent chromosomal aberrations. Early studies were hampered because the technologies for genome-wide genomic analysis lacked the resolution to adequately refine cancer associated loci [3]. The problem of resolution has been overcome with the development of ultra-high resolution aCGH and SNP arrays. Recently, our group has used these latest-generation SNP arrays to annotate even small regions (as small as 25 kb) of genomic alteration [4]. This data also demonstrated that the genetic events occurring in ovarian cancers are more numerous and complex than previously suspected. While some potential driver genes could be rapidly identified from this data due to their location on focal alterations, the majority of recurrent alterations are large and encompass numerous genes.

To expedite identification of ovarian cancer growth promoting genes we have integrated matching DNA copy number and gene expression data from a cohort of 68 primary epithelial ovarian cancers. We have particularly focused on genes in regions of copy number gain, with the expectation that expression of a driver gene within an amplicon will be more tightly correlated with gene copy number than co-amplified genes whose expression is agnostic to tumorigenesis. Integration of copy number and expression has provided a list of candidate dominantly acting driver genes, which can be used to underpin functional analysis that will be necessary to validate their contribution to ovarian tumorigenesis. In addition, the amplified and over expressed genes have the potential to serve as useful therapeutic or diagnostic markers for ovarian cancer.

## Results

### Frequency of copy number alterations (CNA) in ovarian cancer

**Figure 1:**
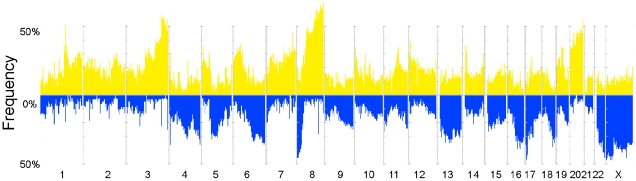
Overview of genomic aberrations in the ovarian cancer dataset (N = 72). Frequency of occurrence of genomic gains (yellow) and losses (blue) across the genome, depicted in chromosome order from 1p to Xq.

Assessment of CNA in 72 epithelial ovarian tumours (Table 1, Table S1) yielded a total of 36,534 segments comprising 20,570 CN gains and 15,964 CN losses. The median number of regions with CN gain per tumour was 208, accounting for an average of 13.6% of the genome per sample (Table S2). The median number of regions with CN loss was 194 representing 12.2% of the genome. These CNAs occurred across the genome but there were some very frequent recurrent regions of CNA among the 72 tumours (Figure 1) including gains located on 1q, 3q, 6, 7q, 8q, 19, and 20 and losses on chromosomes 4, 6, 8, 13, 16, 17, 18, 22q and X. Within epithelial ovarian cancer histotypes we noted that mucinous and to a lesser extent clear cell cases appeared to have fewer CNAs and a smaller proportion of the genome was involved compared to the other subtypes (Figure S1). However, the numbers of samples in the minor subtypes were small, making it difficult to draw statistically valid conclusions about subtype specific changes. Most of the samples were of the serous or related high grade endometrioid subtype and many of the regions of gain and loss are primarily driven by these subtypes.

**Table 1:** Summary of samples analysed by SNP and expression array.

	Grade				FIGO Stage			
Subtype	1	2	3	NK	1	2	3	NK
Clear Cell (9)	2	2	3	2	3	1	2	3
Endometrioid (14)	2	4	8	0	8	2	4	0
Mucinous (7)	5	2	0	0	4	0	2	1
Serous (37)	3	11	20	3	3	10	17	7
Undifferentiated (1)	1	0	0	0	0	1	0	0

NK, grade or stage not known. Information for 68 tumours that had both high quality expression and copy number data is listed here. Four more samples that were used in the copy number analyses alone are detailed in Table S1.

### Integration of mRNA expression in regions of frequent copy number gain

**Figure 2:**
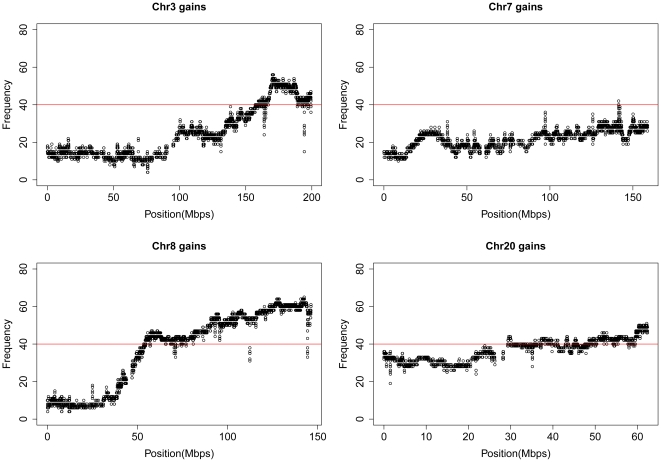
Detailed view of chromosomes showing frequent gains. Frequent gains occur on chromosomes 3, 7, 8 and 20, with each point indicating the frequency of gain of a CN segment. The red line in all panels indicates the 40% frequency threshold.

A common mechanism of activation of gene function in cancer development is through over expression as a consequence of gene amplification. While many genes may be located within a particular amplicon, the targeted gene(s) would be expected to consistently show elevated expression compared with adjacent bystander genes [5]. We have previously conducted an integrated expression analysis of candidate tumour suppressor genes within regions of loss of heterozygosity on an overlapping tumour cohort [6], thus for this study we chose to focus on the identification of candidate genes located within amplicons. An arbitrary frequency threshold of at least 40% was chosen as a filter for selecting key regions, resulting in the demarcation of multiple chromosomal regions on 3q, 7q, 8q and 20q (Figure 2). Each segment of frequent CN gain was labelled by the cytoband it belonged to; following which regions with the same cytoband tag were collapsed into one larger region (Figure S2-A). Those regions overlapping with germline copy number polymorphism (CNPs, Table S3) were excluded as described in Figure S2-B. The final 106 amplicons ranged in size from 11 kb to 7 Mb (Table S4) and 90 of these regions in total contained 1370 gene expression probesets on the Affymetrix Gene 1.0ST array corresponding to 938 known protein coding genes. The other 16 amplicons were not represented by probesets on the Gene 1.0ST arrays.

Expression analyses were carried out for probesets within each of the 90 regions (Tables 2, 3, 4, Table S5). For each region groups of samples that showed copy number gain (3 or more copies) were tested for differential expression against groups of samples that showed normal copy number (∼2 copies). Across all regions, there were 703 (51%) differentially expressed probesets corresponding to 629 genes with unique identifiers such as an HGNC gene symbol or Ensembl ID (Table S5). Only one gene, *hCG_16001*, showed a negative log fold change (−0.34, Figure S3). On average (in regions with at least 5 probesets), 50% of the probesets were found to be differentially expressed suggesting a generalised increase in expression of genes within CN gains. Interestingly, we observed that *MYC*, an oncogene characterised by copy number gain in a wide variety of tumour types, was not significantly differentially expressed between amplified and unamplified groups of samples. One possibility is that *MYC* is expressed at a high level across all tumours irrespective of the copy number status and hence is not different between groups of tumours that show a gain and those that do not. To test this possibility we compared expression of *MYC* in amplified ovarian cancer samples to expression in normal fallopian tube epithelium. We did not find any increase in *MYC* expression when comparing tumours to these samples (p = 0.41, Welch corrected unpaired t-test, Figure S4).

**Table 2:** Genes with increased expression on chromosomes 3 and 7.

Region ID	Chr	Start^1^	End	Samples “G”^2^	Samples “N”^2^	DE Probesets (%)^3^	Most significant DE Genes^4^
3_1	3	157.223	157.972	30	37	3 (60)	SSR3; TIPARP; KCNAB1
3_2	3	158.260	159.895	31	36	8 (62)	MLF1; GFM1; RSRC1; CCNL1; PTX3; VEPH1; LXN; SHOX2
3_3	3	159.895	159.959	30	37	2 (100)	RARRES1
3_4	3	159.959	161.006	32	35	2 (50)	MFSD1; SCHIP1
3_5	3	161.006	161.392	30	37	3 (75)	SCHIP1;IL12A
3_7	3	161.392	168.660	33	35	8 (24)	KPNA4; SMC4; B3GALNT1; NMD3; TRIM59; hCG_16001; IFT80
3_8	3	168.697	168.916	37	31	1 (50)	PDCD10
3_9	3	168.916	169.209	38	30	2 (100)	PDCD10; SERPINI1
3_10	3	169.209	172.478	41	27	12 (40)	MYNN; PHC3; SKIL; MDS1; ARPM1;TLOC1; PRKCI; EVI1; EIF5A2; SLC7A14
3_12	3	172.586	177.095	39	29	2 (8)	ECT2; AADACL1
3_14	3	177.366	180.518	39	29	4 (27)	TBL1XR1; PIK3CA
3_15	3	180.518	180.608	35	33	3 (100)	ZNF639; MFN1;GNB4
3_17	3	180.608	181.970	36	32	6 (43)	ACTL6A; MRPL47; NDUFB5; GNB4; LOC442098; TTC14
3_18	3	181.971	184.153	34	34	4 (57)	FXR1; DNAJC19; DCUN1D1; ATP11B
3_19	3	184.153	184.291	35	33	2 (100)	DCUN1D1;MCCC1
3_20	3	184.291	185.996	34	34	18 (50)	ABCF3*; PSMD2; AP2M1; EIF4G1; PARL; ALG3; KLHL24; POLR2H; EIF2B5*; DVL3*; YEATS2; MAGEF1; MCCC1; LAMP3; ABCC5
3_22	3	186.007	187.399	36	32	5 (31)	SENP2; TMEM41A*; SFRS10; VPS8;EHHADH
3_24	3	187.519	189.379	35	33	10 (42)	RFC4*; RPL39L*; DNAJB11; EIF4A2; TBCCD1; SNORA4; ST6GAL1; BCL6; RTP4
3_25	3	189.379	189.430	38	30	2 (100)	LPP; FLJ42393
3_27	3	193.766	193.936	35	33	1 (50)	FGF12
3_28	3	193.936	199.337	31	37	36 (46)	WDR53*; FBXO45*; NCBP2; LSG1; PIGX; RNF168; SENP5; OPA1; FYTTD1; CENTB2; UBXD7; PCYT1A; ATP13A3; KIAA0226*; DLG1
7_1	7	141.416	141.431	34	33	1 (100)	MGAM

1. Start and end position of regions in Mbp based on hg18 (March 2006 release).

2. Number of samples with copy number gains (G) and normal copy number (N) used in the expression analysis. Samples with copy number loss were not included.

3. Number of differentially expressed (DE) probesets by expression microarray. Regions with no DE named genes are not shown here but are listed in Table S4.

4. Only coding genes with a symbol (from Affymetrix array annotation) are listed here and hence can differ from the number quoted in brackets. For a full list see Table S5. Genes for each region are listed in decreasing order of significance, with only the top 15 most significant listed.

*indicates genes with a correlation coefficient of >0.7.

**Table 3:** Genes with increased expression on chromosome 8.

Region ID	Chr	Start^1^	End	Samples “G”^2^	Samples “N”^2^	DE Probesets (%)^3^	Most significant DE Genes^4^
8_1	8	53.390	55.545	29	39	11 (73)	ATP6V1H*; MRPL15; TCEA1; LYPLA1; RB1CC1; RGS20; NPBWR1; SOX17; UNQ9433
8_5	8	60.387	61.696	35	32	6 (60)	XKR4; TGS1*; TMEM68*; RP1
8_7	8	61.696	61.817	35	32	2 (100)	RAB2A; CHD7
8_13	8	62.549	65.928	33	33	3 (21)	RLBP1L1*; YTHDF3
8_15	8	66.237	68.051	33	33	14 (67)	ARMC1; VCPIP1; C8orf44; RRS1; SGK3; C8orf45; MYBL1; ADHFE1; MTFR1; C8orf46
8_16	8	68.051	68.292	29	36	6 (100)	COPS5; ARFGEF1; CSPP1
8_19	8	70.815	73.993	32	35	9 (41)	KCNB2; NCOA2; TRPA1; TRAM1; MSC
8_21	8	74.016	78.270	34	33	8 (32)	TMEM70; STAU2; PXMP3; TERF1; UBE2W; TCEB1
8_25	8	80.419	84.683	36	31	10 (40)	CHMP4C; ZNF704; ZBTB10; SNX16; ZFAND1
8_27	8	85.122	87.055	34	33	16 (67)	C8orf59; REXO1L2P; REXO1L1; E2F5
8_30	8	87.250	89.422	34	33	5 (50)	WWP1; FAM82B; CPNE3; WDR21C; CNGB3
8_31	8	89.426	93.278	37	29	7 (35)	OTUD6B; NBN; TMEM55A; SLC26A7; RUNX1T1; TMEM64
8_33	8	93.587	98.637	38	28	17 (53)	UQCRB; TP53INP1; C8orf38; MTERFD1; PLEKHF2; PTDSS1; KIAA1429; RBM35A; INTS8; TSPYL5
8_34	8	98.637	99.159	35	32	7 (88)	MTDH; LAPTM4B; MATN2; RPL30
8_36	8	99.159	100.102	37	30	9 (82)	POP1*; NPAL2; STK3; VPS13B; HRSP12; OSR2; KCNS2
8_37	8	100.112	101.579	37	30	6 (55)	COX6C; RNF19A; POLR2K; VPS13B; FBXO43
8_38	8	101.579	101.675	36	31	2 (100)	ANKRD46; MGC39715
8_39	8	101.675	105.906	39	28	25 (50)	YWHAZ; WDSOF1; FLJ45248; ATP6V1C1; ZNF706; UBR5; FZD6; PABPC1; AZIN1; MGC39715
8_43	8	107.681	110.578	39	28	4 (31)	ENY2; TTC35; NUDCD1; OXR1
8_45	8	110.578	110.700	38	29	2 (67)	EBAG9; GOLSYN
8_52	8	113.663	117.487	40	27	1 (25)	TRPS1
8_54	8	117.713	119.186	42	25	8 (89)	RAD21; C8orf53; MED30; EXT1; EIF3H; SLC30A8
8_56	8	119.298	121.983	44	23	6 (35)	MTBP;DCC1; TAF2; MRPL13; SAMD12; MAL2
8_59	8	122.661	122.935	40	27	1 (100)	HAS2
8_60	8	122.935	127.209	44	23	24 (60)	C8orf76*; RNF139; DERL1; ATAD2; TRMT12; NDUFB9; ZNF572; TMEM65; C8orf32; SQLE
8_63	8	127.320	129.639	46	21	2 (25)	FAM84B
8_66	8	129.735	131.499	47	20	4 (50)	FAM49B; MLZE; DDEF1
8_69	8	131.596	135.232	44	23	9 (45)	TG; OC90; KCNQ3; NDRG1; KIAA0143; PHF20L1; WISP1; SLA
8_72	8	135.435	136.466	43	24	2 (67)	ZFAT1*
8_76	8	137.616	139.944	42	25	2 (100)	COL22A1; FAM135B
8_78	8	140.056	146.269	43	24	89 (74)	ZC3H3*; PUF60; GPR172A; CYHR1; SCRIB; HSF1 ; ZNF7*; MAF1; SHARPIN; BOP1

1.-4. Please see legend to Table 2, except that only the top 10 genes are listed and genes present in more than one region are only shown in one of these.

**Table 4:** Genes with increased expression on chromosome 20.

Region ID	Chr	Start^1^	End	Samples “G”^2^	Samples “N”^2^	DE Probesets (%)^3^	Most significant DE Genes^4^
20_1	20	29.299	31.465	34	34	34 (62)	POFUT1; PDRG1; PLAGL2 ;ASXL1; TM9SF4; TPX2; CDK5RAP1; MAPRE1; COMMD7; KIF3B; C20orf112; RP11-49G10.8; DEFB118; DUSP15; DNMT3B
20_2	20	31.466	31.648	29	39	2 (100)	CBFA2T2; SNTA1
20_4	20	31.649	33.694	31	37	34 (74)	PIGU; DYNLRB1; GGTL3; RBM12; RALY; NCOA6*;CEP250*; APBA2BP; TRPC4AP; EIF6; EDEM2; GSS; UQCC; PXMP4; EIF2S2
20_5	20	33.696	33.760	36	32	5 (100)	RBM12; NFS1; RBM39; C20orf52
20_6	20	33.958	37.049	29	39	38 (64)	CTNNBL1*; LOC388796; KIAA0406P; DHX35*; C20orf77; ACTR5; MANBAL; FAM83D; DSN1; RBL1; C20orf198; RPN2; SCAND1; C20orf117; C20orf24
20_7	20	37.107	41.095	29	37	11 (65)	PLCG1; CHD6; LPIN3; TOP1; PTPRT; LOC149692; ZHX3; EMILIN3; MAFB
20_8	20	41.095	41.113	30	36	1 (100)	PTPRT
20_9	20	41.124	41.226	28	38	2 (100)	PTPRT
20_10	20	42.962	45.772	30	37	45 (62)	PIGT; UBE2C; ZSWIM1; TOMM34; DNTTIP1*; NCOA5; SLC35C2; ACOT8; NEURL2; KCNS1; C20orf67; SNX21; ELMO2; ZMYND8; TP53RK;
20_12	20	45.850	49.180	29	38	23 (61)	TMEM189*; MOCS3*; DPM1; STAU1; DDX27; CSE1L; ARFGEF2; ADNP; SPATA2*; PTPN1; SLC9A8*; C20orf199; PARD6B; ZNF313; KCNG1
20_14	20	49.222	54.379	30	37	10 (42)	ZFP64*; AURKA; PFDN4; ATP9A; MC3R; TSHZ2; SUMO1P1
20_15	20	54.379	54.417	27	41	2 (100)	CSTF1; AURKA
20_16	20	54.417	55.828	31	36	8 (32)	C20orf43; RAE1; BMP7; RBM38; GCNT7
20_18	20	55.991	57.887	33	34	12 (57)	VAPB; TUBB1; RAB22A; TH1L; SLMO2; STX16; ATP5E; GNAS; SYCP2; PPP4R1L; NPEPL1
20_20	20	57.901	62.427	33	34	38 (45)	LSM14B*; YTHDF1; SS18L1; DIDO1; GTPBP5; PSMA7; TAF4; C20orf11; C20orf20; TCFL5; C20orf177; MYT1; PCMTD2; DNAJC5; TPD52L2;

1.-4. Please see legend to Table 2.

To further refine this list of 703 copy number driven, differentially expressed probesets, we reasoned that those genes showing the strongest correlation of copy number and expression may be the most likely genes targeted by the CN gain. Thus, we calculated the correlation co-efficient for all differentially expressed genes with copy number probeset coverage in the candidate amplicons (Table S5). Of the 692 probesets tested (11 did not contain copy number probes), 219 (corresponding to 206 protein-coding genes) showed a strong positive correlation (r≥0.6) between expression and copy number.

### Genes targeted by high CN amplification

Our main approach to identify cancer-related genes was to filter for the most frequent aberrations but we noted that well characterised cancer driver genes, such as *CCNE1* and *ERBB2* [7], were not identified since they were amplified in less than 40% of tumours. Rather than using a lower cut-off which would risk including many regions altered due to generalised genomic instability (for example ∼67% of the genome would be considered as candidate regions if a cut-off of >10% was used), we instead filtered for genes showing a high amplitude CN gain. Here, we looked at all segments that had a copy number greater than or equal to 5 and were present in at least 5 samples, which identified 21 regions over 27.2 Mb (Table 5). These regions corresponded to 181 gene expression probesets on our Affymetrix Gene 1.0ST arrays, of which 39 (22%) had a strong positive correlation between CN and gene expression (r>0.6). These probesets corresponded to 32 known protein coding genes including well known cancer driver genes such as *ERBB2* (Table S6).

**Table 5:** Highly amplified genes.

Chr	Start (Mb)	End (Mb)	Length (bp)	No. samples	Genes^1^
3	170.040	170.248	208141	6	None
3	178.305	178.589	283690	5	TBL1XR1
3	180.121	180.410	288435	5	*PIK3CA*; ZMAT3
8	55.208	55.528	319922	5	MRPL15*
8	62.495	63.491	995369	6	RLBP1L1*;NKAIN3;ASPH
8	102.003	102.062	58823	5	YWHAZ
8	123.144	123.746	601615	6	None
8	123.856	124.369	513120	6	DERL1; ZHX2; WDR67*; ZHX1*; C8orf76*; FAM83A
8	124.369	125.825	1455953	6	ATAD2; C8orf32*; FBXO32; ANXA13; KLHL38; FAM91A1; FER1L6; MTSS1; NDUFB9; RNF139; TATDN1; TMEM65; TRMT12*
8	125.828	127.764	1936500	7	KIAA0196; NSMCE2; SQLE; ZNF572*; TRIB1; FAM84B*
8	127.764	128.973	1208920	7	*MYC*; POU5F1P1
8	128.973	130.166	1193146	8	PVT1^†^, TMEM75*
8	130.166	138.988	8821634	7	ADCY8; DDEF1; EFR3A; FAM49B; KCNQ3; MLZE; OC90; LRRC6; NDRG1; PHF20L1; SLA; TG*; TMEM71; WISP1; ST3GAL1; ZFAT*; KHDRBS3; CCDC26
8	138.988	144.000	5382420	7	FAM135B; COL22A1; KCNK9; NIBP*; CHRAC1*; EIF2C2; PTK2; DENND3*; SLC45A4; FLJ43860; GPR20; PTP4A3; ARC; BAI1; C8orf55; CYP11B1; CYP11B2; GML; JRK; LY6D; LY6K; LYNX1; LYPD2; PSCA; SLURP1; TSNARE1
17	35.104	35.105	529	5	ERBB2*
19	34.125	34.639	513414	5	UQCRFS1*
19	34.639	35.610	971542	6	C19orf12*; PLEKHF1; POP4*; *CCNE1*; C19orf2; ZNF536*
19	35.968	36.703	734619	6	TSHZ3*
19	37.459	38.011	552023	5	ANKRD27*; PDCD5*; RGS9BP; ECAT8; DPY19L3*; ZNF507*
19	38.372	39.140	767924	5	CEBPA; LRP3; SLC7A10; CHST8; KCTD15; CEBPG*; PEPD*; FLJ12355
20	29.427	29.849	421241	5	BCL2L1; COX4I2; DEFB119; DEFB121; DEFB123; DEFB124; HM13; ID1; REM1; TPX2

1. Derived from Refseq annotation (September 2009). Genes in *italics* are known oncogenes (based on Cancer Gene Census [38]), *Genes that show a strong (r>0.6) positive correlation of copy number with expression, ^†^Not on expression microarray. Note that some regions encompass multiple smaller amplicons, only genes within regions (+/−10 kb) defined by >5 samples are shown.

### Prioritising candidate driver genes

In order to prioritise the most promising candidates from the previous analyses, we built a gene list using the following criteria. Firstly, we selected those known genes with a high frequency of gain (>40%), that were differentially expressed (n = 629). From this list we selected the genes most strongly over expressed by the level of log fold change (>0.7) between samples with CN gain and samples that were neutral at the locus (n = 59). As a different measure of how gene expression was affected by copy number, we also selected genes that showed a strong correlation (>0.7) of copy number and expression (n = 58). The union of these criteria produced a list of 110 genes. From this list, we identified genes on each chromosome that were the most frequently affected by copy number change; for chr8, this included genes with a frequency of ≥60%, for chr3, ≥50% and for chr20 ≥42%. This list comprised 37 genes (Table 6).

**Table 6:** Candidate oncogenes and current literature.

Gene	Chr	Start	End	Total gain (%)	Comments	Other genes in region
PDCD10	3	168.884	168.935	43	Angiogenesis disorder [39], ERK pathway [40]	
PRKCI	3	171.423	171.506	51	Oncogene in ovarian and other cancers [41], [42]	SKIL, PHC3, MYNN
ECT2	3	173.955	174.022	50	Cytokinesis [43]. Transforming protein [44]. Interacts with PRKCI [45]	
TBL1XR1*	3	178.221	178.398	50	Oncogene in breast cancer [46], transcriptional repressor [47]	
PIK3CA*	3	180.349	180.435	50	Known oncogene	MRPL47, NDUFB5
SENP2	3	186.787	186.832	51	SUMO1 deconjugating peptidase. Possible role in degradation of beta-catenin [48].	TMEM41A
MRPL15*	8	55.210	55.224	42	Mitochondrial ribosomal protein [49]	
RLBP1L1*	8	62.363	62.577	46	Clavesin 1 (CLVS1), regulates endosome morphology [50], upregulated in liver cancer [51]	
YWHAZ*	8	102.000	102.035	53	14-3-3 isoform zeta, oncogenic functions in inhibiting apoptosis and adhesion [52]	
DERL1*	8	124.095	124.124	60	Endoplasmic reticulum protein [53] with role in stress response. Elevated expression in cancer [54], [55]	WDR67*, C8orf76*
ATAD2*	8	124.401	124.478	60	ATPase. E2F target, binds MYC, expression correlates with poor outcome in breast cancer [56]. Interacts with ER and AR and is required for target gene expression [57]	WDYHV1/C8ORF32*, FBXO32*, FAM91A1*
RNF139*	8	125.556	125.570	60	Translocation causes hereditary renal cancer. Interacts with VHL [58]	NDUFB9*, TRMT12*, TMEM65*, SQLE*
FAM84B*	8	127.634	127.640	61	–	
FAM49B*	8	130.923	131.021	61	–	
NDRG1*	8	134.319	134.379	60	Diverse role in stress response including hypoxia [59]. Fusions with ERG in prostate cancer [60].	
ZFAT*	8	135.559	135.794	60	Zinc finger and AT hook protein, anti-apoptotic role [61]	
PTK2*	8	141.738	142.081	60	Focal adhesion kinase. Involved in signal transduction for proliferation[62]	CHRAC1*, NIBP/TRAPPC9*, SLC45A4*
PTP4A3*	8	142.501	142.511	60	Protein tyrosine phosphatase. Increases proliferation and metastasis [63]	JRK*, TSTA3, ZC3H3, LY6E
PUF60	8	144.971	144.984	60	mRNA splicing factor [25]	CYC1, ZNF623, ZNF7, CYHR1
ERBB2*	17	35.098	35.138		Known oncogene in breast cancer	
TPX2*	20	29.791	29.853	42	Activator of Aurora-A and involved in spindle assembly [30]. Interacts with BRCA1/BARD1 [64]	
UBE2C	20	43.875	43.879	42	Ubiquitin-conjugating enzyme E2C, degradation of mitotic cyclins and cell cycle progression [65]	PIGT
ZFP64	20	50.134	50.242	43	Zinc finger protein, Notch signalling [66]	
AURKA	20	54.378	54.401	43	Aurora kinase, cell cycle regulation, chromosome segregation, microtubule/spindle function [67]	CSTF1, RAE1, C20orf43
SS18L1	20	60.152	60.191	46	Synovial sarcoma translocation fusion gene [68]); calcium-responsive transactivator [67]	GTPBP5, LSM14B, TAF4

Genes were selected as follows: Gain in >40% and differentially expressed, with fold change expression in gain vs. neutral of >0.7 or correlation coefficient (r) of >0.7. Of these genes (n = 121), the most frequently gained in each chromosome were selected: Chr 3 n≥50, Chr8 n≥60, Chr 20 n≥42. 2. High level amplification in at least 5 samples (*), and differentially expressed, with fold change expression in gain vs. neutral of >0.6 or correlation coefficient (r) of >0.6. Chr19 genes (n = 12) are not shown here.

Secondly, we also wished to include genes that were highly amplified. From our list of highly amplified genes in at least 5 samples we selected those that had a strong positive correlation between copy number and expression (r>0.6, n = 32). Some of the genes that were highly amplified were also differentially expressed based on the expression analysis of frequently gained regions, so we also included genes with a log fold change greater than 0.6 (n = 17). Taking genes satisfying one or the other of these criteria, we added 41 genes to our high priority list (Table 6).

When we combined these two gene lists, the first based on “high frequency” and the second on “high amplitude” but both with increased expression, the final number of unique genes was 70 (Table 6).

## Discussion

Gene expression analysis has been widely used to identify key pathways and clinically important subgroups in ovarian cancer but identification of specific driver genes using this methodology alone has been hampered by the fact that expression is rather plastic and there has been little consensus in the genes identified between such studies [1], [8]. One reason for this lack of consistency is that most studies have analysed RNA from whole tumour samples without verification of the percentage cancer epithelium and/or have used diverse control tissues such as whole ground ovary [9]. In contrast to gene expression, genomic alterations may be a more stable and reliable predictor of the location of driver genes. Ovarian cancer has long been suspected to be cytogenetically complex [10] and recent advances in genomics technology has confirmed the profound genomic aberrations that characterise most ovarian cancers [4], [11], [12], [13]. Despite this complexity, published copy number profiles of ovarian cancers are highly comparable at a global level [3] and many studies have identified very similar regions of frequent copy number alteration. However, progress at identifying key driver genes has been slow, with different studies often identifying different candidates in the same genomic region. For example, the chromosome 20 amplicon driver has variously been suggested to be *ADRM1* [14], *EYA2* [15], *AURKA* and *ZNF217* [16], among several others. Early studies integrating expression and copy number data have either used cancer cell lines to identify over expressed genes [17], [18] and/or microarray platforms with limited resolution and genome coverage [19], [20]. To date few studies have exploited a truly genome-wide integrated copy number and expression analysis on matched samples for the unbiased identification of candidate genes [21], [22], [23] and there has only been one previous study of a smaller cohort of ovarian tumours [12]. In this study we have therefore attempted to circumvent some of the issues of examining expression or copy number in isolation by integrating two data sets obtained from microdissected tumour epithelial cells.

As a first pass of the data we focussed on gains occurring in a very high proportion of cases which included regions of chromosomes 3, 7, 8 and 20. Identification of differentially expressed genes reduced our list of candidate cancer genes in these regions by approximately half (range 6–89% for regions with at least 5 probesets). We have validated several of the genes identified in Haverty *et al*., for example, on 3q26.2 we confirmed increased expression in 7/8 of their genes. However, we have also identified a number of additional amplified and over expressed genes (Tables 2, 3, 4), most likely due to differences in our method and larger sample size. The proportion of differentially expressed genes in our study is consistent with previous studies of other cancer types [24] supporting the concept that copy number can have a strong influence on gene expression. Consequently, for many regions we were not able to identify one particular driver gene. It is possible that there may truly be many driver genes within each amplicon and although each may individually contribute little to cancer progression, coordinate over expression of these genes in amplified regions may have an additive or synergistic oncogenic effect. Alternatively, many of the differentially expressed genes may be passengers whose over expression endows no selective advantage or disadvantage to the tumour. Discriminating between passengers and drivers within a genomic region may therefore only be achieved through large-scale functional analyses and combinatorial approaches examining many genes in concert.

Despite the relatively large number of amplified and differentially expressed genes identified in this study, we still hypothesise that those genes showing the strongest over expression, and also those genes with the highest amplitude copy number gains, may be more likely to be drivers of tumorigenesis than weakly over expressed genes. Hence, we prioritised our gene list using stringent expression criteria. For example, one of the genes most frequently targeted by copy number that is strongly over expressed is *PUF60* (*poly-U binding splicing factor 60 kDa*). This gene encodes for a pre-mRNA splicing factor thought to be involved in the recognition of 3′ splice sites [25]. It may also inhibit transcription by interacting with the TFIIH helicase, the key factor mutated in the cancer-prone syndrome xeroderma pigmentosum, and this interaction is implicated in the correct regulation of *MYC* transcription [26], [27].

**Figure 3:**
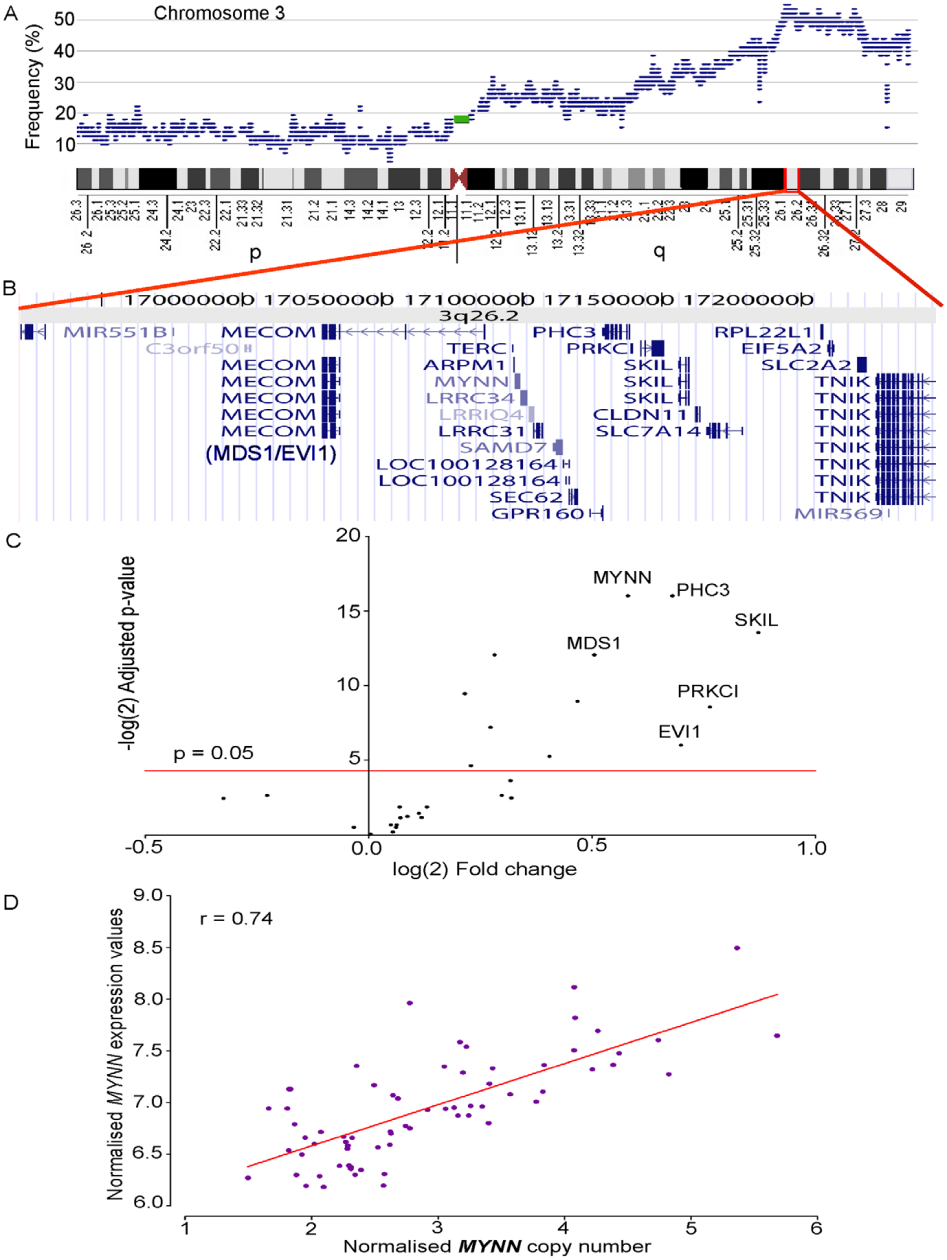
Correlation between copy number and expression for a frequently gained region on cytoband 3q26.2. A. Frequency of copy number gain on chromosome 3 from p-ter at left to q-ter at right as indicated by the ideogram. B. Genes on Chr3: 169.209–172.478 Mbp, a region gained in 60% (41/68) of all samples, including genes previously associated with ovarian cancer (*PRKCI, MECOM* or *MDS1/EVI1*) and potentially novel oncogenes (*MYNN*). C. A volcano plot presenting the results of expression analyses between amplified and unamplified samples in this region. The genes in the top right corner are significantly overexpressed in samples with copy number gain (p<0.05; above the red line at –logP 4.32) compared to samples without copy number change (selected genes are labelled). For full list of differentially expressed genes see Table S5. D. Plot comparing copy number and expression in all samples for the gene *MYNN* that showed the highest correlation (r = 0.74, Pearson's test) between copy number and expression for this region on 3q26.2.

Myoneurin or *MYNN* is a gene that is located in a region of frequent (60%) copy number gain on 3q26.2. It is differentially expressed (adjusted p = 1.51E-05) between amplified and unamplified groups, and shows the strongest correlation between copy number and expression (r = 0.74, Figure 3) amongst all genes in this region. This gene was identified as a member of the Broad complex, Tramtrack, Bric a' brac (BTB) or poxvirus and zinc finger (POZ)-ZF i.e BTB/POZ-ZF family of transcription factors [28]. First discovered in *Drosophila*, this family consists of about 60 human proteins including several cancer related proteins such as leukaemia related factor (LRF/ZBTB7) and B-cell lymphoma 6 (BCL6). While the role of MYNN in cancer is yet to be characterised, other members of this family are similarly overexpressed in tumors [29].

As well as identifying high frequency, differentially expressed genes, including known cancer genes such as *PIK3CA* and *AURKA*, we also used high amplitude regions to locate additional known (e.g. *ERBB2* and *CCNE1*) and potential oncogenes. For example, on chromosome 20, the high-amplitude approach identified a small minimal region that was not evident from the low-amplitude analysis. This 421 kb interval at 20q11.21 encompasses 10 genes, of which *TPX2* showed the strongest correlation with copy number (r = 0.53). This gene was also differentially expressed between samples with any *TPX2* gain and those with normal *TPX2* copy number, and had the strongest fold change of any gene on chromosome 20 (log2 fold change of 1.03). The protein encoded by this gene functions as an activator of Aurora-A with a role in spindle assembly [30]. Interestingly for ovarian cancer, it has been shown to interact with the BRCA1/BARD1 complex (15). Recently, it has been identified as a potential oncogene in pancreatic cancer [31].

In summary, our study shows that combining the high frequency and high amplitude analyses and targeting the most strongly over expressed genes reduced the candidate list to just 70 genes out of the many thousands targeted by copy number change alone. We have identified many promising candidate genes not previously noted in ovarian cancer, particularly genes such as *MYNN*, *TPX2* and *PUF60*. It should be noted, however, that our method of analysis is one of many that can be employed in the identification of novel cancer genes, and is unlikely to have identified all possible candidates. The example of *MYC*, not strongly expressed in our data but previously shown to have a functional effect in ovarian cancer cell lines [32], clearly indicates that our approach should be considered complementary to others such as functional screens and deep sequencing of primary cancer samples. Nevertheless our data provides an important platform from which to rationally pursue the validation of these potential dominant drivers of ovarian tumorigenesis. In addition, this list may include genes that are valid candidates for diagnostic or therapeutic purposes.

## Materials and Methods

### Ethics Statement

All samples were collected with the donor's written informed consent. This study was approved by the Peter MacCallum Cancer Centre Human Research Ethics Committee (Protocol number 01/38).

### Sample collection

Tumour biopsies were obtained from 72 patients who were undergoing surgery for primary ovarian cancers (a) at hospitals in the Wessex region of Southeast England, UK and (b) in hospitals in Victoria, Australia (accessed through the Peter MacCallum Cancer Centre Tissue Bank). Blood was collected from the same patients for matching lymphocytes. Fallopian tube samples were collected through the tissue bank from *BRCA1* or *BRCA2* mutation carriers undergoing prophylactic bilateral salpingo-oophorectomy in hospitals around Melbourne. The accrual and use of patient samples related to this project were approved by the relevant institutional ethics committees. Clinical and histopathological information about the samples are provided in Table 1 and Table S1.

### DNA and RNA extraction

Fresh-frozen tissue was embedded in Optimal Cutting Temperature Compound (OCT, Sakura Finetek, Torrance, CA) and cut into 10 µm sections. Tumour DNA and tumour and fallopian tube RNA were extracted from identical regions after needle micro-dissection of >80% tumour epithelial cells. Sections for RNA were stained using Cresyl violet and RNA was extracted using Ambion mirVana total RNA extraction protocol (Applied Biosystems/Ambion, Austin, TX). Tissue sections used for DNA extraction were stained with haematoxylin and eosin and DNA was extracted using the Qiagen Blood and Tissue Kit (Qiagen, Valencia, CA, USA). DNA from matching normal lymphocytes for samples from the Peter MacCallum Cancer Centre Tissue Bank were extracted using the same kit. DNA from matching normal lymphocytes for samples from Southampton were extracted as described previously [33].

### Microarray data generation and quality control

500 ng of DNA from each tumour sample was analysed using the Affymetrix Genome-wide Human SNP Array 6.0 (SNP6.0) following the manufacturer's instructions (Affymetrix, Santa Clara, CA). Where available (57 cases) DNA from matching peripheral blood lymphocytes was analysed on the same platform and in the same batch. For mRNA expression, 300 ng of total RNA from the same tumour samples were analysed using the Affymetrix Human Gene1.0 ST Array. Analysis of array performance for SNP6.0 arrays was performed using genotyping call rates (>90% call rate required) and also visual inspection of copy number traces to remove noisy samples. 72 samples passed quality control measures and were used in the copy number analysis. For expression arrays, the profiles of hybridisation controls, spike-in controls and positive-versus-negative area under the curve (AUC) were assessed using Affymetrix Expression Console. Additionally, the quality of the arrays was assessed based on Relative Log-Likelihood (RLE) and Normalised Unscaled Standard Errors (NUSE) criteria generated using the “affyPLM” package in the R open-source software. Expression arrays that were flagged as dubious by 2 out of 3 measures (AUC, RLE, NUSE) were excluded from expression analyses. 68 tumour samples (57 with normal DNA) passed for both expression and copy number and were retained in the integrated expression analyses. The final sample set in the integrated analysis included the four most commonly seen histological subtypes of ovarian cancer – serous (n = 37), endometrioid (n = 14), mucinous (n = 7) and clear cell (n = 9). One sample in the study was of unknown histotype (Table 1). Both gene expression and copy number data are MIAME compliant and have been submitted to the National Centre for Biotechnology Information's (NCBI) Gene Expression Omnibus (GEO) website, series accession number GSE19539.

### Copy number analysis

Copy number generation and analyses were performed using Partek^®^ Genomics Suite™ version 6.03 (Partek Inc., St. Louis, Missouri) and Bioconductor packages in the R-open source software framework [34], [35]. SNP 6.0 CEL files were imported into Partek using default settings for background correction and summarisation. Human Genome Build 36.1 (hg18, March 2006) was used for base pair locations. Probeset copy number ratios were calculated by comparing each tumour with its matching normal when available (n = 57). For samples that did not have matching normal data (n = 15), a pooled normal baseline from all the other normal samples was used. Circular binary segmentation [36] was performed using the R-based package “DNAcopy” to segment the data into distinct regions of change using default package settings. This analysis produced a list of regions per sample that was then filtered for those regions that showed gain (copy number ratio >2.5) or loss (copy number ratio <1.5) across ≥40% (n≥29) of all samples. These regions were collapsed into cytobands for easier data manipulation (Figure S2 for more detail). It is important to note that since these regions have undergone filtering steps defined above, they do not include the entire cytoband by which they are represented and hence the high resolution of the data is not compromised.

To identify potential germline copy number polymorphisms (CNP) that could interfere with accurate identification of somatic changes, copy number data for 57 normal samples was generated relative to a pooled baseline of all normal samples. Regions showing gain or loss in >5% of all samples were called as CNPs (Table S3). Regions of interest from the tumour data were scanned for these CNPs and matches were removed from downstream analyses (Figure S2-B). CNP-removed, cytoband-collapsed regions were queried against the entire copy-number dataset to generate accurate, region-wise values of copy number.

Copy number was extracted on a gene-by-gene basis to perform Pearson correlation analysis with expression. Since some genes were so small that there were no copy number probesets mapping to them, an additional 10 kb was added to all gene start and stop positions before extracting their copy number.

### Expression microarray analysis

For each candidate region, samples were divided into two groups, G – consisting of all samples that showed gain (>3 copies) on the SNP6.0 platform; and N – consisting of all samples that showed normal copy number (1.5–2.5 copies). A test for differential expression was performed between these two groups using the “limma” package available on the R-open source software platform [34]. Histological subtype was included as a factor in the analysis. Genes were considered to be significantly differentially expressed with a p-value of <0.05 after multiple testing correction [37]. A Pearson's correlation analysis between copy number and expression was also performed. Separate analyses were performed on a gene-by-gene basis for all genes within (a) most frequently amplified regions (CN≥3; Freq≥40%) and (b) most highly amplified regions (CN≥5; Freq≥7%).

## Supporting Information

Table S1 Sample details. Clinicopathological features and assay information for each sample. 57 out of 72 tumours had matching lymphocytic DNA available for copy number microarray analysis.
(0.06 MB PDF)

Table S2 Proportion of genome-wide gain and loss by sample. In all of these samples, the aberrant genome adds up to 95.4% on average. The missing 4.6% can be attributed to regions on chromosome Y, Mitochondrial DNA and repetitive sequences around centromeric regions that are either removed from the segmentation analysis or not covered by the Affymetrix SNP6.0 array.
(0.06 MB PDF)

Table S3 Germline copy number polymorphisms on Chr 3, 7, 8, 20. The regions/segments of copy number gain that contained one or more of these CNPs were removed or altered as displayed in Figure S1-B. The type of CNP is also displayed in the far right column.
(0.05 MB PDF)

Table S4 Regions of gain present in >40% of samples. This table contains genomic information for the 90 regions included in the expression analyses, i.e., all those regions that mapped to 1 or more probesets on the Human GeneST1.0 microarrays. On this microarray platform, most probesets map uniquely to a protein-coding gene. The region IDs correspond to those in Tables 2, 3, 4 and S5.
(0.13 MB PDF)

Table S5 All differentially expressed probesets in frequent regions of gain. Every probeset tested for differential expression is listed and tagged by the region it belongs to. These region IDs are consistent across all tables in the paper and are derived as shown in Figure S1-A. Column 5 displays the Pearson's correlation between copy number and expression for the listed probeset. Columns 6–11 are derived from differential expression analyses performed using the “limma” package in R.
(0.06 MB PDF)

Table S6 Correlation for all genes highly amplified (CN>5) in at least 5 samples. This table displays Pearson's correlation between copy number and gene expression for all 181 probesets in regions of high CN gain across the genome. The p-value displayed is a raw p-value obtained while testing for correlation. * Genes highly amplified in 4 samples but that were within 10 kb of a copy number breakpoint of 5 amplified samples.
(0.03 MB PDF)

Figure S1 Subtype breakdown of genome wide CN changes. (A) Overall copy number landscape for the cohort of ovarian cancer samples. This is similar to Figure 1 with the exception that the y-axis ranges from 0–100% of samples as opposed to 0–50%. Below are the distribution of copy number changes for (B) 37 serous ovarian cancers, (C) 14 endometrioid ovarian cancers, (D) 7 mucinous ovarian cancers and (E) 9 clear cell ovarian cancers. A, B and C jointly show that the major contributors for the high frequency changes are serous and endometrioid tumours. Data for the single tumor classified as undifferentiated is not shown here.
(0.43 MB TIF)

Figure S2 ‘Cytoband collapsing’ and the exclusion of CNPs. (A) Shows the steps taken towards obtaining the copy number regions. The starting data (far left) contains genomic position and copy number information for segmental overlaps. All segments at this step of analysis occur with >40% frequency and have 3 or more copies. Letters a, b, r, s, t, u, v and w refer to genomic start/stop sites in basepairs. Regions are sorted by chromosome, then by genomic start and finally by genomic stop positions. Following this they are annotated with their cytobands and the newly defined “collapsed” region is bounded by the lowest start (a) and highest stop (b) positions and annotated with the cytoband of origin. The ‘a’ and ‘b’ from here carry through to part B of the figure. Regions that span two cytobands are listed as a separate group as shown in Table S4. (B) Shows the rules used to eliminate CNPs from the cytoband regions. Regions such as “Amp 4” are split into two, resulting in more regions after CNP elimination than before. (C) Regions of CNP across the genome and their position in relation to regions of copy number gain relevant to our study. (i) Global changes in normal (n = 57, green  =  gain and red  =  loss) and tumour (n = 72, yellow  =  gain and blue  =  loss) samples. We define a CNP as a change that occurs in at least 5% of normal samples. CNPs often show both genomic gain and loss at the same locus in normal samples. (ii) All changes on Chromosome 3 and in particular a CNP on 3q26.1 between 168.66 and 168.69 Mbp highlighted by the black oval, observed in >15% of all normal samples. (iii) The 3q26.1 CNP occurs in the middle of a region of copy number gain that we investigate further. This CNP region was removed from the data in accordance with S2-B.
(0.55 MB PDF)

Figure S3 Expression of all genes in regions of frequent copy number gain. This figure displays all genes in 90 regions of copy number change in terms of their average expression and t-statistic, resulting from the test for differential expression for each of these regions between amplified and unamplified samples. Genes showing a significant differential expression are represented by red dots and non-significant genes are represented by purple dots. Only one gene hCG_16001 showed a significant reduction in expression under the influence of copy number gain. This is a ribosomal protein L23a pseudogene 42 (RPL23A42) where RPL23A encodes a ribosomal protein that is a component of the 60S subunit and may be one of the target molecules involved in mediating growth inhibition by interferon.
(0.09 MB TIF)

Figure S4 Expression of MYC across various sample groups. RMA normalised expression of MYC based on Gene 1.0 ST array data. No significant differences were found between groups of samples that showed copy number gain in the region and those that did not.
(0.15 MB TIF)
