# Correction: Identification of Candidate Growth Promoting Genes in Ovarian Cancer through Integrated Copy Number and Expression Analysis

**DOI:** 10.1371/annotation/4056b510-e92d-4472-871f-2cf1f6834689

**Published:** 2011-07-19

**Authors:** Manasa Ramakrishna, Louise H. Williams, Samantha E. Boyle, Jennifer L. Bearfoot, Anita Sridhar, Terence P. Speed, Kylie L. Gorringe, Ian G. Campbell

The authors would like to acknowledge that Figure 1 was generated using the Progenetix website (http://www.progenetix.org) and the paper should have cited the following papers:

Baudis, M. 2006. Online database and bioinformatics toolbox to support data mining in cancer cytogenetics. Biotechniques 40, no. 3: 296-272.

Baudis, M, and ML Cleary. 2001. Progenetix.net: an online repository for molecular cytogenetic aberration data. Bioinformatics 12, no. 17: 1228-1229. 

