# Supplementary material for: Identification of Candidate Growth Promoting Genes in Ovarian Cancer through Integrated Copy Number and Expression Analysis
Source: PLoS One. 2010 Apr 8;5(4):e9983. doi: 10.1371/journal.pone.0009983 (PMC2851616; doi:10.1371/journal.pone.0009983)
Supplement: Table S1 — Sample details. Clinicopathological features and assay information for each sample. 57 out of 72 tumours had matching lymphocytic DNA available for copy number microarray analysis. (0.06 MB PDF) [file pone.0009983.s001.pdf]

Sample Name	Subtype	Grade	Stage	Age	Paired?	SNP array	Expr array
IC022	serous	3	3	65	Y	Y	Y
IC026	serous	2	3	51	Y	Y	Y
IC095	endometrioid	3	2	66	Y	Y	Y
IC121	mucinous	1	3	77	Y	Y	Y
IC128	endometrioid	2	1	56	Y	Y	Y
IC138	mucinous	1	1	47	Y	Y	Y
IC151	endometrioid	2	2	56	Y	Y	N
IC179	endometrioid	3	3	62	Y	Y	Y
IC201	serous	2	3	80	Y	Y	Y
IC220	endometrioid	2	3	62	Y	Y	Y
IC257	mucinous	1	1	60	Y	Y	Y
IC258	endometrioid	3	3	69	Y	Y	Y
IC288	serous	2	3	51	Y	Y	Y
IC293	endometrioid	3	1	59	Y	Y	Y
IC300	endometrioid	3	3	68	Y	Y	Y
IC315	serous	3	-	82	Y	Y	Y
IC318	serous	3	3	70	Y	Y	Y
IC321	mucinous	2	-	47	Y	Y	Y
IC325	serous	3	-	64	Y	Y	Y
IC328	serous	2	-	66	Y	Y	Y
IC349	clear cell	2	-	39	Y	Y	Y
IC382	serous	3	3	59	Y	Y	Y
IC407	serous	2	3	83	Y	Y	Y
IC413	serous	3	3	36	Y	Y	Y
IC419	clear cell	1	1	65	Y	Y	Y
IC434	endometrioid	3	1	76	Y	Y	Y
IC448	mucinous	1	1	55	Y	Y	Y
IC487	serous	1	3	41	Y	Y	Y
IC493	serous	2	3	61	Y	Y	Y
IC499	serous	-	2	49	Y	Y	Y
IC504	endometrioid	3	1	50	Y	Y	Y
IC511	clear cell	1	3	61	Y	Y	Y
IC548	serous	1	3	61	Y	Y	Y
IC549	clear cell	3	-	42	Y	Y	Y
IC557	mucinous	1	1	35	N	Y	Y
IC579	serous	3	-	71	N	Y	Y

IC580	endometrioid	2	1	70	Y	Y	Y
IC594	endometrioid	3	1	47	Y	Y	Y
P0706	serous	2	-	57	Y	Y	Y
P0933	serous	3	2	85	Y	Y	Y
P0985	unknown	3	2	74	N	Y	Y
P1049	serous	3	2	50	Y	Y	Y
P1094	clear cell	-	1	55	Y	Y	Y
P1246	serous	2	2	69	Y	Y	Y
P1348	serous	3	2	42	Y	Y	Y
P1389	serous	-	2	67	Y	Y	N
P1428	serous	2	2	81	Y	Y	Y
P1436	serous	3	3	61	Y	Y	Y
P1555	serous	3	2	52	Y	Y	Y
P1556	clear cell	3	-	39	N	Y	Y
P1680	clear cell	3	2	71	Y	Y	Y
P1768	endometrioid	2	1	51	Y	Y	Y
P1854	serous	2	3	76	N	Y	N
P1921	endometrioid	1	1	63	Y	Y	Y
P1953	serous	3	3	51	N	Y	Y
P1977	endometrioid	1	2	72	N	Y	Y
P2125	serous	3	3	56	Y	Y	Y
P2205	serous	-	1	50	N	Y	Y
P2506	clear cell	-	1	73	Y	Y	Y
P2712	serous	3	1	56	N	Y	Y
P2803	serous	2	2	46	Y	Y	Y
P2808	mucinous	2	3	55	Y	Y	Y
P4075	serous	-	-	54	N	Y	Y
P4076	serous	-	-	56	N	Y	N
P4085	serous	1	2	77	N	Y	Y
P4093	serous	3	-	60	N	Y	Y
P4178	serous	3	3	64	Y	Y	Y
P4219	serous	3	3	44	Y	Y	Y
P4616	serous	3	1	53	Y	Y	Y
P4684	serous	3	3	73	Y	Y	Y
P5390	clear cell	2	3	61	N	Y	Y
P7820	serous	2	2	54	N	Y	Y
