# Supplementary material for: Identification of Candidate Growth Promoting Genes in Ovarian Cancer through Integrated Copy Number and Expression Analysis
Source: PLoS One. 2010 Apr 8;5(4):e9983. doi: 10.1371/journal.pone.0009983 (PMC2851616; doi:10.1371/journal.pone.0009983)
Supplement: Table S2 — Proportion of genome-wide gain and loss by sample. In all of these samples, the aberrant genome adds up to 95.4% on average. The missing 4.6% can be attributed to regions on chromosome Y, Mitochondrial DNA and repetitive sequences around centromeric regions that are either removed from the segmentation analysis or not covered by the Affymetrix SNP6.0 array. (0.06 MB PDF) [file pone.0009983.s002.pdf]

Sample	Subtype	%Genome Gained (a)	%Genome Lost (b)	%Genome Neutral (c)	Total Genome coverage (a+b+c)
IC022	serous	20.2	8.5	66.7	95.4
IC026	serous	25.6	21.0	48.3	95.5
IC095	endometrioid	19.3	6.8	69.3	95.5
IC121	mucinous	0.0	1.8	93.7	95.5
IC128	endometrioid	1.3	0.0	94.2	95.5
IC138	mucinous	6.0	12.3	77.1	94.9
IC151	endometrioid	24.1	13.4	57.9	95.5
IC179	endometrioid	18.3	20.5	56.6	95.5
IC201	serous	27.8	18.9	48.4	95.5
IC220	endometrioid	14.9	15.7	64.9	95.2
IC257	mucinous	0.5	0.7	94.3	95.5
IC258	endometrioid	15.9	8.8	70.8	95.4
IC288	serous	13.3	21.0	61.1	95.3
IC293	endometrioid	20.7	16.6	57.9	95.5
IC300	endometrioid	13.6	0.6	81.2	95.4
IC315	serous	20.7	7.2	67.5	95.3
IC318	serous	18.1	11.3	66.0	94.4
IC321	mucinous	23.7	8.7	63.0	95.5
IC325	serous	1.8	3.4	90.2	95.5
IC328	serous	13.7	22.8	58.9	95.2
IC349	clear cell	4.0	4.5	87.0	95.4
IC382	serous	21.3	19.8	54.1	95.5
IC407	serous	26.2	27.9	41.1	95.4
IC413	serous	0.0	0.0	95.5	95.5
IC419	clear cell	0.9	2.9	91.8	94.9
IC434	endometrioid	22.8	12.4	59.7	95.4
IC448	mucinous	0.0	0.1	95.3	95.3
IC487	serous	0.0	0.0	95.5	95.5
IC493	serous	15.7	11.6	68.0	94.5
IC499	serous	4.4	20.4	70.6	95.4
IC504	endometrioid	23.6	14.9	56.7	95.4
IC511	clear cell	8.6	0.1	86.8	95.4
IC548	serous	13.6	0.0	81.8	95.2
IC549	clear cell	0.0	0.0	95.5	95.5
IC557	mucinous	0.1	0.2	95.0	95.4

IC579	serous	13.4	10.5	71.4	95.5
IC580	endometrioid	13.5	10.3	70.6	95.4
IC594	endometrioid	44.0	2.1	49.5	95.5
P0706	serous	13.8	14.5	66.8	95.2
P0933	serous	14.5	4.0	77.0	95.4
P0985	unknown	10.2	6.3	78.8	95.4
P1049	serous	12.1	15.8	67.6	95.4
P1094	clear cell	16.9	4.2	74.4	95.5
P1246	serous	19.6	20.3	54.5	95.2
P1348	serous	13.5	19.4	62.5	95.5
P1389	serous	19.5	13.8	61.9	95.2
P1428	serous	2.4	3.9	89.2	95.1
P1436	serous	21.9	18.1	55.2	95.4
P1555	serous	8.8	16.2	70.4	95.4
P1556	clear cell	12.3	10.0	73.1	95.4
P1680	clear cell	21.0	30.1	44.0	95.4
P1768	endometrioid	0.0	1.9	93.6	95.2
P1854	serous	23.6	12.1	59.5	95.4
P1921	endometrioid	24.1	19.2	51.9	95.2
P1953	serous	20.0	19.0	56.4	95.3
P1977	endometrioid	14.2	1.1	80.1	95.5
P2125	serous	14.3	12.6	68.5	95.4
P2205	serous	13.3	27.2	54.9	95.3
P2506	clear cell	11.0	5.1	79.4	95.5
P2712	serous	24.4	7.1	63.9	95.5
P2803	serous	9.7	28.1	57.6	95.2
P2808	mucinous	19.0	16.8	59.4	95.3
P4075	serous	17.7	16.6	61.1	95.4
P4076	serous	3.9	26.0	65.6	95.4
P4085	serous	0.6	5.1	89.6	95.4
P4093	serous	12.8	20.1	62.3	95.2
P4178	serous	12.8	19.0	63.6	95.5
P4219	serous	22.1	13.2	60.0	95.3
P4616	serous	11.4	25.0	59.1	95.5
P4684	serous	15.8	20.3	59.2	95.3
P5390	clear cell	10.2	9.8	75.4	95.1
P7820	serous	11.8	24.7	58.8	95.4
