# Supplementary material for: Identification of Candidate Growth Promoting Genes in Ovarian Cancer through Integrated Copy Number and Expression Analysis
Source: PLoS One. 2010 Apr 8;5(4):e9983. doi: 10.1371/journal.pone.0009983 (PMC2851616; doi:10.1371/journal.pone.0009983)
Supplement: Table S3 — Germline copy number polymorphisms on Chr 3, 7, 8, 20. The regions/segments of copy number gain that contained one or more of these CNPs were removed or altered as displayed in Figure S1-B. The type of CNP is also displayed in the far right column. (0.05 MB PDF) [file pone.0009983.s003.pdf]

Cytoband	Chromosome	Start (bp)	End (bp)	Region.length	#samples	Type
3p12.3	3	79210490	80913526	165860	5	Del
3p24.2	3	25810048	25816531	6489	6	Amp
3p24.2	3	25739544	25817166	77253	5	Del
3p24.3	3	23616919	23694910	54575	3	Del
3q11.2	3	97838272	97840646	2375	3	Del
3q13.33	3	122559421	122644239	84827	6	Amp
3q13.33	3	122559421	122643114	83694	5	Del
3q22.1	3	134504447	134504492	46	6	Del
3q26.1	3	168660470	168696753	36289	10	Amp
3q26.1	3	168660470	168696753	36291	12	Del
3q26.33	3	181970431	181970843	413	3	Del
7p14.1	7	42305279	42308036	2758	3	Del
7q11.23	7	73675807	73683570	7764	3	Amp
7q21.11	7	83385157	83390443	5287	13	Amp
7q21.11	7	83385157	83390443	5288	15	Del
7q21.3	7	95107736	95107927	192	4	Del
7q22.3	7	106331207	106337089	5883	6	Del
7q31.1	7	108481492	108482037	546	3	Del
7q32.3	7	131698245	131713939	15695	5	Amp
7q36.3	7	155096769	155103094	6326	3	Del
8p11.1	8	43558059	43723631	165582	9	Amp
8p11.1	8	43580641	43722843	142207	7	Del
8p11.21	8	41661384	41666518	5136	6	Amp
8p12	8	31889700	31893094	3396	6	Amp
8p23.2	8	4356942	6033976	17889	4	Amp
8p23.2	8	4356942	6034068	17989	9	Del
8q12.1	8	60241288	60386830	145549	15	Amp
8q12.1	8	57095066	60386830	141393	10	Del
8q12.3	8	62500852	62507335	971	3	Amp
8q12.3	8	62494870	62549426	12235	4	Del
8q21.3	8	89422381	89425564	3185	4	Amp
8q22.2	8	100101505	100112077	10574	6	Del
8q23.1	8	107680826	107681140	315	9	Del
8q23.2	8	110700157	110724393	24242	5	Amp
8q23.2	8	110700157	110722873	22723	6	Del
8q23.3	8	113491593	113663103	34355	3	Del

8q24.13	8	122651921	122660861	8942	4	Del
8q24.21	8	129639246	129734934	95689	3	Amp
8q24.21	8	129657194	129672109	14916	3	Del
8q24.22	8	135231848	135434578	200169	4	Amp
8q24.22	8	135231868	135342195	10401	3	Del
8q24.23	8	137609964	137616428	6465	3	Amp
20p12.1	20	17332586	17456718	124149	9	Amp
20p12.1	20	17317374	17455689	110392	4	Del
