# Supplementary material for: Identification of Candidate Growth Promoting Genes in Ovarian Cancer through Integrated Copy Number and Expression Analysis
Source: PLoS One. 2010 Apr 8;5(4):e9983. doi: 10.1371/journal.pone.0009983 (PMC2851616; doi:10.1371/journal.pone.0009983)
Supplement: Table S4 — Regions of gain present in >40% of samples. This table contains genomic information for the 90 regions included in the expression analyses, i.e., all those regions that mapped to 1 or more probesets on the Human GeneST1.0 microarrays. On this microarray platform, most probesets map uniquely to a protein-coding gene. The region IDs correspond to those in Tables 2, 3, 4 and S5. (0.13 MB PDF) [file pone.0009983.s004.pdf]

Region_ID	Cytoband	Chr	Start (bp)	End (bp)	Region length	%Samples	#CN/SNP probesets
3_1	3q25.31	3	157223337	157972472	739251	40	494
3_2	3q25.32	3	158260079	159894705	1272589	40	960
3_3	3q25.32 - 3q25.33	3	159894705	159958518	63814	42	48
3_4	3q25.33	3	159958518	161005782	985573	40	684
3_5	3q25.33 - 3q26.1	3	161005782	161392058	386277	42	253
3_7	3q26.1	3	161392058	168915903	7388335	43	4426
3_9	3q26.1 - 3q26.2	3	168915903	169208732	292830	50	191
3_10	3q26.2	3	169208732	172478029	3269408	53	2065
3_11	3q26.2 - 3q26.31	3	172478029	172586020	107992	53	82
3_12	3q26.31	3	172586020	177094525	4508624	50	3122
3_13	3q26.31 - 3q26.32	3	177094525	177366431	271907	51	149
3_14	3q26.32	3	177366431	180517792	3151523	50	2110
3_15	3q26.32 - 3q26.33	3	180517792	180608287	90496	50	40
3_17	3q26.33	3	180608287	184152556	3544366	50	1970
3_19	3q26.33 - 3q27.1	3	184152556	184290998	138443	49	74
3_20	3q27.1	3	184290998	185995678	1704709	49	954
3_21	3q27.1 - 3q27.2	3	185995678	186007206	11529	50	11
3_22	3q27.2	3	186007206	187399115	1391946	51	863
3_23	3q27.2 - 3q27.3	3	187399115	187519179	120065	50	102
3_24	3q27.3	3	187519179	189378616	1858952	46	1506
3_25	3q27.3 - 3q28	3	189378616	189429907	51292	46	37
3_26	3q28	3	189429907	193766085	4334415	43	3180
3_27	3q28 - 3q29	3	193766085	193936360	170276	44	142
3_28	3q29	3	193936360	199337204	5342594	43	3091
7_1	7q34	7	141416100	141430833	13536	40	21
8_1	8q11.23	8	53389772	55544620	1565570	42	1434
8_2	8q11.23 - 8q12.1	8	55544620	55651731	107112	44	82
8_6	8q12.1	8	55651731	61696140	6040855	44	4078
8_7	8q12.1 - 8q12.2	8	61696140	61817420	121281	46	99
8_8	8q12.2	8	61817420	62337503	520100	44	381
8_9	8q12.2 - 8q12.3	8	62337503	62494870	157368	46	108
8_11	8q12.3	8	62494870	65928146	3433364	43	2102
8_14	8q12.3 - 8q13.1	8	65928146	66236864	308719	42	150
8_15	8q13.1	8	66236864	68051346	1814513	43	952
8_16	8q13.1 - 8q13.2	8	68051346	68292309	240964	42	87

8_17	8q13.2	8	68292309	70433174	2135937	42	1444
8_18	8q13.2 - 8q13.3	8	70433174	70815464	382291	43	314
8_19	8q13.3	8	70815464	73992590	2922091	43	2101
8_20	8q13.3 - 8q21.11	8	73992590	74016246	23657	42	16
8_21	8q21.11	8	74016246	78270404	4247517	42	2646
8_22	8q21.11 - 8q21.12	8	78270404	79275760	1005357	43	613
8_23	8q21.12	8	79275760	80255394	979659	44	567
8_24	8q21.12 - 8q21.13	8	80255394	80418549	163156	44	127
8_25	8q21.13	8	80418549	84683102	4264637	44	2471
8_26	8q21.13 - 8q21.2	8	84683102	85121597	438496	47	275
8_27	8q21.2	8	85121597	87055390	1933813	47	1008
8_28	8q21.2 - 8q21.3	8	87055390	87250134	194745	47	138
8_30	8q21.3	8	87250134	93277536	6027501	50	3573
8_32	8q21.3 - 8q22.1	8	93277536	93587044	309509	54	163
8_33	8q22.1	8	93587044	98636746	5049839	51	3383
8_34	8q22.1 - 8q22.2	8	98636746	99158628	521883	51	330
8_36	8q22.2	8	99158628	101579497	2420915	51	1215
8_38	8q22.2 - 8q22.3	8	101579497	101675295	95799	53	52
8_39	8q22.3	8	101675295	105906496	4231356	53	2760
8_40	8q22.3 - 8q23.1	8	105906496	106109752	203257	57	136
8_42	8q23.1	8	106109752	110578384	4468693	56	2859
8_45	8q23.1 - 8q23.2	8	110578384	110759734	181351	54	125
8_48	8q23.2	8	110759734	112170690	1410978	53	749
8_49	8q23.2 - 8q23.3	8	112170690	112351107	180418	54	106
8_51	8q23.3	8	112351107	117486703	5126896	54	3013
8_53	8q23.3 - 8q24.11	8	117486703	117712954	226252	57	174
8_54	8q24.11	8	117712954	119185600	1472671	57	1028
8_55	8q24.11 - 8q24.12	8	119185600	119298314	112715	57	97
8_56	8q24.12	8	119298314	121982811	2684590	57	1924
8_58	8q24.12 - 8q24.13	8	121982811	122934934	952124	58	655
8_60	8q24.13	8	122934934	127209335	4274513	60	3024
8_61	8q24.13 - 8q24.21	8	127209335	127319855	110521	63	74
8_63	8q24.21	8	127319855	131498905	4179160	61	3041
8_67	8q24.21 - 8q24.22	8	131498905	131595877	96973	61	69
8_69	8q24.22	8	131595877	136466442	4870682	60	4090
8_73	8q24.22 - 8q24.23	8	136466442	136568349	101908	61	70
8_75	8q24.23	8	136568349	139943689	3375426	60	2793

8_77	8q24.23 - 8q24.3	8	139943689	140056011	112323	60	90
8_78	8q24.3	8	140056011	146268947	6208543	60	3342
20_1	20q11.21	20	29299120	31464970	2128132	42	1188
20_2	20q11.21 - 20q11.22	20	31466294	31647667	181374	40	60
20_4	20q11.22	20	31648845	33759769	1554149	40	964
20_6	20q11.23	20	33957942	37048849	1984152	40	1822
20_7	20q12	20	37107381	41095146	3924255	42	3115
20_8	20q12 - 20q13.11	20	41095146	41113240	18095	40	20
20_9	20q13.11	20	41123607	41225982	102378	40	105
20_10	20q13.12	20	42961935	45771727	1399743	42	2009
20_11	20q13.12 - 20q13.13	20	45771821	45835796	63976	40	37
20_12	20q13.13	20	45849914	49179606	1142528	40	2385
20_13	20q13.13 - 20q13.2	20	49179606	49222255	42650	42	44
20_14	20q13.2	20	49222255	54379233	5152309	43	4211
20_15	20q13.2 - 20q13.31	20	54379233	54417168	37936	42	31
20_16	20q13.31	20	54417168	55828083	1407348	42	1146
20_17	20q13.31 - 20q13.32	20	55828083	55991018	162936	44	166
20_18	20q13.32	20	55991018	57887358	1896377	43	1455
20_19	20q13.32 - 20q13.33	20	57887358	57900659	13302	43	6
20_20	20q13.33	20	57900659	62426585	4399113	46	2909
