# Supplementary material for: Identification of Candidate Growth Promoting Genes in Ovarian Cancer through Integrated Copy Number and Expression Analysis
Source: PLoS One. 2010 Apr 8;5(4):e9983. doi: 10.1371/journal.pone.0009983 (PMC2851616; doi:10.1371/journal.pone.0009983)
Supplement: Table S5 — All differentially expressed probesets in frequent regions of gain. Every probeset tested for differential expression is listed and tagged by the region it belongs to. These region IDs are consistent across all tables in the paper and are derived as shown in Figure S1-A. Column 5 displays the Pearson's correlation between copy number and expression for the listed probeset. Columns 6–11 are derived from differential expression analyses performed using the “limma” package in R. (0.06 MB PDF) [file pone.0009983.s005.pdf]

Region-name	Expression-Probeset-ID	Gene	Number-of-CN-probesets	Pearson-correlation	logFC	AveExpr	t	P.Value	adj.P.Val	-logP
8_9	8146618	RLBP1L1	167	0.75	-	6.18	-	-	1.3E-04	12.89
8_8	8146579	CHD7	146	0.68	-	8.12	-	-	6.6E-04	10.57
8_78	8148674	MAPK15	0	#N/A	0.29	7.76	2.69	8.9E-03	1.3E-02	6.28
8_78	8148796	SCXB	0	#N/A	0.46	6.47	3.89	2.3E-04	4.4E-04	11.15
8_78	8148821	ENST00000340210	0	#N/A	0.46	6.47	3.89	2.3E-04	4.4E-04	11.15
8_78	8148772	C8orf30A	0	#N/A	0.24	7.82	4.34	4.5E-05	9.8E-05	13.32
8_78	8148799	C8orf30A	0	#N/A	0.29	7.72	5.04	3.3E-06	1.1E-05	16.45
8_78	8148808	KIAA1833	0	#N/A	0.49	7.45	5.86	1.3E-07	7.9E-07	20.27
8_78	8153664	BOP1	0	#N/A	0.41	7.80	6.41	1.4E-08	1.7E-07	22.51
8_78	8148941	ZNF7	27	0.80	0.39	7.16	6.54	8.0E-09	1.4E-07	22.79
8_78	8148658	ZNF623	18	0.72	0.68	7.40	5.81	1.6E-07	8.3E-07	20.20
8_78	8153411	ZC3H3	34	0.72	0.49	7.15	7.28	3.5E-10	4.2E-08	24.49
8_78	8153175	NIBP	496	0.70	0.41	8.01	4.91	5.5E-06	1.6E-05	15.98
8_78	8148467	CHRAC1	7	0.69	0.46	7.40	6.12	4.6E-08	3.5E-07	21.45
8_78	8153876	KIAA1688	30	0.68	0.43	6.98	5.81	1.6E-07	8.3E-07	20.20
8_78	8153390	TOP1MT	26	0.68	0.43	7.73	4.53	2.3E-05	5.4E-05	14.18
8_78	8153819	CYHR1	18	0.67	0.84	7.15	6.75	3.3E-09	1.1E-07	23.10
8_78	8148694	GRINA	5	0.67	0.63	8.13	6.17	3.7E-08	3.5E-07	21.45
8_78	8153896	ZNF34	17	0.67	0.34	6.30	5.36	9.9E-07	4.1E-06	17.89
8_78	8153652	SHARPIN	10	0.67	0.44	8.39	6.48	1.0E-08	1.5E-07	22.64
8_78	8153449	EEF1D	10	0.67	0.39	9.35	3.47	8.9E-04	1.5E-03	9.35
8_78	8153223	PTK2	207	0.67	0.45	8.87	3.67	4.7E-04	8.5E-04	10.20
8_78	8148783	KIAA1833	44	0.66	0.67	7.91	4.98	4.2E-06	1.2E-05	16.30
8_78	8153609	PARP10	5	0.66	0.43	7.59	6.13	4.4E-08	3.5E-07	21.45
8_78	8153536	PUF60	8	0.66	0.72	8.87	6.93	1.6E-09	9.4E-08	23.34
8_78	8148662	ZNF707	14	0.66	0.47	6.80	6.14	4.2E-08	3.5E-07	21.45
8_78	8153727	CPSF1	7	0.65	0.57	7.93	5.16	2.1E-06	7.8E-06	16.96
8_78	8153920	ZNF250	31	0.65	0.53	7.46	5.32	1.1E-06	4.6E-06	17.73
8_78	8148824	HSF1	14	0.64	0.54	8.38	6.57	7.0E-09	1.4E-07	22.79
8_78	8153678	BOP1	9	0.64	0.39	7.74	4.99	4.1E-06	1.2E-05	16.30
8_78	8148955	C8orf33	11	0.63	0.61	7.92	6.31	2.1E-08	2.3E-07	22.08
8_78	8148597	ZFP41	9	0.62	0.34	6.71	4.92	5.5E-06	1.6E-05	15.98
8_78	8148841	GPR172A	4	0.62	0.53	8.30	6.72	3.7E-09	1.1E-07	23.10
8_78	8153457	AY358690	7	0.61	0.36	7.00	4.88	6.3E-06	1.7E-05	15.81
8_78	8153568	PLEC1	11	0.61	0.56	8.51	4.57	2.0E-05	5.0E-05	14.30
8_78	8153911	COMMD5	26	0.61	0.26	7.42	5.12	2.5E-06	8.6E-06	16.83
8_78	8153497	SCRIB	8	0.61	0.53	7.73	6.62	5.6E-09	1.4E-07	22.81
8_78	8153488	FAM83H	3	0.61	0.57	7.48	5.91	1.1E-07	6.8E-07	20.48
8_78	8148715	GPAA1	13	0.61	0.56	8.27	5.50	5.7E-07	2.6E-06	18.55
8_78	8148642	GSDMDC1	8	0.60	0.46	7.18	6.12	4.5E-08	3.5E-07	21.45
8_78	8148476	DENND3	67	0.60	0.35	7.02	4.21	7.4E-05	1.6E-04	12.63
8_78	8148728	CYC1	10	0.60	0.89	8.58	6.03	6.7E-08	4.8E-07	21.00
8_78	8153328	JRK	16	0.60	0.26	7.72	4.17	8.4E-05	1.7E-04	12.49
8_78	8153304	TSNARE1	103	0.59	0.32	7.18	4.67	1.4E-05	3.5E-05	14.81
8_78	8148737	MAF1	5	0.58	0.53	8.11	6.45	1.1E-08	1.5E-07	22.64
8_78	8153201	EIF2C2	60	0.57	0.50	7.89	3.95	1.8E-04	3.7E-04	11.40
8_78	8148710	EXOSC4	11	0.57	0.32	7.36	5.01	3.8E-06	1.2E-05	16.31
8_78	8153709	FBXL6	11	0.56	0.30	6.91	4.45	3.1E-05	6.8E-05	13.85
8_78	8148501	PTP4A3	4	0.56	0.64	8.08	5.14	2.4E-06	8.4E-06	16.87
8_78	8153935	ZNF252	18	0.56	0.65	6.25	3.87	2.4E-04	4.6E-04	11.08
8_78	8153550	NRBP2	12	0.53	0.55	7.76	3.84	2.6E-04	5.0E-04	10.98
8_78	8148515	---	17	0.53	0.23	5.02	2.38	2.0E-02	2.8E-02	5.18
8_78	8153474	TSTA3	17	0.53	0.73	8.36	4.83	7.6E-06	2.1E-05	15.57
8_78	8153903	RPL8	13	0.53	0.48	10.49	3.25	1.8E-03	2.9E-03	8.44
8_78	8148559	C8orf55	9	0.53	0.41	6.86	5.21	1.8E-06	6.9E-06	17.15
8_78	8148572	LY6E	9	0.53	1.09	9.38	4.99	4.2E-06	1.2E-05	16.30
8_78	8153823	CYHR1	11	0.52	0.36	7.43	5.18	2.0E-06	7.4E-06	17.04
8_78	8153459	PYCRL	10	0.51	0.29	7.16	4.79	8.9E-06	2.4E-05	15.38
8_78	8153790	NFKBIL2	13	0.51	0.29	7.18	4.59	1.9E-05	4.8E-05	14.36
8_78	8153625	OPLAH	4	0.51	0.44	6.99	5.56	4.4E-07	2.1E-06	18.83
8_78	8148888	PPP1R16A	2	0.50	0.37	7.66	5.00	4.0E-06	1.2E-05	16.30
8_78	8153776	VPS28	3	0.50	0.61	8.00	5.93	9.9E-08	6.7E-07	20.52
8_78	8148867	KIFC2	16	0.50	0.38	7.25	4.50	2.6E-05	5.8E-05	14.06
8_78	8148621	RHPN1	7	0.49	0.30	7.68	3.81	3.0E-04	5.5E-04	10.83
8_78	8148692	ENST00000355099	8	0.48	0.24	6.68	3.02	3.5E-03	5.3E-03	7.56
8_78	8153684	DGAT1	5	0.48	0.51	7.97	5.36	9.8E-07	4.1E-06	17.89
8_78	8153890	ENST00000292562	13	0.47	0.35	7.49	3.40	1.1E-03	1.9E-03	9.06
8_78	8148615	ZNF696	14	0.47	0.20	7.04	3.19	2.1E-03	3.3E-03	8.24
8_78	8148923	LRRC14	1	0.47	0.42	7.57	5.84	1.4E-07	8.1E-07	20.24
8_78	8153405	C8orf51	7	0.46	0.23	6.76	3.34	1.3E-03	2.2E-03	8.82
8_78	8148917	MFSD3	1	0.46	0.43	7.57	5.57	4.2E-07	2.1E-06	18.85
8_78	8148850	ADCK5	13	0.45	0.25	7.07	4.48	2.8E-05	6.3E-05	13.95
8_78	8153868	ENST00000313465	2	0.43	0.26	7.23	4.10	1.1E-04	2.2E-04	12.13
8_78	8148671	BREA2	10	0.42	0.23	6.63	2.87	5.4E-03	8.1E-03	6.94
8_78	8153258	ENST00000377746	12	0.41	0.74	6.99	4.56	2.1E-05	5.0E-05	14.30
8_78	8148553	LY6K	10	0.40	0.22	6.54	2.76	7.3E-03	1.1E-02	6.54
8_78	8148580	C8orf31	12	0.40	0.24	6.74	2.86	5.6E-03	8.4E-03	6.90
8_78	8148607	GLI4	8	0.39	0.20	7.08	3.26	1.7E-03	2.8E-03	8.47
8_78	8153262	SLC45A4	25	0.38	0.62	7.49	4.54	2.2E-05	5.3E-05	14.21
8_78	8148748	KIAA1875	12	0.37	0.20	6.62	2.70	8.6E-03	1.3E-02	6.31
8_78	8153346	LYNX1	9	0.37	0.24	7.58	2.59	1.1E-02	1.6E-02	5.93
8_78	8153930	ZNF16	32	0.36	0.38	6.90	5.49	5.8E-07	2.6E-06	18.55
8_78	8153835	ENST00000355148	4	0.35	0.28	6.64	3.20	2.0E-03	3.2E-03	8.28
8_78	8153838	RECQL4	1	0.35	0.25	7.30	3.63	5.3E-04	9.6E-04	10.02
8_78	8153363	CYP11B1	16	0.35	0.20	6.99	2.31	2.4E-02	3.3E-02	4.92
8_78	8148640	---	6	0.34	0.17	7.33	2.45	1.7E-02	2.4E-02	5.39
8_78	8153430	NAPRT1	8	0.34	0.23	7.64	3.34	1.3E-03	2.2E-03	8.82
8_78	8148951	C8orf77	16	0.33	0.16	6.49	2.27	2.7E-02	3.6E-02	4.79
8_78	8148655	TIGD5	6	0.31	0.21	7.09	3.58	6.1E-04	1.1E-03	9.86
8_78	8153762	SLC39A4	9	0.31	0.23	7.57	3.06	3.2E-03	4.9E-03	7.68
8_78	8153424	C8orf73	4	0.15	0.49	6.57	3.59	6.0E-04	1.1E-03	9.87
8_77	8153101	COL22A1	326	0.36	-	7.22	-	-	6.4E-03	7.28
8_76	8153101	COL22A1	326	0.36	0.23	7.22	3.15	2.1E-03	4.2E-03	7.89
8_76	8153071	FAM135B	355	0.35	0.23	5.84	2.71	7.7E-03	7.7E-03	7.02
8_75	8153069	---	15	0.37	0.16	4.26	2.58	1.2E-02	2.4E-02	5.37
8_72	8153043	ZFAT1	236	0.62	0.39	6.71	5.75	1.8E-07	5.3E-07	20.84
8_72	8153067	hsa-mir-30d	10	0.38	0.16	4.11	2.92	4.6E-03	6.9E-03	7.19
8_7	8146579	CHD7	146	0.68	0.69	8.08	3.78	1.6E-04	1.6E-04	12.62
8_7	8146564	RAB2A	76	0.65	0.85	8.54	4.67	3.0E-06	6.0E-06	17.34
8_69	8148358	PHF20L1	52	0.57	0.37	6.80	2.92	4.8E-03	1.6E-02	5.94
8_69	8148385	TG	298	0.48	0.26	6.38	3.26	1.7E-03	1.5E-02	6.09
8_69	8148435	WISP1	69	0.45	0.27	7.07	2.86	5.7E-03	1.6E-02	5.94
8_69	8148333	KIAA0143	85	0.42	0.51	8.42	2.87	5.5E-03	1.6E-02	5.94
8_69	8152924	OC90	46	0.39	0.26	6.41	3.09	2.9E-03	1.5E-02	6.09
8_69	8152946	KCNQ3	326	0.39	0.22	6.94	3.11	2.7E-03	1.5E-02	6.09

8_69	8153002	NDRG1	73	0.39	0.72	9.22	3.33	1.4E-03	1.5E-02	6.09
8_69	8152988	SLA	107	0.33	0.34	6.19	2.62	1.1E-02	2.7E-02	5.19
8_69	8152938	ENST00000262283	29	0.27	0.20	5.72	2.51	1.4E-02	3.2E-02	4.96
8_66	8152900	ENST00000363434	11	0.55	0.25	5.09	2.56	1.3E-02	3.3E-02	4.91
8_66	8152845	FAM49B	83	0.48	0.60	7.30	3.72	4.1E-04	3.2E-03	8.27
8_66	8152867	DDEF1	236	0.46	0.36	7.86	2.40	1.9E-02	3.8E-02	4.71
8_66	8152828	MLZE	65	0.43	0.54	5.48	2.80	6.6E-03	2.6E-02	5.24
8_63	8152812	FAM84B	12	0.64	0.61	6.96	3.54	6.9E-04	5.5E-03	7.51
8_63	8152819	---	21	0.34	0.29	6.49	2.66	9.4E-03	3.8E-02	4.73
8_60	8152648	C8orf76	14	0.79	0.56	6.37	6.01	8.0E-08	3.2E-06	18.26
8_60	8152628	DERL1	31	0.70	0.90	8.12	5.63	3.6E-07	4.8E-06	17.67
8_60	8148198	C8orf32	34	0.68	0.53	6.41	4.69	1.3E-05	5.9E-05	14.05
8_60	8148263	TRMT12	11	0.68	0.61	6.66	5.31	1.3E-06	1.0E-05	16.57
8_60	8148265	RNF139	19	0.66	0.56	7.21	5.78	2.0E-07	3.9E-06	17.95
8_60	8148158	WDR67	78	0.65	0.51	6.18	4.10	1.1E-04	3.7E-04	11.40
8_60	8152750	TMEM65	34	0.64	0.52	7.05	4.75	1.1E-05	5.3E-05	14.20
8_60	8148280	SQLE	22	0.62	0.85	8.16	4.60	1.9E-05	7.5E-05	13.71
8_60	8152668	ATAD2	33	0.61	1.04	7.53	5.48	6.5E-07	6.5E-06	17.22
8_60	8148270	NDUFB9	23	0.60	0.77	8.08	5.09	3.0E-06	2.0E-05	15.62
8_60	8148293	NSMCE2	165	0.58	0.58	6.84	4.42	3.7E-05	1.3E-04	12.87
8_60	8148276	ZNF572	13	0.57	0.48	6.06	4.77	1.0E-05	5.3E-05	14.20
8_60	8152656	ZHX1	20	0.55	0.42	6.85	3.52	7.8E-04	1.8E-03	9.09
8_60	8152782	KIAA0196	67	0.55	0.55	7.37	3.84	2.7E-04	8.3E-04	10.23
8_60	8148184	FAM83A	23	0.54	0.49	6.78	3.59	6.1E-04	1.7E-03	9.23
8_60	8148208	FAM91A1	25	0.54	0.62	8.01	3.59	6.2E-04	1.7E-03	9.23
8_60	8152664	---	12	0.53	0.39	4.57	3.55	6.9E-04	1.7E-03	9.17
8_60	8152759	TATDN1	31	0.52	0.18	6.77	2.45	1.7E-02	3.0E-02	5.04
8_60	8152703	FBXO32	39	0.50	0.74	7.73	3.31	1.5E-03	3.1E-03	8.33
8_60	8152666	---	8	0.49	0.30	5.48	3.37	1.2E-03	2.7E-03	8.53
8_60	8152642	AY225521	6	0.35	0.22	6.20	3.01	3.7E-03	7.3E-03	7.09
8_60	8152626	---	16	0.34	0.19	5.12	2.31	2.4E-02	4.0E-02	4.64
8_60	8148194	ENST00000390925	10	0.33	0.18	4.38	2.42	1.8E-02	3.2E-02	4.98
8_60	8148261	---	20	0.20	0.17	5.07	2.62	1.1E-02	2.0E-02	5.62
8_6	8146564	RAB2A	76	0.65	0.80	8.55	4.15	8.4E-05	1.7E-04	12.55
8_59	8152617	HAS2	36	0.27	-	6.35	-	-	3.4E-02	4.90
8_56	8152553	TAF2	61	0.62	0.49	7.66	3.50	8.4E-04	4.8E-03	7.71
8_56	8152597	MRPL13	28	0.56	0.31	5.01	3.22	2.0E-03	8.5E-03	6.88
8_56	8148124	MTBP	45	0.54	0.66	5.69	4.04	1.4E-04	1.2E-03	9.73
8_56	8152506	SAMD12	221	0.53	0.56	6.66	3.05	3.3E-03	1.1E-02	6.48
8_56	8152582	DCC1	20	0.52	0.55	5.82	4.24	7.1E-05	1.2E-03	9.73
8_56	8148040	MAL2	43	0.49	0.75	9.97	2.98	4.0E-03	1.1E-02	6.47
8_55	8152491	EXT1	256	0.58	-	7.70	-	-	4.8E-05	14.33
8_54	8147994	C8orf53	29	0.72	0.45	6.85	3.97	1.7E-04	6.3E-04	10.64
8_54	8148022	MED30	37	0.66	0.19	5.21	3.92	2.1E-04	6.3E-04	10.64
8_54	8152477	RAD21	18	0.60	0.71	9.00	4.24	7.0E-05	6.3E-04	10.64
8_54	8152491	EXT1	256	0.58	0.39	7.70	3.22	2.0E-03	4.0E-03	7.96
8_54	8152465	EIF3H	73	0.57	0.38	8.90	2.85	5.7E-03	8.6E-03	6.86
8_54	8148000	ENST00000378279	19	0.47	0.23	6.18	3.18	2.2E-03	4.0E-03	7.96
8_54	8148003	SLC30A8	159	0.29	0.23	5.58	2.55	1.3E-02	1.7E-02	5.89
8_54	8148018	---	13	0.19	0.20	5.79	2.49	1.5E-02	1.7E-02	5.86
8_52	8152453	TRPS1	256	0.51	0.83	7.98	2.87	5.6E-03	2.2E-02	5.49
8_5	8146482	TGS1	31	0.80	0.49	6.96	3.70	4.4E-04	3.3E-03	8.25
8_5	8150846	TMEM68	26	0.78	0.51	6.28	3.47	9.0E-04	3.3E-03	8.25
8_5	8150866	---	10	0.73	0.22	5.40	2.42	1.8E-02	3.0E-02	5.06
8_5	8146475	XKR4	380	0.63	0.30	6.87	3.44	9.8E-04	3.3E-03	8.25
8_5	8146468	RP1	25	0.45	0.33	5.02	2.43	1.8E-02	3.0E-02	5.06
8_5	8150860	---	9	0.38	0.21	7.81	2.83	6.1E-03	1.5E-02	6.04
8_47	8152355	GOLSYN	77	0.35	-	6.83	-	-	9.5E-03	6.72
8_45	8147970	EBAG9	25	0.73	0.68	6.83	6.42	1.8E-08	5.3E-08	24.17
8_45	8152355	GOLSYN	77	0.35	0.32	6.83	2.67	9.5E-03	1.4E-02	6.13
8_43	8147864	TTC35	46	0.62	0.61	7.61	3.81	3.1E-04	1.3E-03	9.54
8_43	8152340	NUDCD1	55	0.62	0.58	6.94	3.90	2.3E-04	1.3E-03	9.54
8_43	8147883	ENY2	16	0.62	0.62	7.76	4.30	5.7E-05	7.4E-04	10.39
8_43	8147848	OXR1	157	0.58	0.61	7.01	3.48	8.8E-04	2.9E-03	8.45
8_42	8147848	OXR1	157	0.58	0.51	7.01	2.84	6.0E-03	1.8E-02	5.80
8_39	8152096	YWHAZ	23	0.70	0.60	9.86	4.91	6.2E-06	3.1E-04	11.67
8_39	8147697	GRHL2	193	0.70	0.64	7.68	3.24	1.9E-03	5.8E-03	7.42
8_39	8152117	---	20	0.67	0.21	5.87	2.64	1.0E-02	2.5E-02	5.32
8_39	8152111	ZNF706	21	0.65	0.35	7.51	4.22	7.6E-05	1.0E-03	9.94
8_39	8152148	UBR5	64	0.64	0.58	8.60	4.19	8.4E-05	1.0E-03	9.94
8_39	8147724	ATP6V1C1	50	0.62	0.71	7.71	4.11	1.1E-04	1.0E-03	9.94
8_39	8152222	AZIN1	30	0.61	0.62	8.41	3.68	4.7E-04	2.6E-03	8.58
8_39	8147766	FZD6	25	0.60	0.70	7.71	3.85	2.7E-04	1.7E-03	9.23
8_39	8152079	PABPC1	17	0.58	0.59	11.30	3.86	2.6E-04	1.7E-03	9.23
8_39	8147785	WDSOF1	31	0.57	0.40	7.69	4.52	2.6E-05	6.5E-04	10.59
8_39	8152255	SLC25A32	19	0.57	0.42	7.69	3.38	1.2E-03	4.4E-03	7.84
8_39	8152280	LRP12	50	0.54	0.39	6.21	2.61	1.1E-02	2.5E-02	5.32
8_39	8152090	ENST00000386147	8	0.52	0.23	4.37	3.47	9.3E-04	3.9E-03	8.01
8_39	8147691	ENST00000365490	14	0.51	0.23	5.74	3.24	1.9E-03	5.8E-03	7.42
8_39	8152119	NCALD	332	0.46	0.53	7.39	2.72	8.4E-03	2.2E-02	5.50
8_39	8147721	FLJ45248	16	0.45	0.26	6.12	4.08	1.2E-04	1.0E-03	9.94
8_39	8152133	RRM2B	28	0.45	0.44	7.26	3.53	7.6E-04	3.5E-03	8.18
8_39	8147777	CTHRC1	17	0.44	0.30	7.13	2.63	1.0E-02	2.5E-02	5.32
8_39	8147828	---	11	0.41	0.20	4.10	3.38	1.2E-03	4.4E-03	7.84
8_39	8152248	ENST00000386036	14	0.40	0.25	4.48	3.10	2.8E-03	8.3E-03	6.92
8_39	8152211	---	14	0.40	0.48	4.91	2.80	6.6E-03	1.8E-02	5.77
8_39	8152062	MGC39715	80	0.39	0.45	5.76	3.55	7.1E-04	3.5E-03	8.18
8_39	8147830	TM7SF4	28	0.33	0.25	5.48	2.43	1.8E-02	3.9E-02	4.70
8_39	8152213	---	17	0.32	0.26	5.78	2.31	2.4E-02	4.7E-02	4.40
8_39	8147689	---	10	0.29	0.18	6.27	2.40	1.9E-02	4.0E-02	4.64
8_38	8152053	ANKRD46	41	0.69	0.42	6.74	4.13	8.1E-05	1.6E-04	12.60
8_38	8152062	MGC39715	80	0.39	0.47	5.76	3.83	2.4E-04	2.4E-04	12.03
8_37	8151993	COX6C	15	0.64	0.72	8.87	4.95	5.3E-06	4.9E-05	14.32
8_37	8152041	RNF19A	35	0.63	0.75	8.26	4.81	8.9E-06	4.9E-05	14.32
8_37	8147654	POLR2K	8	0.53	0.63	7.78	4.38	4.2E-05	1.5E-04	12.67
8_37	8147580	VPS13B	413	0.43	0.40	7.72	3.15	2.4E-03	5.4E-03	7.54
8_37	8152031	FBXO43	13	0.35	0.23	5.22	3.16	2.3E-03	5.4E-03	7.54
8_37	8147650	---	11	0.24	0.18	4.45	2.50	1.5E-02	2.7E-02	5.21
8_36	8147548	POP1	41	0.70	0.35	6.06	4.29	5.5E-05	3.0E-04	11.68
8_36	8151952	NPAL2	80	0.57	0.66	6.68	4.30	5.5E-05	3.0E-04	11.68
8_36	8151967	STK3	148	0.56	0.48	6.91	4.02	1.4E-04	5.3E-04	10.89
8_36	8151942	HRSP12	22	0.50	0.47	6.88	3.10	2.8E-03	6.2E-03	7.34
8_36	8147580	VPS13B	413	0.43	0.40	7.72	3.18	2.2E-03	6.0E-03	7.38
8_36	8151989	---	7	0.38	0.21	5.17	2.86	5.5E-03	8.7E-03	6.84
8_36	8147543	ENST00000318528	39	0.27	0.22	6.20	2.74	7.8E-03	1.1E-02	6.54
8_36	8147566	KCNS2	19	0.25	0.15	7.04	2.28	2.6E-02	3.1E-02	4.99

8_36	8147573	OSR2	8	0.23	0.26	6.41	2.89	5.2E-03	8.7E-03	6.84
8_34	8147483	MTDH	46	0.72	0.66	8.64	5.49	6.9E-07	5.5E-06	17.47
8_34	8147503	LAPTM4B	87	0.57	0.74	9.10	3.22	2.0E-03	3.3E-03	8.24
8_34	8151935	RPL30	10	0.42	0.48	10.92	3.30	1.6E-03	3.3E-03	8.24
8_34	8147516	MATN2	117	0.39	0.59	7.20	3.17	2.3E-03	3.3E-03	8.24
8_34	8147541	---	7	0.30	0.17	4.56	3.25	1.8E-03	3.3E-03	8.24
8_34	8147543	ENST00000318528	39	0.27	0.22	6.20	2.78	7.1E-03	8.1E-03	6.95
8_34	8147501	---	7	0.24	0.26	4.64	3.15	2.5E-03	3.3E-03	8.24
8_33	8151842	KIAA1429	72	0.70	0.43	7.33	3.16	2.3E-03	8.3E-03	6.92
8_33	8151909	UQCRB	15	0.69	0.41	5.53	4.45	3.3E-05	1.1E-03	9.89
8_33	8151917	MTERFD1	21	0.68	0.46	6.31	3.83	2.8E-04	2.2E-03	8.80
8_33	8147424	C8orf38	67	0.65	0.36	6.27	3.84	2.8E-04	2.2E-03	8.80
8_33	8147447	PTDSS1	64	0.62	0.57	8.03	3.47	9.0E-04	3.6E-03	8.12
8_33	8147351	RBM35A	57	0.62	0.65	8.99	2.99	3.9E-03	1.1E-02	6.48
8_33	8147439	PLEKHF2	24	0.57	0.62	6.71	3.62	5.6E-04	2.8E-03	8.46
8_33	8151788	RBM12B	13	0.56	0.29	7.48	2.46	1.6E-02	3.3E-02	4.93
8_33	8147373	BC071775	20	0.54	0.49	6.06	3.59	6.2E-04	2.8E-03	8.46
8_33	8147396	INTS8	35	0.54	0.41	6.92	3.00	3.8E-03	1.1E-02	6.48
8_33	8151890	TP53INP1	33	0.52	0.62	8.07	4.06	1.3E-04	2.0E-03	8.93
8_33	8147344	PPM2C	18	0.52	0.29	8.22	2.29	2.5E-02	4.7E-02	4.41
8_33	8147371	---	8	0.48	0.27	6.51	3.71	4.3E-04	2.7E-03	8.52
8_33	8151931	TSPYL5	21	0.42	0.57	7.03	2.91	4.9E-03	1.3E-02	6.27
8_33	8151888	---	14	0.36	0.33	4.99	2.59	1.2E-02	2.7E-02	5.20
8_33	8151927	---	16	0.35	0.33	6.14	2.68	9.3E-03	2.3E-02	5.44
8_33	8151816	GEM	34	0.22	0.25	6.45	2.50	1.5E-02	3.2E-02	4.98
8_31	8147262	OTUD6B	18	0.60	0.43	7.17	4.12	1.1E-04	1.2E-03	9.74
8_31	8151711	NBN	52	0.57	0.51	7.40	3.29	1.6E-03	8.0E-03	6.97
8_31	8151756	TMEM55A	29	0.46	0.34	6.17	3.34	1.4E-03	8.0E-03	6.97
8_31	8151747	TMEM64	119	0.41	0.23	7.05	2.63	1.1E-02	3.0E-02	5.06
8_31	8147273	SLC26A7	185	0.40	0.57	5.32	3.11	2.7E-03	1.1E-02	6.52
8_31	8147242	---	6	0.35	0.40	4.87	4.09	1.2E-04	1.2E-03	9.74
8_31	8151768	RUNX1T1	172	0.27	0.28	5.38	2.78	7.0E-03	2.3E-02	5.42
8_30	8147172	CPNE3	51	0.66	0.69	7.97	4.18	8.3E-05	2.8E-04	11.82
8_30	8151644	FAM82B	21	0.62	0.48	6.49	4.34	4.7E-05	2.3E-04	12.06
8_30	8147156	WWP1	43	0.61	0.47	8.15	4.57	2.0E-05	2.0E-04	12.28
8_30	8151659	CNGB3	131	0.38	0.17	4.97	2.58	1.2E-02	2.4E-02	5.37
8_30	8151679	WDR21C	10	0.37	0.30	6.03	3.74	3.8E-04	9.4E-04	10.05
8_27	8151613	ENST00000379003	0	#N/A	0.54	10.70	2.71	8.5E-03	1.4E-02	6.21
8_27	8151621	ENST00000379010	0	#N/A	0.54	10.70	2.71	8.5E-03	1.4E-02	6.21
8_27	8151609	REXO1L1	0	#N/A	0.55	9.75	3.15	2.4E-03	6.4E-03	7.28
8_27	8151623	REXO1L2P	0	#N/A	0.54	9.87	3.30	1.6E-03	4.8E-03	7.71
8_27	8151587	C8orf59	9	0.48	0.57	6.50	4.76	1.1E-05	2.6E-04	11.92
8_27	8147101	E2F5	25	0.45	0.40	6.94	2.42	1.8E-02	2.7E-02	5.21
8_27	8151631	ENST00000378995	20	0.38	0.56	9.81	3.34	1.4E-03	4.8E-03	7.71
8_27	8151603	REXO1L2P	7	0.36	0.54	9.87	3.30	1.6E-03	4.8E-03	7.71
8_27	8151629	ENST00000379010	18	0.27	0.59	10.57	2.76	7.5E-03	1.4E-02	6.21
8_27	8151617	ENST00000379003	1	0.04	0.54	10.70	2.71	8.5E-03	1.4E-02	6.21
8_27	8151619	REXO1L2P	1	0.01	0.55	9.91	3.29	1.6E-03	4.8E-03	7.71
8_27	8151615	REXO1L2P	1	0.00	0.55	9.80	3.31	1.5E-03	4.8E-03	7.71
8_27	8151605	ENST00000379006	1	-0.01	0.54	10.70	2.71	8.5E-03	1.4E-02	6.21
8_27	8151607	REXO1L2P	1	-0.06	0.54	9.87	3.30	1.6E-03	4.8E-03	7.71
8_27	8151627	REXO1L2P	3	-0.14	0.57	9.87	3.32	1.5E-03	4.8E-03	7.71
8_27	8151625	ENST00000379006	3	-0.19	0.54	10.70	2.71	8.5E-03	1.4E-02	6.21
8_25	8147057	CHMP4C	25	0.58	0.31	7.11	3.73	4.0E-04	9.9E-03	6.65
8_25	8151561	ZFAND1	15	0.54	0.36	7.97	2.53	1.4E-02	4.3E-02	4.55
8_25	8151496	ZNF704	119	0.54	0.55	7.67	3.36	1.3E-03	1.6E-02	5.95
8_25	8151542	ENST00000360464	16	0.44	0.15	4.19	2.41	1.9E-02	5.0E-02	4.33
8_25	8151508	ENST00000391127	7	0.43	0.19	5.18	2.55	1.3E-02	4.3E-02	4.55
8_25	8147040	ZBTB10	23	0.42	0.40	7.76	3.11	2.7E-03	2.2E-02	5.48
8_25	8151490	ENST00000383922	12	0.42	0.13	3.17	2.67	9.5E-03	4.3E-02	4.55
8_25	8151572	SNX16	25	0.42	0.45	5.46	3.01	3.6E-03	2.3E-02	5.47
8_25	8147038	---	12	0.38	0.21	4.81	2.39	2.0E-02	5.0E-02	4.33
8_25	8151492	---	15	0.33	0.15	4.86	2.63	1.0E-02	4.3E-02	4.55
8_21	8146930	TMEM70	20	0.64	0.26	6.61	3.91	2.2E-04	2.7E-03	8.53
8_21	8151384	STAU2	212	0.64	0.39	6.88	3.79	3.2E-04	2.7E-03	8.53
8_21	8151401	UBE2W	56	0.50	0.35	7.16	2.73	8.0E-03	3.2E-02	4.98
8_21	8151436	PXMP3	18	0.47	0.43	6.32	3.80	3.1E-04	2.7E-03	8.53
8_21	8151413	TCEB1	28	0.47	0.17	7.00	2.64	1.0E-02	3.2E-02	4.96
8_21	8146914	TERF1	38	0.45	0.38	8.07	2.89	5.2E-03	3.2E-02	4.98
8_21	8146943	---	11	0.40	0.32	5.21	2.71	8.6E-03	3.2E-02	4.98
8_21	8151432	ENST00000388687	15	0.25	0.34	6.08	2.69	8.9E-03	3.2E-02	4.98
8_20	8146908	KCNB2	357	0.57	-	6.14	-	-	7.0E-03	7.16
8_2	8150844	ENST00000297317	16	0.68	-	6.90	-	-	1.6E-03	9.26
8_19	8151254	NCOA2	144	0.63	0.52	8.04	4.14	9.6E-05	1.1E-03	9.89
8_19	8146908	KCNB2	357	0.57	0.42	6.14	4.89	6.2E-06	1.4E-04	12.83
8_19	8151252	ENST00000334932	14	0.48	0.26	6.33	3.72	4.1E-04	3.0E-03	8.39
8_19	8151334	MSC	25	0.47	0.19	6.48	2.82	6.2E-03	1.9E-02	5.69
8_19	8151308	---	8	0.45	0.13	4.14	2.39	2.0E-02	4.8E-02	4.37
8_19	8151341	TRPA1	69	0.43	0.25	4.80	3.28	1.6E-03	7.1E-03	7.14
8_19	8151281	TRAM1	28	0.43	0.39	9.45	3.02	3.5E-03	1.3E-02	6.28
8_19	8151238	---	11	0.41	0.35	5.94	3.54	7.3E-04	4.0E-03	7.97
8_19	8151236	---	16	0.32	0.23	5.65	2.62	1.1E-02	3.0E-02	5.08
8_17	8151149	ARFGEF1	54	0.63	0.53	8.04	3.81	3.1E-04	5.2E-03	7.58
8_16	8151149	ARFGEF1	54	0.63	0.52	8.06	3.78	3.3E-04	1.0E-03	9.97
8_16	8151136	COPS5	18	0.63	0.53	6.90	4.73	1.2E-05	7.0E-05	13.80
8_16	8146756	CSPP1	53	0.48	0.54	6.70	3.09	2.9E-03	5.8E-03	7.42
8_16	8146754	---	4	0.48	0.23	4.59	2.89	5.2E-03	6.2E-03	7.34
8_16	8151127	ENST00000324682	30	0.43	0.31	3.86	2.97	4.1E-03	6.1E-03	7.35
8_16	8146788	---	9	0.37	0.42	6.33	2.42	1.8E-02	1.8E-02	5.79
8_15	8151118	VCPIP1	13	0.73	0.39	8.09	4.69	1.4E-05	1.5E-04	12.74
8_15	8151066	ARMC1	20	0.65	0.62	7.12	4.69	1.4E-05	1.5E-04	12.74
8_15	8146711	C8orf44	14	0.63	0.55	6.07	4.29	5.9E-05	4.1E-04	11.25
8_15	8146685	RRS1	12	0.62	0.44	7.25	3.90	2.2E-04	1.2E-03	9.73
8_15	8146649	MTFR1	31	0.54	0.39	7.57	2.78	7.1E-03	1.4E-02	6.21
8_15	8146647	---	13	0.53	0.43	4.31	3.46	9.4E-04	2.2E-03	8.84
8_15	8151090	---	9	0.50	0.33	5.29	3.76	3.5E-04	1.2E-03	9.71
8_15	8146717	SGK3	70	0.50	0.39	6.34	3.70	4.3E-04	1.2E-03	9.71
8_15	8146738	C8orf45	19	0.50	0.35	5.06	3.69	4.5E-04	1.2E-03	9.71
8_15	8146687	ADHFE1	38	0.49	0.39	6.15	3.03	3.5E-03	7.3E-03	7.09
8_15	8151101	MYBL1	25	0.45	0.72	5.43	3.69	4.5E-04	1.2E-03	9.71
8_15	8151127	ENST00000324682	30	0.43	0.25	3.86	2.46	1.6E-02	2.6E-02	5.24
8_15	8146703	C8orf46	23	0.39	0.19	6.14	2.66	9.9E-03	1.7E-02	5.86
8_15	8151092	CRH	22	0.37	0.19	5.09	2.30	2.5E-02	3.7E-02	4.76
8_13	8146618	RLBP1L1	167	0.75	0.52	6.18	4.17	9.0E-05	1.3E-03	9.63
8_13	8146637	YTHDF3	22	0.66	0.60	7.38	3.73	4.0E-04	2.8E-03	8.48
8_13	8146633	AK127279	15	0.46	0.33	6.52	2.79	6.9E-03	3.2E-02	4.95

8_11	8146618	RLBP1L1	167	0.75	-	6.18	-	-	1.3E-03	9.58
8_1	8150797	ATP6V1H	68	0.79	0.72	7.47	5.82	1.3E-07	2.0E-06	18.97
8_1	8150818	TCEA1	39	0.74	0.53	8.60	5.30	1.1E-06	5.4E-06	17.50
8_1	8146448	MRPL15	15	0.70	0.75	7.45	5.37	8.2E-07	5.4E-06	17.50
8_1	8146435	RGS20	63	0.68	0.31	5.87	3.49	8.0E-04	2.0E-03	8.97
8_1	8150757	RB1CC1	58	0.66	0.60	7.25	4.40	3.4E-05	1.0E-04	13.24
8_1	8146456	---	9	0.66	0.28	6.41	2.72	8.1E-03	1.5E-02	6.04
8_1	8150830	LYPLA1	26	0.58	0.64	7.62	4.60	1.6E-05	6.1E-05	14.00
8_1	8146458	---	19	0.55	0.28	4.81	2.64	1.0E-02	1.7E-02	5.88
8_1	8150751	UNQ9433	26	0.50	0.19	5.58	2.32	2.3E-02	3.1E-02	5.00
8_1	8146429	NPBWR1	13	0.47	0.26	6.34	3.14	2.4E-03	5.2E-03	7.59
8_1	8146462	SOX17	28	0.17	0.33	8.67	2.58	1.2E-02	1.8E-02	5.81
7_1	8136662	MGAM	168	0.61	-	5.68	-	-	1.4E-02	6.11
3_9	8091941	PDCD10	33	0.64	0.66	6.54	5.49	4.1E-08	8.2E-08	23.55
3_9	8083779	SERPINI1	62	0.27	0.24	5.03	2.02	4.3E-02	4.3E-02	4.54
3_8	8091941	PDCD10	33	0.64	0.72	6.54	6.00	7.2E-08	1.5E-07	22.72
3_7	8083757	NMD3	22	0.62	0.45	8.70	3.62	5.7E-04	4.7E-03	7.73
3_7	8091764	KPNA4	43	0.55	0.62	7.08	4.63	1.7E-05	5.7E-04	10.79
3_7	8083709	SMC4	24	0.53	0.72	7.82	3.93	2.0E-04	3.0E-03	8.37
3_7	8091780	B3GALNT1	20	0.44	0.48	7.03	3.84	2.8E-04	3.0E-03	8.37
3_7	8083707	ENST00000326474	7	0.43	0.15	5.21	3.12	2.7E-03	1.5E-02	6.09
3_7	8091757	TRIM59	13	0.32	0.43	6.09	3.18	2.3E-03	1.5E-02	6.09
3_7	8091737	IFT80	63	0.32	0.38	7.53	2.84	5.9E-03	2.4E-02	5.36
3_7	8091806	hCG_16001	13	-0.22	-0.34	9.74	-2.90	5.0E-03	2.4E-02	5.40
3_5	8083690	IL12A	32	0.39	0.20	5.67	2.16	3.4E-02	4.5E-02	4.47
3_5	8091733	---	13	0.25	0.15	4.29	2.39	1.9E-02	3.8E-02	4.70
3_5	8083677	SCHIP1	381	0.22	0.21	6.50	2.45	1.6E-02	3.8E-02	4.70
3_4	8083656	MFSD1	28	0.49	0.84	8.01	4.56	2.2E-05	8.8E-05	13.47
3_4	8083677	SCHIP1	381	0.22	0.20	6.50	2.43	1.8E-02	3.6E-02	4.80
3_3	8083652	AY070437	18	0.43	0.36	6.08	3.64	5.3E-04	1.1E-03	9.88
3_3	8091723	RARRES1	42	0.31	0.53	6.40	2.17	3.3E-02	3.3E-02	4.91
3_28	8084947	FBXO45	23	0.80	0.89	6.58	8.34	4.3E-12	1.7E-10	32.49
3_28	8093230	KIAA0226	37	0.80	0.53	7.75	5.64	3.3E-07	1.7E-06	19.17
3_28	8084971	SENP5	42	0.79	0.80	7.93	6.87	2.1E-09	2.4E-08	25.32
3_28	8093156	NCBP2	24	0.78	0.79	7.82	8.00	1.8E-11	4.6E-10	31.02
3_28	8084982	ENST00000319735	18	0.76	0.47	6.72	6.55	8.1E-09	7.9E-08	23.59
3_28	8093130	RNF168	31	0.76	0.70	7.11	7.25	4.3E-10	5.6E-09	27.42
3_28	8093141	WDR53	18	0.75	0.53	6.61	8.53	1.9E-12	1.5E-10	32.65
3_28	8092905	LSG1	37	0.73	0.77	7.71	7.79	4.3E-11	8.4E-10	30.15
3_28	8084986	FYTTD1	24	0.68	0.83	7.91	6.24	2.9E-08	2.1E-07	22.21
3_28	8093191	DLG1	146	0.65	0.67	8.39	5.36	1.0E-06	4.9E-06	17.64
3_28	8093112	UBXD7	44	0.64	0.70	8.23	5.75	2.2E-07	1.4E-06	19.44
3_28	8093086	PCYT1A	37	0.63	0.57	6.99	5.71	2.5E-07	1.5E-06	19.34
3_28	8084955	PIGX	17	0.63	0.90	6.30	7.34	2.9E-10	4.5E-09	27.72
3_28	8092849	ATP13A3	41	0.61	0.75	8.54	5.65	3.3E-07	1.7E-06	19.17
3_28	8085033	LMLN	49	0.60	0.44	6.60	4.34	4.6E-05	1.6E-04	12.63
3_28	8092922	C3orf21	126	0.57	0.24	7.47	4.01	1.5E-04	4.6E-04	11.07
3_28	8084844	OPA1	81	0.57	0.72	8.11	6.25	2.8E-08	2.1E-07	22.21
3_28	8084895	MUC20	54	0.56	0.65	7.30	4.54	2.3E-05	8.5E-05	13.52
3_28	8093013	TNK2	24	0.55	0.22	7.42	3.03	3.4E-03	8.4E-03	6.90
3_28	8084963	PAK2	49	0.54	0.82	7.62	5.10	2.8E-06	1.3E-05	16.24
3_28	8085026	RPL35A	15	0.53	0.54	10.30	4.90	5.9E-06	2.6E-05	15.24
3_28	8085000	LRCH3	55	0.53	0.32	7.41	3.37	1.2E-03	3.2E-03	8.30
3_28	8093053	TFRC	31	0.51	1.03	8.59	4.83	7.8E-06	3.2E-05	14.93
3_28	8084838	HRASLS	30	0.49	0.33	6.37	3.71	4.1E-04	1.2E-03	9.74
3_28	8093145	ENST00000311566	11	0.48	0.37	5.42	4.76	1.0E-05	4.0E-05	14.61
3_28	8093219	BDH1	44	0.48	0.31	6.66	3.48	8.6E-04	2.3E-03	8.75
3_28	8092888	TMEM44	39	0.46	0.16	6.95	2.99	3.8E-03	8.8E-03	6.83
3_28	8092959	PPP1R2	17	0.46	0.33	7.45	4.14	9.6E-05	3.1E-04	11.64
3_28	8084904	MUC20	4	0.46	0.45	9.80	4.00	1.6E-04	4.7E-04	11.06
3_28	8093039	SDHA	9	0.45	0.34	9.52	3.02	3.6E-03	8.4E-03	6.89
3_28	8092765	ENST00000315470	116	0.44	0.44	7.07	4.36	4.3E-05	1.5E-04	12.68
3_28	8092933	CENTB2	70	0.40	0.74	8.17	6.26	2.6E-08	2.1E-07	22.21
3_28	8084891	FAM43A	18	0.39	0.27	7.14	3.04	3.4E-03	8.4E-03	6.90
3_28	8092957	---	4	0.37	0.25	6.28	2.73	8.1E-03	1.7E-02	5.84
3_28	8084923	FLJ25996	14	0.35	0.22	5.53	2.81	6.5E-03	1.4E-02	6.12
3_28	8084878	---	14	0.30	0.59	5.67	3.64	5.2E-04	1.5E-03	9.42
3_27	8092750	FGF12	463	0.27	0.24	5.81	2.35	2.0E-02	4.1E-02	4.63
3_25	8084742	LPP	506	0.55	0.29	8.45	3.04	3.0E-03	6.0E-03	7.39
3_25	8084739	FLJ42393	21	0.48	0.17	6.70	2.05	4.3E-02	4.3E-02	4.54
3_24	8092640	RFC4	22	0.74	0.77	7.68	6.62	6.8E-09	1.6E-07	22.54
3_24	8092654	RPL39L	26	0.73	0.71	6.64	5.55	5.1E-07	6.2E-06	17.31
3_24	8084634	DNAJB11	23	0.66	0.66	8.26	5.26	1.6E-06	1.3E-05	16.25
3_24	8092691	BCL6	29	0.48	0.37	7.47	2.44	1.7E-02	4.6E-02	4.44
3_24	8092627	TBCCD1	25	0.47	0.29	6.77	3.64	5.3E-04	2.1E-03	8.89
3_24	8084717	ST6GAL1	150	0.42	0.58	8.24	2.50	1.5E-02	4.5E-02	4.48
3_24	8084732	RTP4	18	0.41	0.40	6.55	2.38	2.0E-02	4.8E-02	4.37
3_24	8084694	EIF4A2	20	0.40	0.49	9.76	3.84	2.7E-04	1.6E-03	9.28
3_24	8084704	ENST00000363548	14	0.37	0.56	6.15	3.66	4.9E-04	2.1E-03	8.89
3_24	8084708	SNORA4	15	0.29	0.45	7.02	2.64	1.0E-02	3.5E-02	4.85
3_22	8092534	TMEM41A	14	0.77	0.82	8.05	6.97	1.4E-09	1.1E-08	26.40
3_22	8084607	SENP2	29	0.70	0.82	7.00	7.21	5.4E-10	8.6E-09	26.80
3_22	8092564	SFRS10	22	0.56	0.47	8.87	4.96	4.8E-06	2.6E-05	15.24
3_22	8084541	VPS8	137	0.44	0.47	7.38	3.97	1.7E-04	6.9E-04	10.49
3_22	8092523	EHHADH	50	0.32	0.25	6.46	2.68	9.2E-03	2.9E-02	5.08
3_20	8092409	PARL	37	0.77	0.63	8.58	7.99	1.5E-11	1.3E-10	32.89
3_20	8084439	EIF4G1	13	0.76	0.72	8.89	7.96	1.8E-11	1.3E-10	32.89
3_20	8084360	ABCF3	16	0.75	0.70	7.73	8.64	9.4E-13	2.0E-11	35.57
3_20	8084323	DVL3	12	0.74	0.45	8.19	6.24	2.6E-08	9.4E-08	23.34
3_20	8084232	YEATS2	66	0.73	0.61	7.90	5.99	7.4E-08	2.2E-07	22.10
3_20	8084303	EIF2B5	14	0.72	0.41	7.36	6.24	2.6E-08	9.4E-08	23.34
3_20	8084219	KLHL24	35	0.70	0.84	8.02	7.82	3.2E-11	1.7E-10	32.49
3_20	8084423	PSMD2	10	0.66	0.83	7.55	8.61	1.1E-12	2.0E-11	35.57
3_20	8084488	POLR2H	12	0.65	0.82	8.33	6.97	1.3E-09	5.6E-09	27.41
3_20	8092457	ALG3	8	0.62	0.64	8.37	7.86	2.7E-11	1.6E-10	32.51
3_20	8084345	AP2M1	9	0.54	0.88	9.43	8.36	3.2E-12	3.8E-11	34.62
3_20	8092514	MAGEF1	8	0.50	0.38	8.73	5.99	7.4E-08	2.2E-07	22.10
3_20	8092328	MCCC1	53	0.49	0.61	7.81	5.05	3.2E-06	8.7E-06	16.81
3_20	8092418	ABCC5	57	0.43	0.38	7.22	3.62	5.5E-04	1.3E-03	9.56
3_20	8092348	LAMP3	51	0.43	0.63	6.84	3.93	1.9E-04	4.9E-04	10.98
3_20	8084397	ECE2	26	0.36	0.15	7.04	2.54	1.3E-02	2.6E-02	5.25
3_20	8084299	HSP90AA5P	17	0.33	0.19	4.45	2.92	4.7E-03	9.9E-03	6.66
3_20	8084524	EPHB3	19	0.28	0.38	7.17	3.09	2.9E-03	6.4E-03	7.28
3_2	8083630	GFM1	32	0.62	0.56	7.19	4.64	1.6E-05	1.1E-04	13.20
3_2	8083605	RSRC1	229	0.54	0.72	7.58	4.47	3.1E-05	1.3E-04	12.89
3_2	8091658	CCNL1	15	0.53	0.45	8.65	3.92	2.1E-04	6.7E-04	10.55

3_2	8083616	MLF1	33	0.53	0.81	6.11	5.01	4.1E-06	5.4E-05	14.18
3_2	8091678	VEPH1	195	0.41	0.27	5.11	2.77	7.3E-03	1.6E-02	5.99
3_2	8083594	PTX3	17	0.39	0.37	5.31	3.23	1.9E-03	5.0E-03	7.65
3_2	8091715	LXN	15	0.25	0.41	5.37	2.43	1.8E-02	3.3E-02	4.93
3_2	8091698	SHOX2	10	0.24	0.19	6.29	2.25	2.8E-02	4.5E-02	4.48
3_19	8092321	DCUN1D1	41	0.50	0.65	6.82	4.92	8.5E-07	1.7E-06	19.16
3_19	8092328	MCCC1	53	0.49	0.54	7.81	4.13	3.7E-05	3.7E-05	14.73
3_18	8084146	FXR1	37	0.60	0.69	9.20	6.08	5.4E-08	3.8E-07	21.34
3_18	8092314	DNAJC19	10	0.55	0.41	6.67	4.95	4.9E-06	1.1E-05	16.42
3_18	8092321	DCUN1D1	41	0.50	0.62	6.82	4.95	4.9E-06	1.1E-05	16.42
3_18	8084173	ATP11B	66	0.30	0.46	7.39	3.60	5.8E-04	1.0E-03	9.95
3_17	8092265	MRPL47	19	0.56	0.81	7.44	5.49	6.5E-07	4.6E-06	17.74
3_17	8084067	ACTL6A	40	0.54	0.68	7.48	5.59	4.4E-07	4.6E-06	17.74
3_17	8084092	NDUFB5	14	0.40	0.98	8.14	4.56	2.2E-05	1.0E-04	13.26
3_17	8084128	TTC14	13	0.37	0.47	7.14	2.82	6.2E-03	1.5E-02	6.11
3_17	8092251	GNB4	37	0.30	0.50	6.51	4.07	1.2E-04	4.3E-04	11.19
3_17	8084064	LOC442098	19	0.20	0.54	6.99	3.38	1.2E-03	3.3E-03	8.23
3_15	8084035	ZNF639	12	0.41	0.62	6.16	4.81	3.2E-06	9.6E-06	16.67
3_15	8084045	MFN1	29	0.38	0.59	7.86	4.27	3.2E-05	4.8E-05	14.35
3_15	8092251	GNB4	37	0.30	0.48	6.51	3.69	2.9E-04	2.9E-04	11.74
3_14	8092201	TBL1XR1	110	0.48	0.76	9.32	5.05	3.3E-06	4.9E-05	14.32
3_14	8084016	PIK3CA	217	0.47	0.65	7.66	4.66	1.5E-05	1.1E-04	13.15
3_14	8092220	---	8	0.23	0.17	4.43	2.55	1.3E-02	4.8E-02	4.37
3_14	8084014	---	9	0.19	0.19	4.16	2.67	9.4E-03	4.7E-02	4.41
3_12	8083941	ECT2	39	0.59	0.80	7.35	3.75	3.7E-04	4.4E-03	7.82
3_12	8092177	AADACL1	82	0.53	0.62	7.32	3.91	2.2E-04	4.4E-03	7.82
3_10	8083794	MYNN	21	0.74	0.58	6.94	5.52	5.6E-07	1.5E-05	16.02
3_10	8092035	PHC3	45	0.73	0.68	8.28	5.38	1.0E-06	1.5E-05	16.02
3_10	8092002	ARPM1	14	0.62	0.28	5.79	4.40	3.9E-05	2.3E-04	12.07
3_10	8091991	MDS1	400	0.60	0.50	7.36	4.42	3.6E-05	2.3E-04	12.07
3_10	8083850	ENST00000359416	17	0.55	0.27	5.07	3.21	2.0E-03	6.7E-03	7.22
3_10	8083854	PRKCI	41	0.54	0.76	8.93	3.55	7.0E-04	2.6E-03	8.58
3_10	8083826	TLOC1	38	0.51	0.47	7.54	3.68	4.7E-04	2.0E-03	8.96
3_10	8083808	ENST00000340806	19	0.46	0.22	5.15	3.83	2.8E-04	1.4E-03	9.46
3_10	8083876	SKIL	25	0.45	0.87	7.49	4.82	8.3E-06	8.3E-05	13.56
3_10	8091972	EVI1	63	0.38	0.70	8.60	2.89	5.1E-03	1.5E-02	6.02
3_10	8092055	SLC7A14	107	0.28	0.23	5.98	2.47	1.6E-02	4.0E-02	4.65
3_10	8092073	EIF5A2	15	0.28	0.40	5.92	2.67	9.5E-03	2.6E-02	5.27
3_1	8091648	SSR3	27	0.44	0.73	8.05	5.00	4.1E-06	2.1E-05	15.56
3_1	8083569	TIPARP	22	0.43	0.50	7.28	3.92	2.1E-04	5.1E-04	10.92
3_1	8083546	KCNAB1	312	0.31	0.18	5.50	2.45	1.7E-02	2.8E-02	5.15
20_9	8066347	PTPRT	1076	0.58	0.48	6.23	3.23	1.9E-03	1.9E-03	9.01
20_9	8062685	---	13	0.47	0.26	5.78	3.40	1.1E-03	1.9E-03	9.01
20_8	8066347	PTPRT	1076	0.58	-	6.23	-	-	4.0E-03	7.98
20_7	8066303	CHD6	129	0.70	0.52	8.14	4.41	3.4E-05	2.9E-04	11.77
20_7	8062623	PLCG1	28	0.69	0.68	8.20	5.37	8.6E-07	1.5E-05	16.05
20_7	8062658	LPIN3	14	0.61	0.42	7.01	4.17	8.2E-05	4.6E-04	11.07
20_7	8062601	LOC149692	10	0.59	0.38	5.83	3.44	9.5E-04	2.3E-03	8.76
20_7	8066347	PTPRT	1076	0.58	0.50	6.23	3.55	6.7E-04	2.1E-03	8.93
20_7	8066275	AF090938	13	0.57	0.36	4.89	3.53	7.2E-04	2.1E-03	8.93
20_7	8062603	TOP1	50	0.55	0.42	8.60	3.87	2.3E-04	9.7E-04	10.02
20_7	8066279	ZHX3	72	0.46	0.25	6.50	2.94	4.3E-03	9.2E-03	6.77
20_7	8066273	---	17	0.45	0.26	5.49	2.57	1.2E-02	2.1E-02	5.58
20_7	8066294	EMILIN3	9	0.34	0.20	6.61	2.58	1.2E-02	2.1E-02	5.58
20_7	8066266	MAFB	11	0.24	0.24	7.07	2.23	2.9E-02	4.5E-02	4.48
20_6	8062576	DHX35	59	0.84	0.61	6.78	6.53	9.5E-09	9.3E-08	23.36
20_6	8066200	KIAA0406	33	0.76	0.61	7.45	6.75	3.7E-09	5.5E-08	24.12
20_6	8062213	PHF20	99	0.74	0.36	7.19	3.18	2.2E-03	4.5E-03	7.80
20_6	8062409	CTNNBL1	149	0.74	0.45	7.33	7.48	1.8E-10	1.0E-08	26.52
20_6	8062433	C20orf77	38	0.72	0.49	8.32	6.44	1.3E-08	1.1E-07	23.08
20_6	8062286	ENST00000373932	18	0.71	0.43	7.33	6.55	8.4E-09	9.3E-08	23.36
20_6	8062545	ACTR5	35	0.68	0.39	7.33	6.20	3.6E-08	2.6E-07	21.86
20_6	8062319	TGIF2	11	0.67	0.39	7.71	4.13	1.0E-04	3.0E-04	11.72
20_6	8066254	LOC388796	14	0.66	0.53	7.73	7.28	4.0E-10	1.2E-08	26.32
20_6	8066091	C20orf117	44	0.66	0.38	7.06	4.47	3.0E-05	1.1E-04	13.13
20_6	8066074	DSN1	15	0.64	0.60	6.89	4.97	4.7E-06	2.5E-05	15.27
20_6	8062371	MANBAL	27	0.64	0.46	7.25	6.07	6.2E-08	4.1E-07	21.23
20_6	8066247	LOC388796	18	0.62	0.52	7.42	6.86	2.4E-09	4.6E-08	24.37
20_6	8062571	FAM83D	20	0.60	0.48	7.28	5.61	3.8E-07	2.3E-06	18.76
20_6	8066136	RBL1	172	0.59	0.66	6.92	4.84	7.6E-06	3.7E-05	14.71
20_6	8062492	KIAA1219	70	0.58	0.45	8.46	4.06	1.3E-04	3.3E-04	11.54
20_6	8066031	SCAND1	11	0.56	0.27	6.90	4.53	2.4E-05	9.4E-05	13.38
20_6	8062293	DLGAP4	111	0.56	0.24	7.89	3.49	8.5E-04	1.9E-03	9.01
20_6	8066051	NDRG3	42	0.54	0.52	6.74	4.20	7.7E-05	2.4E-04	12.02
20_6	8066195	BLCAP	15	0.54	0.28	8.97	4.05	1.3E-04	3.3E-04	11.54
20_6	8066161	C20orf132	46	0.51	0.29	5.79	4.09	1.1E-04	3.2E-04	11.61
20_6	8062480	C20orf198	12	0.51	0.28	7.23	4.73	1.2E-05	5.2E-05	14.23
20_6	8062349	RPN2	57	0.47	0.68	8.94	4.66	1.5E-05	6.2E-05	13.98
20_6	8062444	BPI	43	0.44	0.33	5.80	4.38	4.2E-05	1.4E-04	12.84
20_6	8066072	---	5	0.44	0.23	4.55	3.25	1.8E-03	3.9E-03	8.02
20_6	8066210	---	15	0.42	0.37	3.74	2.63	1.0E-02	1.9E-02	5.74
20_6	8062326	C20orf24	8	0.40	0.46	7.79	4.41	3.7E-05	1.3E-04	12.93
20_6	8062395	NNAT	11	0.39	0.33	6.46	3.94	1.9E-04	4.7E-04	11.07
20_6	8066038	SLA2	22	0.38	0.24	6.15	3.05	3.3E-03	6.2E-03	7.33
20_6	8062251	EPB41L1	70	0.36	0.32	7.17	2.96	4.2E-03	7.7E-03	7.01
20_6	8062555	ENST00000362821	15	0.35	0.20	5.19	2.55	1.3E-02	2.2E-02	5.52
20_6	8062284	---	13	0.33	0.30	7.87	2.19	3.2E-02	5.0E-02	4.33
20_6	8062237	C20orf152	39	0.29	0.30	5.95	3.69	4.4E-04	1.0E-03	9.90
20_6	8062377	SRC	38	0.29	0.24	7.14	3.07	3.0E-03	6.0E-03	7.39
20_6	8062404	ENST00000373508	43	0.25	0.21	6.14	2.61	1.1E-02	1.9E-02	5.69
20_6	8066212	---	5	0.25	0.30	7.12	2.53	1.4E-02	2.2E-02	5.48
20_6	8062347	RBL1	45	0.23	0.23	6.82	2.35	2.2E-02	3.5E-02	4.85
20_6	8066260	SNORA71C	13	0.18	0.47	8.71	3.19	2.2E-03	4.5E-03	7.80
20_5	8065963	RBM12	23	0.75	0.57	8.58	5.24	1.3E-06	6.4E-06	17.26
20_5	8065992	NFS1	18	0.62	0.31	7.58	3.94	1.8E-04	2.9E-04	11.73
20_5	8066009	RBM39	31	0.62	0.48	9.91	3.98	1.5E-04	2.9E-04	11.73
20_5	8065990	---	13	0.50	0.42	4.62	3.56	6.3E-04	6.3E-04	10.64
20_5	8062206	C20orf52	8	0.50	0.34	7.62	3.58	5.9E-04	6.3E-04	10.64
20_4	8065963	RBM12	23	0.75	0.62	8.58	6.04	5.5E-08	1.0E-06	19.93
20_4	8062137	CEP250	52	0.74	0.45	6.97	5.47	5.6E-07	3.0E-06	18.34
20_4	8065776	NCOA6	62	0.74	0.49	8.03	5.63	3.0E-07	2.3E-06	18.75
20_4	8065889	UQCC	66	0.70	0.51	7.52	5.04	3.2E-06	1.1E-05	16.45
20_4	8062016	DYNLRB1	18	0.70	0.49	7.96	5.93	8.7E-08	1.0E-06	19.93
20_4	8065798	GGTL3	17	0.64	0.51	7.00	5.96	7.4E-08	1.0E-06	19.93
20_4	8061966	RALY	43	0.64	0.48	8.94	5.65	2.7E-07	2.3E-06	18.75
20_4	8065730	EIF2S2	15	0.62	0.47	7.79	4.88	5.7E-06	1.6E-05	15.90

20_4	8065832	TRPC4AP	57	0.62	0.53	8.35	5.48	5.4E-07	3.0E-06	18.34
20_4	8065762	PIGU	48	0.61	0.84	7.40	7.03	7.9E-10	3.6E-08	24.72
20_4	8065693	APBA2BP	10	0.59	0.40	7.41	5.46	5.9E-07	3.0E-06	18.34
20_4	8065719	PXMP4	12	0.58	0.44	6.65	4.97	4.0E-06	1.3E-05	16.21
20_4	8065903	---	11	0.58	0.51	6.21	4.92	4.9E-06	1.5E-05	16.02
20_4	8065868	EIF6	13	0.56	0.49	8.22	5.38	8.2E-07	3.8E-06	18.01
20_4	8061919	CBFA2T2	61	0.55	0.36	6.67	3.75	3.5E-04	7.3E-04	10.41
20_4	8061986	ITCH	63	0.54	0.38	8.41	3.82	2.7E-04	5.9E-04	10.73
20_4	8061958	CHMP4B	28	0.53	0.23	8.74	3.56	6.6E-04	1.3E-03	9.58
20_4	8062041	ACSS2	30	0.53	0.36	7.33	4.47	2.8E-05	7.0E-05	13.80
20_4	8065710	E2F1	6	0.51	0.32	7.25	4.44	3.1E-05	7.5E-05	13.71
20_4	8065855	EDEM2	42	0.49	0.49	6.81	5.21	1.6E-06	6.8E-06	17.17
20_4	8065738	AHCY	15	0.49	0.46	8.81	4.25	6.1E-05	1.4E-04	12.79
20_4	8062174	ERGIC3	9	0.48	0.63	9.70	4.50	2.4E-05	6.5E-05	13.91
20_4	8062123	MMP24	31	0.47	0.24	6.28	3.20	2.0E-03	3.8E-03	8.06
20_4	8065817	GSS	22	0.45	0.43	7.98	5.06	2.9E-06	1.1E-05	16.46
20_4	8065911	C20orf173	13	0.43	0.17	6.07	2.16	3.4E-02	4.7E-02	4.40
20_4	8065758	FLJ38773	7	0.39	0.35	7.28	3.37	1.2E-03	2.3E-03	8.79
20_4	8061982	ASIP	13	0.35	0.17	6.37	2.23	2.9E-02	4.1E-02	4.61
20_4	8062134	GDF5OS	9	0.34	0.22	6.03	2.58	1.2E-02	2.0E-02	5.62
20_4	8062023	MAP1LC3A	9	0.34	0.19	6.59	2.44	1.7E-02	2.7E-02	5.21
20_4	8065905	GDF5	18	0.32	0.19	5.82	2.59	1.2E-02	2.0E-02	5.62
20_4	8065756	GENSCAN00000000672	2	0.31	0.25	5.91	2.55	1.3E-02	2.1E-02	5.59
20_4	8065880	FAM83C	13	0.30	0.23	7.29	2.26	2.7E-02	4.0E-02	4.64
20_4	8062064	MYH7B	28	0.28	0.20	6.42	2.31	2.4E-02	3.6E-02	4.78
20_4	8062108	PROCR	19	0.20	0.13	6.39	2.13	3.6E-02	4.9E-02	4.35
20_20	8063814	LSM14B	21	0.76	0.52	7.15	7.40	2.0E-10	1.7E-08	25.80
20_20	8063857	GTPBP5	23	0.72	0.33	7.59	5.62	3.5E-07	4.9E-06	17.65
20_20	8063839	SS18L1	36	0.71	0.38	6.99	5.78	1.8E-07	3.9E-06	17.99
20_20	8067361	TAF4	62	0.70	0.35	7.86	5.27	1.4E-06	1.4E-05	16.10
20_20	8064218	PRPF6	32	0.69	0.43	8.24	3.71	4.1E-04	1.6E-03	9.30
20_20	8064302	PCMTD2	29	0.67	0.53	8.72	4.15	9.1E-05	5.4E-04	10.85
20_20	8063835	LSM14B	13	0.66	0.63	6.53	7.22	4.5E-10	1.9E-08	25.65
20_20	8067563	DIDO1	49	0.64	0.40	8.05	5.70	2.5E-07	4.2E-06	17.87
20_20	8067727	ARFRP1	10	0.62	0.23	7.55	3.68	4.5E-04	1.6E-03	9.25
20_20	8064208	DNAJC5	25	0.62	0.22	8.28	4.01	1.5E-04	8.2E-04	10.25
20_20	8067593	YTHDF1	19	0.61	0.52	8.46	6.45	1.1E-08	3.2E-07	21.59
20_20	8067773	ZNF512B	39	0.60	0.31	7.39	3.61	5.6E-04	1.9E-03	9.02
20_20	8064191	TPD52L2	14	0.59	0.43	8.10	3.89	2.2E-04	1.1E-03	9.77
20_20	8067495	CABLES2	14	0.57	0.29	6.97	3.88	2.3E-04	1.1E-03	9.77
20_20	8067382	PSMA7	18	0.57	0.44	8.31	5.43	7.3E-07	8.7E-06	16.81
20_20	8067756	UCKL1	8	0.56	0.36	7.28	3.78	3.3E-04	1.4E-03	9.51
20_20	8063873	OSBPL2	44	0.55	0.38	7.94	3.70	4.2E-04	1.6E-03	9.30
20_20	8063755	C20orf177	20	0.54	0.49	5.75	4.59	1.8E-05	1.3E-04	12.92
20_20	8067554	TCFL5	16	0.50	0.32	7.55	4.69	1.3E-05	9.9E-05	13.31
20_20	8064042	ARFGAP1	10	0.50	0.23	7.82	3.82	2.8E-04	1.2E-03	9.67
20_20	8063793	LOC284757	35	0.50	0.33	6.01	2.81	6.4E-03	1.5E-02	6.03
20_20	8064007	C20orf11	10	0.50	0.65	8.39	5.23	1.6E-06	1.5E-05	16.02
20_20	8067709	GMEB2	35	0.49	0.19	7.78	3.83	2.7E-04	1.2E-03	9.67
20_20	8064245	TCEA2	13	0.48	0.17	7.46	2.59	1.2E-02	2.6E-02	5.25
20_20	8067380	LSM14B	13	0.46	0.21	5.32	3.18	2.2E-03	5.9E-03	7.41
20_20	8063949	C20orf20	11	0.44	0.27	7.29	4.88	6.3E-06	5.3E-05	14.21
20_20	8063903	RPS21	11	0.44	0.37	8.57	3.50	8.1E-04	2.5E-03	8.63
20_20	8064277	MYT1	80	0.43	0.31	6.39	4.15	8.9E-05	5.4E-04	10.85
20_20	8063893	ADRM1	13	0.43	0.34	8.20	3.60	5.7E-04	1.9E-03	9.02
20_20	8067305	SYCP2	40	0.41	0.66	4.75	3.57	6.3E-04	2.0E-03	8.93
20_20	8067409	LAMA5	25	0.41	0.48	8.63	3.34	1.3E-03	4.0E-03	7.96
20_20	8063921	hsa-mir-1-1	9	0.40	0.27	3.97	3.25	1.8E-03	4.9E-03	7.67
20_20	8064111	TNFRSF6B	40	0.37	0.17	7.01	3.11	2.7E-03	6.9E-03	7.19
20_20	8063761	CDH26	73	0.36	0.25	5.96	3.17	2.2E-03	5.9E-03	7.41
20_20	8063785	GENSCAN00000041041	40	0.36	0.23	6.34	2.64	1.0E-02	2.3E-02	5.41
20_20	8067358	ENST00000317652	20	0.36	0.21	5.60	2.84	5.9E-03	1.5E-02	6.11
20_20	8064175	SLC2A4RG	11	0.28	0.26	7.32	3.28	1.6E-03	4.6E-03	7.76
20_20	8067680	PRIC285	10	0.28	0.19	7.25	2.50	1.5E-02	3.3E-02	4.93
20_2	8061919	CBFA2T2	61	0.55	0.41	6.67	4.32	5.1E-05	1.0E-04	13.25
20_2	8065683	SNTA1	14	0.41	0.18	7.44	3.13	2.5E-03	2.5E-03	8.63
20_18	8063620	VAPB	65	0.65	0.48	7.70	5.44	7.6E-07	9.1E-06	16.74
20_18	8063607	RAB22A	47	0.61	0.45	8.29	5.20	2.0E-06	1.4E-05	16.16
20_18	8063697	TH1L	16	0.59	0.57	8.40	4.78	9.4E-06	5.0E-05	14.30
20_18	8063716	TUBB1	12	0.58	0.33	5.93	5.40	8.7E-07	9.1E-06	16.74
20_18	8063668	GNAS	51	0.56	0.29	8.92	3.51	8.0E-04	2.1E-03	8.90
20_18	8063650	NPEPL1	22	0.55	0.18	7.35	2.60	1.1E-02	2.2E-02	5.51
20_18	8067248	PPP4R1L	88	0.54	0.31	6.43	3.31	1.5E-03	3.1E-03	8.33
20_18	8067295	SLMO2	15	0.52	0.61	8.48	4.48	2.9E-05	1.2E-04	13.03
20_18	8063723	ENST00000371030	68	0.42	0.20	6.18	2.42	1.8E-02	3.1E-02	4.99
20_18	8067305	SYCP2	40	0.41	0.64	4.75	3.40	1.1E-03	2.6E-03	8.58
20_18	8063636	STX16	32	0.36	0.46	8.19	3.84	2.7E-04	9.5E-04	10.04
20_18	8067288	ATP5E	9	0.35	0.41	7.72	3.65	5.0E-04	1.5E-03	9.38
20_16	8063509	C20orf43	41	0.71	0.48	7.77	7.11	9.7E-10	1.4E-08	26.10
20_16	8063566	RAE1	28	0.71	0.72	7.57	7.08	1.1E-09	1.4E-08	26.10
20_16	8063522	---	10	0.61	0.26	4.14	3.94	1.9E-04	1.2E-03	9.69
20_16	8067201	ENST00000388144	15	0.56	0.64	6.03	4.13	1.0E-04	8.6E-04	10.18
20_16	8067199	ENST00000388140	15	0.54	0.30	4.78	2.93	4.6E-03	1.9E-02	5.70
20_16	8063583	RBM38	21	0.48	0.29	7.43	2.72	8.4E-03	3.0E-02	5.06
20_16	8067178	GCNT7	30	0.45	0.21	6.23	2.61	1.1E-02	3.5E-02	4.84
20_16	8067185	BMP7	88	0.37	0.66	6.70	3.71	4.3E-04	2.1E-03	8.86
20_15	8063484	CSTF1	28	0.77	0.60	7.04	8.03	1.8E-11	3.5E-11	34.72
20_15	8067167	AURKA	45	0.54	0.72	6.52	5.55	4.9E-07	4.9E-07	20.97
20_14	8067094	ZFP64	79	0.74	0.43	6.75	5.34	1.1E-06	2.7E-05	15.18
20_14	8063453	PFDN4	29	0.62	0.56	5.60	4.95	5.1E-06	4.1E-05	14.59
20_14	8067155	ENST00000388080	24	0.60	0.25	5.26	3.13	2.6E-03	1.0E-02	6.59
20_14	8067055	ATP9A	145	0.60	0.53	8.21	3.83	2.8E-04	1.7E-03	9.24
20_14	8063433	---	21	0.58	0.25	4.90	2.63	1.1E-02	3.2E-02	4.98
20_14	8067121	SUMO1P1	19	0.55	0.21	6.03	2.38	2.0E-02	4.8E-02	4.39
20_14	8067167	AURKA	45	0.54	0.68	6.51	5.15	2.3E-06	2.8E-05	15.12
20_14	8063476	MC3R	35	0.49	0.35	6.64	3.17	2.3E-03	1.0E-02	6.59
20_14	8063444	TSHZ2	17	0.48	0.28	6.09	2.42	1.8E-02	4.8E-02	4.39
20_14	8063447	---	18	0.39	0.48	4.89	3.06	3.1E-03	1.1E-02	6.54
20_12	8066953	SPATA2	20	0.73	0.35	6.71	5.47	6.6E-07	2.8E-06	18.45
20_12	8063427	MOCS3	14	0.71	0.46	6.17	6.41	1.5E-08	2.1E-07	22.18
20_12	8066964	TMEM189	47	0.71	0.49	7.31	7.22	5.0E-10	1.9E-08	25.66
20_12	8063315	DDX27	27	0.71	0.59	7.49	6.22	3.2E-08	2.4E-07	21.97
20_12	8063351	SLC9A8	63	0.70	0.43	7.36	4.60	1.8E-05	5.8E-05	14.07
20_12	8066889	STAU1	47	0.65	0.57	8.43	6.28	2.5E-08	2.3E-07	22.03
20_12	8067011	ADNP	44	0.62	0.64	8.59	5.70	2.6E-07	1.3E-06	19.61
20_12	8063283	CSE1L	40	0.62	0.96	8.23	6.09	5.3E-08	3.4E-07	21.50

20_12	8063242	ARFGEF2	84	0.60	0.59	8.09	5.92	1.1E-07	5.9E-07	20.70
20_12	8063394	PTPN1	174	0.57	0.35	7.97	4.77	9.9E-06	3.4E-05	14.84
20_12	8063345	ENST00000386307	11	0.52	0.64	5.24	5.13	2.5E-06	9.5E-06	16.69
20_12	8067017	DPM1	22	0.50	0.83	7.93	6.38	1.7E-08	2.1E-07	22.18
20_12	8063410	PARD6B	16	0.50	0.45	8.97	3.47	9.0E-04	2.4E-03	8.68
20_12	8066905	ZNFX1	34	0.47	0.42	7.68	2.95	4.3E-03	9.5E-03	6.71
20_12	8063369	ZNF313	16	0.44	0.39	7.96	3.34	1.3E-03	3.4E-03	8.21
20_12	8066921	KCNB1	88	0.44	0.21	6.16	2.35	2.2E-02	3.7E-02	4.75
20_12	8066848	PREX1	134	0.41	0.31	7.08	2.77	7.2E-03	1.4E-02	6.11
20_12	8063349	---	13	0.40	0.18	4.45	2.33	2.3E-02	3.7E-02	4.75
20_12	8067029	KCNG1	13	0.38	0.41	6.82	3.29	1.6E-03	3.7E-03	8.06
20_12	8063337	C20orf199	20	0.37	0.51	8.28	3.77	3.4E-04	1.0E-03	9.97
20_12	8066960	---	14	0.37	0.27	5.45	2.87	5.4E-03	1.1E-02	6.44
20_12	8067033	KCNG1	15	0.34	0.19	6.95	2.45	1.7E-02	3.2E-02	4.98
20_12	8063389	LOC284751	31	0.33	0.25	6.92	2.34	2.2E-02	3.7E-02	4.75
20_10	8062981	PIGT	25	0.74	0.65	7.85	7.77	5.8E-11	4.2E-09	27.81
20_10	8066697	SLC35C2	19	0.68	0.42	7.70	4.65	1.6E-05	1.6E-04	12.57
20_10	8063028	DNTTIP1	22	0.68	0.38	7.56	5.09	3.0E-06	4.4E-05	14.46
20_10	8063043	UBE2C	13	0.68	0.75	7.64	6.71	4.6E-09	1.7E-07	22.50
20_10	8066461	TOMM34	33	0.66	0.58	7.59	5.18	2.2E-06	4.0E-05	14.61
20_10	8063074	ZSWIM1	15	0.66	0.28	6.82	5.40	9.4E-07	2.3E-05	15.42
20_10	8066668	NCOA5	32	0.65	0.36	8.06	4.91	6.0E-06	7.3E-05	13.73
20_10	8063097	C20orf67	14	0.65	0.32	7.22	3.79	3.3E-04	2.0E-03	8.97
20_10	8066598	ACOT8	19	0.64	0.25	7.11	4.10	1.1E-04	1.0E-03	9.95
20_10	8062890	C20orf119	49	0.60	0.35	7.52	3.37	1.2E-03	4.3E-03	7.85
20_10	8066612	NEURL2	12	0.58	0.25	7.00	4.07	1.2E-04	1.0E-03	9.95
20_10	8066641	ZNF335	28	0.57	0.22	7.25	3.54	7.4E-04	3.0E-03	8.39
20_10	8066786	ZMYND8	74	0.56	0.52	7.94	3.72	4.0E-04	2.0E-03	8.97
20_10	8063057	SNX21	18	0.56	0.20	7.02	3.72	4.1E-04	2.0E-03	8.97
20_10	8063209	ENST00000360965	12	0.56	0.30	6.02	3.38	1.2E-03	4.3E-03	7.85
20_10	8066745	ZNF334	22	0.53	0.46	6.39	2.25	2.8E-02	4.6E-02	4.43
20_10	8066528	ENST00000300192	19	0.52	0.40	4.87	3.88	2.4E-04	1.6E-03	9.26
20_10	8066716	ELMO2	27	0.51	0.39	7.38	3.72	4.1E-04	2.0E-03	8.97
20_10	8066739	ZNF663	39	0.51	0.24	4.93	2.58	1.2E-02	2.6E-02	5.24
20_10	8062964	SYS1	21	0.51	0.24	7.30	3.26	1.7E-03	5.3E-03	7.57
20_10	8066609	C20orf165	14	0.51	0.23	6.58	3.29	1.6E-03	5.1E-03	7.62
20_10	8062971	DBNDD2	13	0.47	0.18	7.57	2.60	1.1E-02	2.6E-02	5.27
20_10	8066521	C20orf10	18	0.46	0.20	6.01	2.65	1.0E-02	2.4E-02	5.37
20_10	8062908	STK4	74	0.46	0.36	8.47	3.13	2.6E-03	6.8E-03	7.20
20_10	8062880	YWHAB	21	0.45	0.41	8.92	3.33	1.4E-03	4.7E-03	7.74
20_10	8066776	TP53RK	19	0.44	0.22	6.51	3.66	5.0E-04	2.3E-03	8.79
20_10	8063071	ZSWIM3	22	0.43	0.15	5.83	2.34	2.2E-02	4.1E-02	4.63
20_10	8063187	EYA2	368	0.43	0.52	7.73	2.32	2.3E-02	4.1E-02	4.61
20_10	8066542	SPINLW1	34	0.42	0.40	5.71	3.14	2.5E-03	6.8E-03	7.21
20_10	8062933	SEMG1	9	0.41	0.33	5.54	3.14	2.5E-03	6.8E-03	7.21
20_10	8066569	WFDC11	22	0.41	0.20	4.82	2.38	2.0E-02	3.9E-02	4.70
20_10	8063211	NCOA3	101	0.39	0.50	8.28	3.49	8.4E-04	3.2E-03	8.28
20_10	8063011	WFDC10A	15	0.39	0.33	5.82	3.21	2.0E-03	5.9E-03	7.40
20_10	8066530	AY372172	19	0.39	0.16	4.97	2.52	1.4E-02	3.0E-02	5.04
20_10	8063019	SPINT4	15	0.39	0.22	4.71	2.73	8.1E-03	2.0E-02	5.61
20_10	8066475	KCNS1	13	0.38	0.27	5.94	3.87	2.5E-04	1.6E-03	9.26
20_10	8066637	FLJ40606	5	0.38	0.16	6.81	2.37	2.1E-02	3.9E-02	4.70
20_10	8066549	WFDC8	53	0.36	0.32	5.40	2.64	1.0E-02	2.4E-02	5.37
20_10	8063078	CTSA	18	0.36	0.49	8.89	3.59	6.2E-04	2.7E-03	8.55
20_10	8063129	SLC12A5	21	0.35	0.19	5.88	2.37	2.0E-02	3.9E-02	4.70
20_10	8066536	WFDC6	26	0.35	0.24	6.24	2.41	1.9E-02	3.8E-02	4.71
20_10	8066489	WFDC12	11	0.33	0.23	6.72	2.42	1.8E-02	3.8E-02	4.72
20_10	8066574	WFDC10B	28	0.28	0.18	6.05	2.32	2.4E-02	4.1E-02	4.61
20_10	8066579	WFDC3	25	0.27	0.18	6.48	2.28	2.6E-02	4.3E-02	4.53
20_10	8066498	MATN4	25	0.22	0.17	6.67	2.22	3.0E-02	4.8E-02	4.38
20_1	8065668	CDK5RAP1	30	0.60	0.34	7.24	4.72	1.1E-05	8.6E-05	13.51
20_1	8061725	ASXL1	52	0.60	0.48	7.93	5.50	5.2E-07	7.1E-06	17.10
20_1	8065596	PDRG1	12	0.53	0.39	6.20	5.95	8.4E-08	3.5E-06	18.11
20_1	8061579	TPX2	37	0.53	1.04	7.51	5.15	2.1E-06	1.9E-05	15.70
20_1	8065607	PLAGL2	19	0.51	0.66	7.34	5.63	3.1E-07	5.6E-06	17.44
20_1	8065637	COMMD7	29	0.50	0.38	7.79	4.17	8.2E-05	5.0E-04	10.97
20_1	8061706	POFUT1	28	0.49	0.55	7.70	5.84	1.3E-07	3.5E-06	18.11
20_1	8061772	MAPRE1	35	0.48	0.50	9.23	4.39	3.7E-05	2.6E-04	11.93
20_1	8061517	ENST00000390832	8	0.47	0.19	3.48	2.69	8.9E-03	1.9E-02	5.74
20_1	8065612	C20orf112	66	0.45	0.33	7.08	3.57	6.4E-04	3.2E-03	8.30
20_1	8065555	DEFB119	16	0.43	0.21	5.67	2.43	1.7E-02	3.1E-02	5.02
20_1	8061685	TM9SF4	32	0.42	0.59	8.40	5.43	7.0E-07	7.6E-06	17.00
20_1	8065683	SNTA1	14	0.41	0.17	7.44	2.80	6.6E-03	1.5E-02	6.05
20_1	8061509	DEFB118	8	0.40	0.27	5.71	3.19	2.1E-03	7.6E-03	7.04
20_1	8061847	C20orf70	31	0.38	0.22	5.92	2.61	1.1E-02	2.2E-02	5.49
20_1	8061645	---	6	0.37	0.32	6.21	3.24	1.8E-03	7.0E-03	7.15
20_1	8065633	ENST00000360170	6	0.36	0.19	6.92	2.59	1.2E-02	2.3E-02	5.45
20_1	8061504	DEFB115	15	0.36	0.26	5.88	3.07	3.0E-03	8.7E-03	6.84
20_1	8061620	TTLL9	37	0.34	0.27	6.85	2.85	5.7E-03	1.4E-02	6.20
20_1	8061666	---	7	0.33	0.24	5.88	3.11	2.7E-03	8.6E-03	6.86
20_1	8065580	DUSP15	20	0.33	0.19	6.75	3.15	2.3E-03	8.0E-03	6.97
20_1	8061859	RP11-49G10.8	27	0.32	0.25	5.44	3.30	1.5E-03	6.3E-03	7.30
20_1	8061912	ENST00000246222	24	0.30	0.31	6.44	3.53	7.1E-04	3.2E-03	8.27
20_1	8065652	SPAG4L	26	0.28	0.21	5.90	2.95	4.3E-03	1.2E-02	6.42
20_1	8061869	C20orf71	29	0.28	0.23	6.42	2.38	2.0E-02	3.4E-02	4.88
20_1	8061815	C20orf185	34	0.28	0.22	6.87	2.48	1.5E-02	2.9E-02	5.10
20_1	8061746	DNMT3B	50	0.28	0.30	6.69	3.09	2.8E-03	8.6E-03	6.86
20_1	8061799	BPIL3	41	0.26	0.19	6.40	2.29	2.5E-02	4.1E-02	4.60
20_1	8061605	MYLK2	19	0.24	0.21	6.28	2.76	7.2E-03	1.6E-02	5.98
20_1	8061715	KIF3B	36	0.24	0.32	7.50	4.12	9.7E-05	5.3E-04	10.87
20_1	8061916	ENST00000253375	17	0.23	0.16	4.74	2.47	1.6E-02	2.9E-02	5.10
20_1	8065630	FLJ33706	12	0.22	0.29	5.67	2.87	5.3E-03	1.4E-02	6.20
20_1	8061542	HM13	38	0.21	0.34	8.36	2.86	5.5E-03	1.4E-02	6.20
20_1	8061883	PLUNC	32	0.20	0.19	6.36	2.20	3.1E-02	5.0E-02	4.33
