# Supplementary material for: Identification of Candidate Growth Promoting Genes in Ovarian Cancer through Integrated Copy Number and Expression Analysis
Source: PLoS One. 2010 Apr 8;5(4):e9983. doi: 10.1371/journal.pone.0009983 (PMC2851616; doi:10.1371/journal.pone.0009983)
Supplement: Table S6 — Correlation for all genes highly amplified (CN>5) in at least 5 samples. This table displays Pearson's correlation between copy number and gene expression for all 181 probesets in regions of high CN gain across the genome. The p-value displayed is a raw p-value obtained while testing for correlation. * Genes highly amplified in 4 samples but that were within 10 kb of a copy number breakpoint of 5 amplified samples. (0.03 MB PDF) [file pone.0009983.s006.pdf]

Probeset	P.value	Pearson correlation (r)	Chr	Start (bp)	End (bp)	Frequency of high level amplification	Number of CN probesets	Gene
8152648	2.22E-16	0.8003	chr8	124301388	124322798	5	28	BC012379 // C8orf76 // chromosome 8 open reading frame 76 // 8q24.13 // 84933
8027566	3.49E-14	0.7640	chr19	38556076	38565432	5	32	NM_001806 // CEBPG // CCAAT/enhancer binding protein (C/EBP)
8027439	4.77E-14	0.7614	chr19	37528393	37570411	5	33	NM_014910 // ZNF507 // zinc finger protein 507 // 19q13.11 // 22847 /// ENST00000311921 // ZNF507 // zinc finger protein 507 (ZNF507)
8035905	5.42E-14	0.7604	chr19	37779747	37857945	5	34	NM_032139 // ANKRD27 // ankyrin repeat domain 27 (VPS9 domain) // 19q13.11 // 84079 /// ENST00000306065 // ANKRD27 // ankyrin repeat domain 27 (VPS9 domain) (ANKRD27)
8035886	7.53E-14	0.7576	chr19	34881633	34898292	6	29	NM_031448 // C19orf12 // chromosome 19 open reading frame 12 // 19q12 // 83636 /// NM_001031726 // C19orf12 // chromosome 19 open reading frame 12 // 19q12 // 83636
8146618	1.41E-13	0.7523	chr8	62363104	62576758	5	29	ENST00000325897 // RLBP1L1 // hypothetical protein MGC34646 (MGC34646)
8006906	1.44E-12	0.7312	chr17	35097919	35138441	5	43	NM_001005862 // ERBB2 // v-erb-b2 erythroblastic leukemia viral oncogene homolog 2
8152815	1.82E-12	0.7289	chr8	129027987	129030151	7	25	ENST00000328323 // TMEM75 // transmembrane protein 75 (TMEM75)
8148313	2.04E-12	0.7279	chr8	127752949	127753081	6	25	ENST00000391149 // ENSEMBL ncRNA // ncrna:snRNA chromosome:NCBI36:8:127752949:127753081:1 gene:ENSG00000212451 // chr8 // 100 // 100 // 25 // 25 // 0
8036010	2.56E-12	0.7257	chr19	38569696	38704540	7	27	NM_000285 // PEPD // peptidase D // 19q12-q13.2 // 5184 /// ENST00000244137 // PEPD // peptidase D (PEPD)
8027390	4.46E-12	0.7202	chr19	34789041	34798547	6	32	NM_006627 // POP4 // processing of precursor 4
8148327	9.67E-12	0.7123	chr8	129301932	129302259	7	26	ENST00000364912 // ENSEMBL ncRNA // ncrna:misc_RNA chromosome:NCBI36:8:129301932:129302231:1 gene:ENSG00000201782 // chr8 // 100 // 77 // 20 // 20 // 0
8148263	1.14E-11	0.7106	chr8	125532243	125534277	5	26	NM_017956 // TRMT12 // tRNA methyltransferase 12 homolog (S. cerevisiae) // 8q24.13 // 55039 /// ENST00000328599 // TRMT12 // tRNA methyltransferase 12 homolog (S. cerevisiae) (TRMT12)
8146448	1.27E-11	0.7094	chr8	55210334	55223014	5	22	NM_014175 // MRPL15 // mitochondrial ribosomal protein L15 // 8q11.2-q13 // 29088 /// ENST00000260102 // MRPL15 // mitochondrial ribosomal protein L15 (MRPL15)
8152096	1.73E-11	0.7062	chr8	102000087	102034745	5	28	NM_145690 // YWHAZ // tyrosine 3-monooxygenase/tryptophan 5-monooxygenase activation protein
8153175	4.04E-11	0.6970	chr8	140811770	141537860	7	31	NM_031466 // NIBP // NIK and IKK{beta} binding protein // 8q24.3 // 83696 /// ENST00000389327 // NIBP // NIK and IKK{beta} binding protein (NIBP)
8152628	5.16E-11	0.6943	chr8	124094769	124123722	5	31	NM_024295 // DERL1 // Der1-like domain family
8027473	8.47E-11	0.6888	chr19	37763944	37770171	5	16	NM_004708 // PDCD5 // programmed cell death 5 // 19q12-q13.1 // 9141 /// ENST00000221784 // PDCD5 // programmed cell death 5 (PDCD5)
8148198	1.33E-10	0.6836	chr8	124498146	124523442	6	33	AK225643 // C8orf32 // chromosome 8 open reading frame 32 // 8q24.13 // 55093
8148467	1.54E-10	0.6818	chr8	141590586	141596434	6	24	uc003yvl.1 // CHRAC1 // chromatin accessibility complex 1 // 8q24.3 // 54108
8027416	2.66E-10	0.6754	chr19	35106391	35198456	6	34	NM_003796 // C19orf2 // chromosome 19 open reading frame 2 // 19q12 // 8725 /// NM_134447 // C19orf2 // chromosome 19 open reading frame 2 // 19q12 // 8725 /// BC026184 // C19orf2 // chromosome 19 open reading frame 2 // 19q12 // 8725
8148265	2.68E-10	0.6753	chr8	125556189	125570040	5	31	NM_007218 // RNF139 // ring finger protein 139 // 8q24 // 11236
8036027	4.74E-10	0.6683	chr19	39110149	39110370	5	24	---

8153223	6.01E-10	0.6654	chr8	141737670	142080514	7	37	NM_153831 // PTK2 // PTK2 protein tyrosine kinase 2 // 8q24-qter // 5747 /// NM_005607 // PTK2 // PTK2 protein tyrosine kinase 2 // 8q24-qter // 5747 /// ENST00000340930 // PTK2 // PTK2 protein tyrosine kinase 2 (PTK2)
8027429	9.11E-10	0.6602	chr19	35212048	35212284	5	21	ENST00000361098 // ENSEMBL Transcript // cdna:known chromosome:NCBI36:19:35212048:35212284:1 gene:ENSG00000196986 // na // 100 // 0 // 21 // --- // 0
8152812	1.36E-09	0.6551	chr8	127633869	127639648	6	30	NM_174911 // FAM84B // family with sequence similarity 84
8151030	1.65E-09	0.6525	chr8	62706791	62707125	5	25	---
8027482	1.68E-09	0.6523	chr19	37902519	37998370	5	30	NM_001015890 // ECAT8 // ES cell associated transcript 8 // 19q13.11 // 91646 /// AB211063 // ECAT8 // ES cell associated transcript 8 // 19q13.11 // 91646 /// ENST00000311695 // ECAT8 // ES cell associated transcript 8 (ECAT8)
8148158	1.70E-09	0.6521	chr8	124154101	124233574	6	28	NM_145647 // WDR67 // WD repeat domain 67 // 8q24.13 // 93594 /// ENST00000287380 // WDR67 // WD repeat domain 67 (WDR67)
8152750	4.21E-09	0.6400	chr8	125393412	125454110	5	35	NM_194291 // TMEM65 // transmembrane protein 65 // 8q24.13 // 157378 /// ENST00000297632 // TMEM65 // transmembrane protein 65 (TMEM65)
8027448	5.99E-09	0.6351	chr19	37588520	37668635	5	45	NM_207325 // DPY19L3 // dpy-19-like 3 (C. elegans) // 19q13.11 // 147991 /// ENST00000342179 // DPY19L3 // dpy-19-like 3 (C. elegans) (DPY19L3)
8146456	6.84E-09	0.6332	chr8	55405800	55406008	5	25	---
8035941	8.51E-09	0.6302	chr19	38013257	38052512	4*	35	NM_014270 // SLC7A9 // solute carrier family 7 (cationic amino acid transporter
8153043	1.71E-08	0.6201	chr8	135559213	135794463	6	28	NM_020863 // ZFAT1 // ZFAT zinc finger 1 // 8q24.22 // 57623 /// NM_001029939 // ZFAT1 // ZFAT zinc finger 1 // 8q24.22 // 57623 /// AB167739 // ZFAT1 // ZFAT zinc finger 1 // 8q24.22 // 57623 /// ENST00000318135 // ZFAT1 // ZFAT zinc finger 1 (ZFAT1)
8148325	1.71E-08	0.6201	chr8	129080559	129080722	7	25	---
8148280	1.95E-08	0.6181	chr8	126079901	126103707	5	37	NM_003129 // SQLE // squalene epoxidase // 8q24.1 // 6713 /// ENST00000265896 // SQLE // squalene epoxidase (SQLE)
8152668	2.97E-08	0.6118	chr8	124401922	124477868	6	37	NM_014109 // ATAD2 // ATPase family
8027431	5.08E-08	0.6036	chr19	35555168	35740805	5	25	NM_014717 // ZNF536 // zinc finger protein 536 // 19q12 // 9745 /// ENST00000355537 // ZNF536 // zinc finger protein 536 (ZNF536)
8153328	5.60E-08	0.6021	chr8	143740950	143744942	5	17	NM_003724 // JRK // jerky homolog (mouse) // 8q24.3 // 8629 /// NM_001077527 // JRK // jerky homolog (mouse) // 8q24.3 // 8629
8148270	6.53E-08	0.5997	chr8	125620092	125631401	5	16	NM_005005 // NDUFB9 // NADH dehydrogenase (ubiquinone) 1 beta subcomplex
8148476	7.53E-08	0.5974	chr8	142207902	142275083	5	37	NM_014957 // DENND3 // DENN/MADD domain containing 3 // 8q24.3 // 22898 /// ENST00000262585 // DENND3 // DENN/MADD domain containing 3 (DENND3)
8027402	9.06E-08	0.5944	chr19	34994741	35007059	6	30	NM_001238 // CCNE1 // cyclin E1 // 19q12 // 898 /// NM_057182 // CCNE1 // cyclin E1 // 19q12 // 898 /// ENST00000262643 // CCNE1 // cyclin E1 (CCNE1)
8153304	1.18E-07	0.5902	chr8	143291348	143482444	5	32	NM_145003 // TSNARE1 // t-SNARE domain containing 1 // 8q24.3 // 203062 /// ENST00000307180 // TSNARE1 // t-SNARE domain containing 1 (TSNARE1)
8148315	1.66E-07	0.5846	chr8	128497039	128498637	6	13	NR_002304 // POU5F1P1 // POU class 5 homeobox 1 pseudogene 1 // 8q24.21 // 5462

8150988	1.78E-07	0.5834	chr8	62578374	62789889	5	61	NM_004318 // ASPH // aspartate beta-hydroxylase // 8q12.1 // 444 /// NM_032468 // ASPH // aspartate beta-hydroxylase // 8q12.1 // 444 /// NM_020164 // ASPH // aspartate beta-hydroxylase // 8q12.1 // 444 /// NM_032467 // ASPH // aspartate beta-hydroxylase // 8q12.1 // 444 /// NM_032466 // ASPH // aspartate beta-hydroxylase // 8q12.1 // 444 /// ENST00000389213 // ASPH // aspartate beta-hydroxylase (ASPH)
8148358	2.42E-07	0.5782	chr8	133856786	133930234	6	53	NM_016018 // PHF20L1 // PHD finger protein 20-like 1 // 8q24.22 // 51105 /// NM_198513 // PHF20L1 // PHD finger protein 20-like 1 // 8q24.22 // 51105 /// NM_032205 // PHF20L1 // PHD finger protein 20-like 1 // 8q24.22 // 51105 /// ENST00000337920 // PHF20L1 // PHD finger protein 20-like 1 (PHF20L1)
8148293	2.67E-07	0.5765	chr8	126173277	126448544	5	33	NM_173685 // NSMCE2 // non-SMC element 2
8153201	2.99E-07	0.5746	chr8	141610446	141714827	6	30	NM_012154 // EIF2C2 // eukaryotic translation initiation factor 2C
8148276	3.24E-07	0.5732	chr8	126054721	126060812	5	27	NM_152412 // ZNF572 // zinc finger protein 572 // 8q24.13 // 137209 /// ENST00000319286 // ZNF572 // zinc finger protein 572 (ZNF572)
8146458	3.66E-07	0.5711	chr8	55495581	55495806	5	18	---
8027383	6.91E-07	0.5599	chr19	34184414	34184503	5	25	---
8148501	7.20E-07	0.5591	chr8	142501189	142510802	5	28	NM_032611 // PTP4A3 // protein tyrosine phosphatase type IVA
8146625	7.96E-07	0.5573	chr8	63324055	64074765	5	28	ENST00000328472 // FAM77D // family with sequence similarity 77
8035880	9.10E-07	0.5549	chr19	34390007	34395954	5	9	NM_006003 // UQCRFS1 // ubiquinol-cytochrome c reductase
8152656	9.13E-07	0.5548	chr8	124329877	124355728	5	32	NM_001017926 // ZHX1 // zinc fingers and homeoboxes 1 // 8q24.13 // 11244 /// NM_007222 // ZHX1 // zinc fingers and homeoboxes 1 // 8q24.13 // 11244 /// ENST00000297857 // ZHX1 // zinc fingers and homeoboxes 1 (ZHX1)
8027398	9.29E-07	0.5545	chr19	34847803	34858224	6	26	NM_024310 // PLEKHF1 // pleckstrin homology domain containing
8152900	9.63E-07	0.5538	chr8	131150964	131151080	6	11	ENST00000363434 // ENSEMBL ncRNA // ncrna:snRNA chromosome:NCBI36:8:131150969:131151080:-1 gene:ENSG00000200304 // chr8 // 100 // 100 // 11 // 11 // 0
8148208	1.21E-06	0.5496	chr8	124849884	124896873	5	11	NM_144963 // FAM91A1 // family with sequence similarity 91
8148184	1.59E-06	0.5445	chr8	124261580	124291499	5	28	NM_207006 // FAM83A // family with sequence similarity 83
8152782	1.97E-06	0.5404	chr8	126105690	126173191	5	35	NM_014846 // KIAA0196 // KIAA0196 // 8q24.13 // 9897 /// ENST00000318410 // KIAA0196 // KIAA0196 (KIAA0196)
8027564	2.24E-06	0.5379	chr19	38485617	38487800	5	25	BC027959 // FLJ12355 // hypothetical protein FLJ12355 // 19q13.11 // 80054
8035896	2.70E-06	0.5342	chr19	36457691	36531984	6	24	NM_020856 // TSHZ3 // teashirt zinc finger homeobox 3 // 19q12 // 57616 /// ENST00000240587 // TSHZ3 // teashirt family zinc finger 3 (TSHZ3)
8152666	2.79E-06	0.5336	chr8	124348065	124348166	5	25	---
8148515	3.81E-06	0.5274	chr8	142526758	142526831	5	25	---
8152664	3.90E-06	0.5270	chr8	124339906	124339978	5	25	---
8061579	4.70E-06	0.5232	chr20	29790565	29853264	5	28	NM_012112 // TPX2 // TPX2
8027584	5.92E-06	0.5185	chr19	38979607	38996988	5	24	NM_024076 // KCTD15 // potassium channel tetramerisation domain containing 15 // 19q13.11 // 79047 /// ENST00000284006 // KCTD15 // potassium channel tetramerisation domain containing 15 (KCTD15)
8152759	1.24E-05	0.5029	chr8	125569930	125620510	5	9	NM_032026 // TATDN1 // TatD DNase domain containing 1 // 8q24.13 // 83940 /// ENST00000276692 // TATDN1 // TatD DNase domain containing 1 (TATDN1)

8148559	1.32E-05	0.5015	chr8	143805623	143815352	5	33	NM_016647 // C8orf55 // chromosome 8 open reading frame 55 // 8q24.3 // 51337 /// AK055140 // C8orf55 // chromosome 8 open reading frame 55 // 8q24.3 // 51337
8152703	1.56E-05	0.4979	chr8	124584539	124622627	6	28	NM_058229 // FBXO32 // F-box protein 32 // 8q24.13 // 114907 /// NM_148177 // FBXO32 // F-box protein 32 // 8q24.13 // 114907 /// ENST00000287396 // FBXO32 // F-box protein 32 (FBXO32)
8146460	2.05E-05	0.4919	chr8	55496033	55496120	5	25	---
8027437	3.27E-05	0.4812	chr19	36332202	36332597	5	4	---
8092201	3.30E-05	0.4810	chr3	178221236	178397734	5	18	NM_024665 // TBL1XR1 // transducin (beta)-like 1X-linked receptor 1 // 3q26.32 // 79718 /// ENST00000314694 // TBL1XR1 // transducin (beta)-like 1X-linked receptor 1 (TBL1XR1)
8148385	3.56E-05	0.4792	chr8	133948387	134216340	6	62	NM_003235 // TG // thyroglobulin // 8q24 // 7038 /// ENST00000220616 // TG // thyroglobulin (TG)
8152845	4.88E-05	0.4718	chr8	130922898	131097829	6	37	ENST00000358017 // FAM49B // family with sequence similarity 49
8027480	5.80E-05	0.4676	chr19	37858153	37861046	5	18	NM_207391 // RGS9BP // regulator of G protein signaling 9 binding protein // 19q13.11 // 388531 /// ENST00000334176 // RGS9BP // regulator of G protein signalling 9 binding protein (RGS9BP)
8084016	5.94E-05	0.4670	chr3	180349005	180435616	5	37	NM_006218 // PIK3CA // phosphoinositide-3-kinase
8148149	6.99E-05	0.4631	chr8	123863082	124055936	5	28	NM_014943 // ZHX2 // zinc fingers and homeoboxes 2 // 8q24.13 // 22882 /// ENST00000314393 // ZHX2 // zinc fingers and homeoboxes 2 (ZHX2)
8152867	7.11E-05	0.4627	chr8	131133535	131483399	6	41	NM_018482 // DDEF1 // development and differentiation enhancing factor 1 // 8q24.1-q24.2 // 50807 /// ENST00000357668 // DDEF1 // development and differentiation enhancing factor 1 (DDEF1)
8035993	7.84E-05	0.4603	chr19	38391410	38408596	5	28	NM_019849 // SLC7A10 // solute carrier family 7
8061517	0.000104	0.4532	chr20	29427783	29427911	5	25	ENST00000390832 // ENSEMBL ncRNA // ncrna:snoRNA chromosome:NCBI36:20:29427783:29427910:1 gene:ENSG00000212134 // chr20 // 100 // 96 // 24 // 24 // 0
8148435	0.000137	0.4462	chr8	134272494	134310753	6	30	NM_003882 // WISP1 // WNT1 inducible signaling pathway protein 1 // 8q24.1-q24.3 // 8840 /// NM_080838 // WISP1 // WNT1 inducible signaling pathway protein 1 // 8q24.1-q24.3 // 8840 /// ENST00000250160 // WISP1 // WNT1 inducible signaling pathway protein 1 (WISP1)
8065555	0.000209	0.4351	chr20	29428628	29442067	5	27	NM_173460 // DEFB119 // defensin
8152828	0.000255	0.4298	chr8	130829624	130868316	6	38	NM_031415 // MLZE // melanoma-derived leucine zipper
8148333	0.000378	0.4189	chr8	132985549	133094956	6	27	ENST00000254624 // KIAA0143 // KIAA0143 protein (KIAA0143)
8027556	0.000492	0.4114	chr19	38377330	38390386	5	24	NM_002333 // LRP3 // low density lipoprotein receptor-related protein 3 // 19q13.11 // 4037 /// ENST00000253193 // LRP3 // low density lipoprotein receptor-related protein 3 (LRP3)
8152865	0.000514	0.4101	chr8	130956698	130959352	6	20	AB062477 // GenBank // Homo sapiens OK/SW-cl.41 mRNA
8148448	0.000539	0.4087	chr8	136538884	136729035	7	33	NM_006558 // KHDRBS3 // KH domain containing
8148553	0.000541	0.4087	chr8	143778533	143782613	5	29	NM_017527 // LY6K // lymphocyte antigen 6 complex
8153258	0.00057	0.4071	chr8	142286455	142287964	5	6	ENST00000377746 // ENSEMBL Transcript // cdna:known chromosome:NCBI36:8:142286845:142287423:-1 gene:ENSG00000204886 // chr8 // 100 // 50 // 3 // 3 // 0
8153002	0.000757	0.3988	chr8	134318596	134379011	6	45	NM_006096 // NDRG1 // N-myc downstream regulated gene 1 // 8q24.3 // 10397 /// ENST00000388848 // NDRG1 // N-myc downstream regulated gene 1 (NDRG1)
8152821	0.000842	0.3955	chr8	130433122	130761667	7	28	BC070152 // CCDC26 // coiled-coil domain containing 26 // 8q24.21 // 137196 /// BC026098 // CCDC26 // coiled-coil domain containing 26 // 8q24.21 // 137196
8153262	0.000866	0.3947	chr8	142290052	142307855	6	18	NM_001080431 // SLC45A4 // solute carrier family 45

8152863	0.000925	0.3927	chr8	130950018	130950144	6	25	ENST00000363205 // ENSEMBL ncRNA // ncrna:snoRNA chromosome:NCBI36:8:130950018:130950144:-1 gene:ENSG00000200075 // chr8 // 100 // 100 // 25 // 25 // 0
8061509	0.000941	0.3922	chr20	29420082	29425364	4*	29	NM_054112 // DEFB118 // defensin
8148302	0.000951	0.3919	chr8	126352028	126352326	5	21	---
8152946	0.000997	0.3904	chr8	133210438	133562186	6	36	NM_004519 // KCNQ3 // potassium voltage-gated channel
8152924	0.001153	0.3859	chr8	133105910	133136455	6	30	NM_001080399 // OC90 // otoconin 90 // 8q24.22 // 729330 /// ENST00000254627 // OC90 // otoconin 90 (OC90)
8153067	0.001215	0.3843	chr8	135886301	135886370	6	21	hsa-mir-30d // miRBase Micro RNA Database // MI0000255 Homo sapiens miR-30d stem-loop // chr8 // 100 // 100 // 21 // 21 // 0
8153069	0.00125	0.3834	chr8	136823743	136823920	6	21	---
8153021	0.001326	0.3815	chr8	134540312	134653349	6	27	NM_003033 // ST3GAL1 // ST3 beta-galactoside alpha-2
8148329	0.00141	0.3796	chr8	130372411	130372654	7	25	---
8153346	0.001785	0.3720	chr8	143842758	143856642	5	44	NM_023946 // LYNX1 // Ly6/neurotoxin 1 // 8q24.3 // 66004 /// NM_177457 // LYNX1 // Ly6/neurotoxin 1 // 8q24.3 // 66004 /// NM_177477 // LYNX1 // Ly6/neurotoxin 1 // 8q24.3 // 66004 /// NM_177476 // LYNX1 // Ly6/neurotoxin 1 // 8q24.3 // 66004 /// NM_177458 // LYNX1 // Ly6/neurotoxin 1 // 8q24.3 // 66004 /// ENST00000345173 // LYNX1 // Ly6/neurotoxin 1 (LYNX1)
8148194	0.001848	0.3709	chr8	124344264	124344352	5	25	ENST00000390925 // ENSEMBL ncRNA // ncrna:snRNA chromosome:NCBI36:8:124344264:124344370:1 gene:ENSG00000212227 // chr8 // 100 // 96 // 24 // 24 // 0
8036025	0.001948	0.3692	chr19	38771952	38772443	5	24	ENST00000310822 // ENSEMBL Transcript // cdna:known chromosome:NCBI36:19:38771952:38772443:-1 gene:ENSG00000173772 // chr19 // 100 // 100 // 24 // 24 // 0
8153363	0.001956	0.3690	chr8	143950775	143958238	5	23	NM_000497 // CYP11B1 // cytochrome P450
8148461	0.001982	0.3686	chr8	140189855	140189978	5	25	---
8153101	0.002541	0.3603	chr8	139669660	139995418	5	69	NM_152888 // COL22A1 // collagen
8152626	0.002605	0.3595	chr8	123955490	123955596	5	25	---
8035935	0.002709	0.3582	chr19	37791083	37791215	5	23	ENST00000364518 // ENSEMBL ncRNA // ncrna:snoRNA chromosome:NCBI36:19:37791083:37791215:-1 gene:ENSG00000201388 // chr19 // 100 // 100 // 23 // 23 // 0
8153071	0.003503	0.3493	chr8	139211448	139578247	5	61	NM_015912 // FAM135B // family with sequence similarity 135
8148465	0.003557	0.3488	chr8	141375899	141376162	6	25	ENST00000323118 // ENSEMBL Transcript // cdna:known chromosome:NCBI36:8:141375899:141376162:1 gene:ENSG00000179503 // chr8 // 100 // 100 // 25 // 25 // 0
8061529	0.003642	0.3480	chr20	29526766	29536369	5	26	NM_014012 // REM1 // RAS (RAD and GEM)-like GTP-binding 1 // 20q11.21 // 28954 /// ENST00000201979 // REM1 // RAS (RAD and GEM)-like GTP-binding 1 (REM1)
8152962	0.003938	0.3452	chr8	133653629	133756995	6	28	NM_012472 // LRRC6 // leucine rich repeat containing 6 // 8q24.22 // 23639 /// ENST00000250173 // LRRC6 // leucine rich repeat containing 6 (LRRC6)
8148156	0.00416	0.3433	chr8	124126138	124126231	5	4	---
8153167	0.004714	0.3388	chr8	140693986	140784481	5	26	NM_016601 // KCNK9 // potassium channel
8152819	0.005012	0.3366	chr8	129091903	129092039	7	26	---

8065563	0.005677	0.3320	chr20	29456361	29464107	5	26	NM_001011878 // DEFB121 // defensin
8148463	0.006206	0.3287	chr8	141012598	141015362	5	25	AF220264 // C8orf17 // chromosome 8 open reading frame 17 // 8q24.3 // 56988
8152988	0.006649	0.3261	chr8	134118155	134184479	6	26	NM_001045556 // SLA // Src-like-adaptor // 8q22.3-qter|8q24 // 6503 /// NM_006748 // SLA // Src-like-adaptor // 8q22.3-qter|8q24 // 6503 /// NM_001045557 // SLA // Src-like-adaptor // 8q22.3-qter|8q24 // 6503 /// ENST00000338087 // SLA // Src-like-adaptor (SLA)
8153035	0.007167	0.3233	chr8	134726022	134726150	6	22	ENST00000390971 // ENSEMBL ncRNA // ncrna:snoRNA chromosome:NCBI36:8:134726023:134726150:-1 gene:ENSG00000212273 // chr8 // 100 // 95 // 21 // 21 // 0
8153041	0.007615	0.3210	chr8	134837126	134837192	6	12	ENST00000385766 // ENSEMBL ncRNA // ncrna:Mt_tRNA_pseudogene chromosome:NCBI36:8:134837126:134837192:-1 gene:ENSG00000208501 // chr8 // 100 // 92 // 11 // 11 // 0
8148565	0.007766	0.3202	chr8	143913219	143925264	5	29	NM_002066 // GML // GPI anchored molecule like protein // 8q24.3 // 2765 /// ENST00000220940 // GML // GPI anchored molecule like protein (GML)
8148304	0.009136	0.3139	chr8	126511745	126519827	5	28	NM_025195 // TRIB1 // tribbles homolog 1 (Drosophila) // 8q24.13 // 10221 /// ENST00000311922 // TRIB1 // tribbles homolog 1 (Drosophila) (TRIB1)
8147689	0.010637	0.3079	chr8	102039330	102039584	5	24	---
8035939	0.010709	0.3076	chr19	37837829	37838054	5	22	---
8092239	0.011077	0.3063	chr3	180369567	180369692	4*	25	ENST00000365087 // ENSEMBL ncRNA // ncrna:snoRNA chromosome:NCBI36:3:180369567:180369692:-1 gene:ENSG00000201957 // chr3 // 100 // 100 // 25 // 25 // 0
8152642	0.011141	0.3061	chr8	124282593	124284164	5	29	AY225521 // GenBank // Homo sapiens HCC-related HCC-C11_v3 mRNA
8148309	0.012296	0.3021	chr8	126982377	126982480	6	25	---
8148548	0.01298	0.2999	chr8	143758877	143761145	5	26	NM_005672 // PSCA // prostate stem cell antigen // 8q24.2 // 8000 /// AF043498 // PSCA // prostate stem cell antigen // 8q24.2 // 8000 /// ENST00000301258 // PSCA // prostate stem cell antigen (PSCA)
8152734	0.014883	0.2942	chr8	125065559	125122204	5	25	AK093040 // C8orf54 // chromosome 8 open reading frame 54 // 8q24.13 // 439941
8152742	0.015467	0.2926	chr8	125127495	125252944	5	27	AK057332 // C8orf78 // chromosome 8 open reading frame 78 // 8q24.13 // 157376
8152640	0.015937	0.2913	chr8	124261731	124261945	5	24	---
8152922	0.016854	0.2890	chr8	132514205	132514384	6	25	ENST00000362726 // ENSEMBL ncRNA // ncrna:snoRNA chromosome:NCBI36:8:132514205:132514384:-1 gene:ENSG00000199596 // chr8 // 100 // 100 // 25 // 25 // 0
8153273	0.027568	0.2673	chr8	142513114	142586512	5	37	NM_207414 // FLJ43860 // FLJ43860 protein // 8q24.3 // 389690 /// AK131461 // FLJ43860 // FLJ43860 protein // 8q24.3 // 389690
8152764	0.028749	0.2653	chr8	125632212	125809840	5	29	NM_014751 // MTSS1 // metastasis suppressor 1 // 8p22 // 9788 /// ENST00000325064 // MTSS1 // metastasis suppressor 1 (MTSS1)
8061519	0.029656	0.2639	chr20	29492014	29501721	5	27	NM_153324 // DEFB123 // defensin
8152938	0.029756	0.2638	chr8	133145214	133162535	6	34	ENST00000262283 // ENSEMBL Transcript // HERV-H LTR-associating 1 (HHLA1)
8152715	0.031193	0.2616	chr8	124727160	124734347	5	28	ENST00000325995 // ENSEMBL Transcript // hypothetical protein LOC340359 (C8ORFK36)
8148474	0.0337	0.2579	chr8	142019832	142019945	6	25	---

8027385	0.033819	0.2578	chr19	34709331	34713045	6	21	ENST00000335523 // ENSEMBL Transcript // cdna:known chromosome:NCBI36:19:34709331:34713045:1 gene:ENSG00000187135 // chr19 // 100 // 100 // 21 // 21 // 0
8092220	0.035943	0.2549	chr3	178326499	178326602	5	24	---
8152976	0.038544	0.2515	chr8	133791373	133842010	6	32	ENST00000356838 // TMEM71 // transmembrane protein 71 (TMEM71)
8027574	0.039631	0.2502	chr19	38804717	38956254	5	36	NM_022467 // CHST8 // carbohydrate (N-acetylgalactosamine 4-0) sulfotransferase 8 // 19q13.1 // 64377 /// ENST00000262622 // CHST8 // carbohydrate (N-acetylgalactosamine 4-0) sulfotransferase 8 (CHST8)
8150842	0.040555	0.2491	chr8	55266694	55267149	5	25	ENST00000331419 // ENSEMBL Transcript // cdna:known chromosome:NCBI36:8:55266694:55267149:-1 gene:ENSG00000182699 // chr8 // 100 // 100 // 25 // 25 // 0
8148196	0.052934	0.2358	chr8	124423835	124423903	6	15	---
8065569	0.05883	0.2303	chr20	29715916	29774366	5	26	NM_138578 // BCL2L1 // BCL2-like 1 // 20q11.21 // 598 /// NM_001191 // BCL2L1 // BCL2-like 1 // 20q11.21 // 598 /// ENST00000376055 // BCL2L1 // BCL2-like 1 (BCL2L1)
8092218	0.059201	0.2300	chr3	178321175	178321282	5	25	---
8153320	0.061632	0.2279	chr8	143326852	143327134	5	22	---
8148261	0.065211	0.2249	chr8	125342068	125342174	5	24	---
8061539	0.066441	0.2239	chr20	29537334	29538662	5	23	ENST00000278994 // ENSEMBL Transcript // cdna:novel chromosome:NCBI36:20:29537334:29538662:1 gene:ENSG00000149611 // chr20 // 100 // 100 // 23 // 23 // 0
8148317	0.067117	0.2234	chr8	128817498	128822856	6	26	NM_002467 // MYC // v-myc myelocytomatosis viral oncogene homolog (avian) // 8q24.21 // 4609 /// ENST00000377970 // MYC // v-myc myelocytomatosis viral oncogene homolog (avian) (MYC)
8152701	0.069215	0.2217	chr8	124536398	124536528	6	14	---
8148331	0.074763	0.2175	chr8	131816260	131816330	6	4	---
8153065	0.076589	0.2162	chr8	135881945	135882032	6	22	hsa-mir-30b // miRBase Micro RNA Database // MI0000441 Homo sapiens miR-30b stem-loop // chr8 // 100 // 100 // 22 // 22 // 0
8061542	0.082478	0.2121	chr20	29565892	29621031	5	47	NM_178580 // HM13 // histocompatibility (minor) 13 // 20q11.21 // 81502 /// NM_178581 // HM13 // histocompatibility (minor) 13 // 20q11.21 // 81502 /// NM_030789 // HM13 // histocompatibility (minor) 13 // 20q11.21 // 81502 /// NM_178582 // HM13 // histocompatibility (minor) 13 // 20q11.21 // 81502 /// ENST00000335574 // HM13 // histocompatibility (minor) 13 (HM13)
8061572	0.086416	0.2095	chr20	29689352	29696470	5	28	NM_032609 // COX4I2 // cytochrome c oxidase subunit IV isoform 2 (lung) // 20q11.21 // 84701 /// ENST00000376075 // COX4I2 // cytochrome c oxidase subunit IV isoform 2 (lung) (COX4I2)
8065566	0.095648	0.2037	chr20	29516970	29524477	5	26	NM_001037500 // DEFB124 // defensin
8153336	0.096091	0.2035	chr8	143819364	143820831	5	28	NM_020427 // SLURP1 // secreted LY6/PLAUR domain containing 1 // 8q24.3 // 57152 /// ENST00000246515 // SLURP1 // secreted LY6/PLAUR domain containing 1 (SLURP1)
8148517	0.10604	0.1977	chr8	143542379	143623370	5	43	NM_001702 // BAI1 // brain-specific angiogenesis inhibitor 1 // 8q24 // 575 /// ENST00000323289 // BAI1 // brain-specific angiogenesis inhibitor 1 (BAI1)
8153269	0.111899	0.1945	chr8	142435767	142446547	5	22	NM_005293 // GPR20 // G protein-coupled receptor 20 // 8q24.2-q24.3 // 2843 /// ENST00000377741 // GPR20 // G protein-coupled receptor 20 (GPR20)
8035884	0.118294	0.1912	chr19	34415837	34416132	5	25	---
8153334	0.127943	0.1864	chr8	143759705	143760100	5	24	AK092432 // PSCA // prostate stem cell antigen // 8q24.2 // 8000

8146462	0.128227	0.1863	chr8	55533048	55535517	4*	27	NM_022454 // SOX17 // SRY (sex determining region Y)-box 17 // 8q11.23 // 64321 /// ENST00000297316 // SOX17 // SRY (sex determining region Y)-box 17 (SOX17)
8148206	0.131244	0.1849	chr8	124663658	124663728	6	25	---
8153037	0.158618	0.1729	chr8	134836956	134837024	6	24	ENST00000385768 // ENSEMBL ncRNA // ncrna:Mt_tRNA_pseudogene chromosome:NCBI36:8:134836956:134837024:-1 gene:ENSG00000208503 // chr8 // 100 // 100 // 24 // 24 // 0
8152902	0.178472	0.1651	chr8	131861729	132123854	6	29	NM_001115 // ADCY8 // adenylate cyclase 8 (brain) // 8q24 // 114 /// ENST00000286355 // ADCY8 // adenylate cyclase 8 (brain) (ADCY8)
8061562	0.191005	0.1605	chr20	29632553	29632668	5	24	---
8153359	0.211829	0.1534	chr8	143863300	143865010	5	27	NM_003695 // LY6D // lymphocyte antigen 6 complex
8153342	0.212389	0.1532	chr8	143828630	143830954	5	27	NM_205545 // LYPD2 // LY6/PLAUR domain containing 2 // 8q24.3 // 137797 /// ENST00000359228 // LYPD2 // LY6/PLAUR domain containing 2 (LYPD2)
8027414	0.222079	0.1500	chr19	35103929	35104399	6	21	ENST00000355049 // ENSEMBL Transcript // similar to peptidylprolyl isomerase A isoform 1 (LOC126170)
8035937	0.268652	0.1360	chr19	37837423	37837691	5	17	---
8153373	0.360995	0.1125	chr8	143988977	143996261	5	24	NM_000498 // CYP11B2 // cytochrome P450
8153322	0.399208	0.1039	chr8	143689412	143692835	5	30	NM_015193 // ARC // activity-regulated cytoskeleton-associated protein // 8q24.3 // 23237 /// ENST00000356613 // ARC // activity-regulated cytoskeleton-associated protein (ARC)
8152719	0.453276	0.0925	chr8	124762215	124818828	5	30	NM_001003954 // ANXA13 // annexin A13 // 8q24.13 // 312 /// NM_004306 // ANXA13 // annexin A13 // 8q24.13 // 312 /// ENST00000262219 // ANXA13 // annexin A13 (ANXA13)
8148512	0.483561	0.0864	chr8	142513827	142517232	5	8	ENST00000355850 // ENSEMBL Transcript // cdna:known chromosome:NCBI36:8:142513827:142517232:1 gene:ENSG00000184334 // chr8 // 100 // 100 // 8 // 8 // 0
8036004	0.560592	0.0718	chr19	38482690	38485270	5	29	NM_004364 // CEBPA // CCAAT/enhancer binding protein (C/EBP)
8148220	0.972111	-0.0043	chr8	124933408	125201483	5	48	NM_001039112 // FER1L6 // fer-1-like 6 (C. elegans) // --- // 654463
8061564	0.959377	-0.0063	chr20	29656747	29657979	5	32	NM_181353 // ID1 // inhibitor of DNA binding 1
8153039	0.741517	-0.0407	chr8	134837037	134837098	6	36	ENST00000385767 // ENSEMBL ncRNA // ncrna:Mt_tRNA_pseudogene chromosome:NCBI36:8:134837037:134837098:-1 gene:ENSG00000208502 // chr8 // 100 // 94 // 34 // 34 // 0 /// ENST00000385831 // ENSEMBL ncRNA // ncrna:Mt_tRNA_pseudogene chromosome:NCBI36:2:117500310:117500382:-1 gene:ENSG00000208566 // chr8 // 86 // 97 // 30 // 35 // 0 /// ENST00000385604 // ENSEMBL ncRNA // ncrna:Mt_tRNA_pseudogene chromosome:NCBI36:7:141148515:141148587:1 gene:ENSG00000208339 // chr8 // 60 // 97 // 21 // 35 // 0 /// ENST00000388363 // ENSEMBL ncRNA // ncrna:Mt_tRNA_pseudogene chromosome:NCBI36:X:55222073:55222145:1 gene:ENSG00000211098 // chr8 // 40 // 97 // 14 // 35 // 1 /// ENST00000385967 // ENSEMBL ncRNA // ncrna:Mt_tRNA_pseudogene chromosome:NCBI36:8:112016133:112016204:1 gene:ENSG00000208702 // chr8 // 24 // 94 // 8 // 34 // 1 /// ENST00000385986 // ENSEMBL ncRNA // ncrna:Mt_tRNA_pseudogene chromosome:NCBI36:3:154120272:154120344:1 gene:ENSG00000208721 // chr8 // 9 // 97 // 3 // 35 // 1

8148446	0.437147	-0.0958	chr8	134836879	134836946	6	8	ENST00000388256 // ENSEMBL ncRNA // ncrna:Mt_tRNA_pseudogene chromosome:NCBI36:11:102782085:102782152:-1 gene:ENSG00000210991 // chr8 // 100 // 100 // 8 // 8 // 0 /// ENST00000387858 // ENSEMBL ncRNA // ncrna:Mt_tRNA_pseudogene chromosome:NCBI36:8:32994108:32994175:1 gene:ENSG00000210593 // chr8 // 100 // 100 // 8 // 8 // 0 /// ENST00000385769 // ENSEMBL ncRNA // ncrna:Mt_tRNA_pseudogene chromosome:NCBI36:8:134836879:134836946:1 gene:ENSG00000208504 // chr8 // 100 // 100 // 8 // 8 // 0 /// ENST00000388466 // ENSEMBL ncRNA // ncrna:Mt_tRNA_pseudogene chromosome:NCBI36:9:82369696:82369762:-1 gene:ENSG00000211201 // chr8 // 63 // 100 // 5 // 8 // 0

* These genes were highly amplified in 4 samples, but were within 10 kb of a copy number breakpoint of 5 amplified samples
