# Supplementary figures and images for: Identification of Candidate Growth Promoting Genes in Ovarian Cancer through Integrated Copy Number and Expression Analysis
Source: PLoS One. 2010 Apr 8;5(4):e9983. doi: 10.1371/journal.pone.0009983 (PMC2851616; doi:10.1371/journal.pone.0009983)

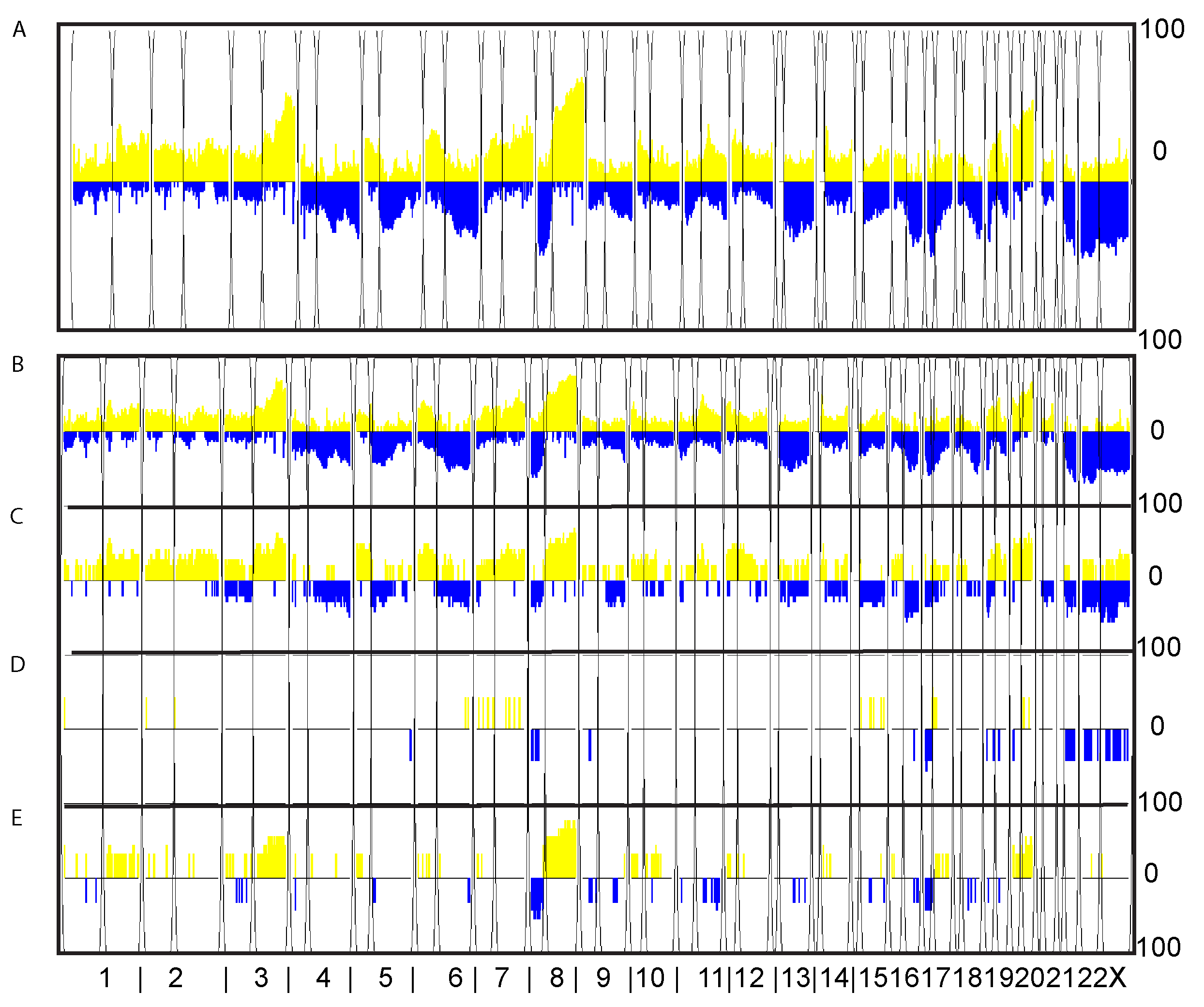

Supplement: Figure S1 — Subtype breakdown of genome wide CN changes. (A) Overall copy number landscape for the cohort of ovarian cancer samples. This is similar to Figure 1 with the exception that the y-axis ranges from 0–100% of samples as opposed to 0–50%. Below are the distribution of copy number changes for (B) 37 serous ovarian cancers, (C) 14 endometrioid ovarian cancers, (D) 7 mucinous ovarian cancers and (E) 9 clear cell ovarian cancers. A, B and C jointly show that the major contributors for the high frequency changes are serous and endometrioid tumours. Data for the single tumor classified as undifferentiated is not shown here. (0.43 MB TIF) [file pone.0009983.s007.tif]

A

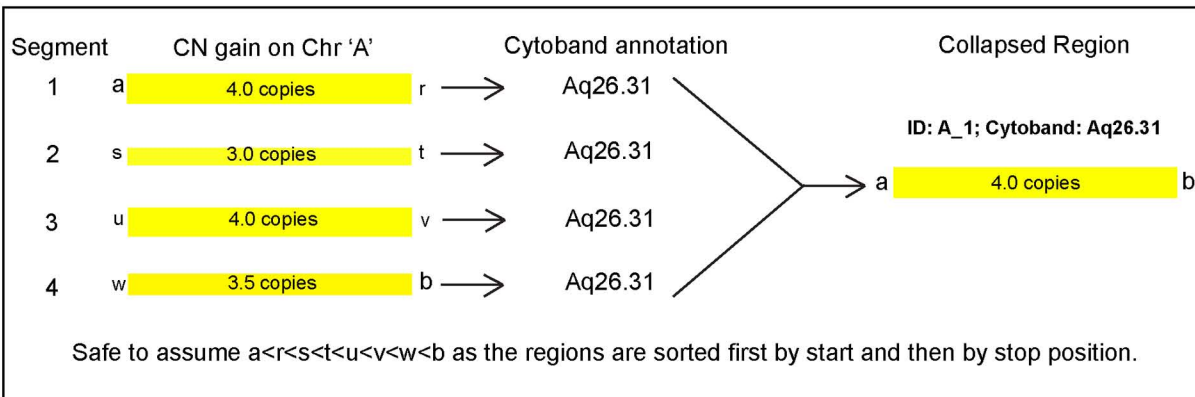

B

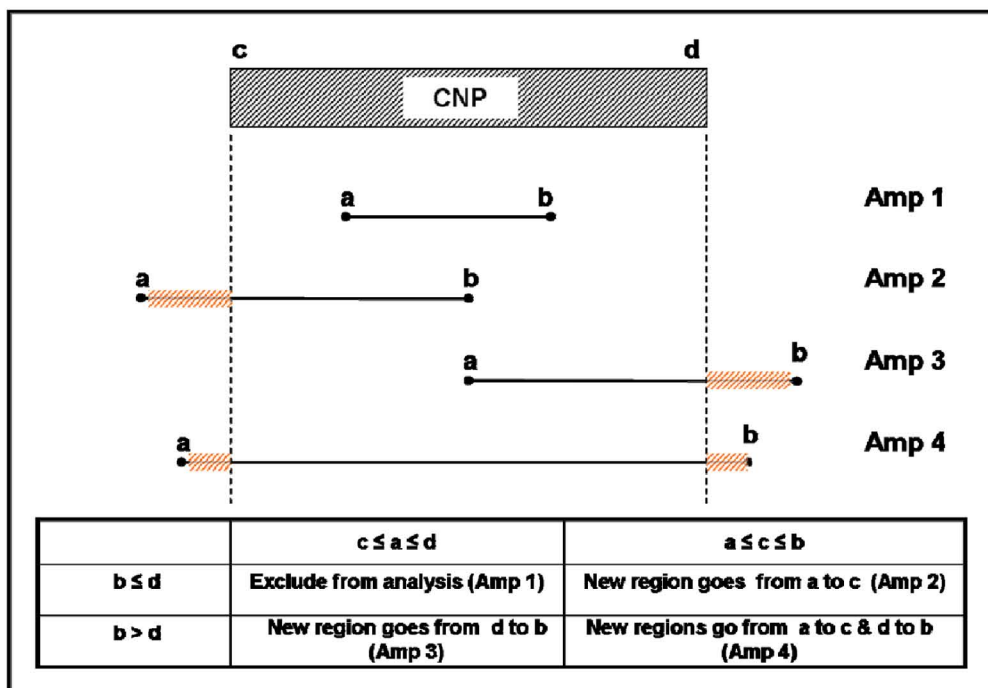

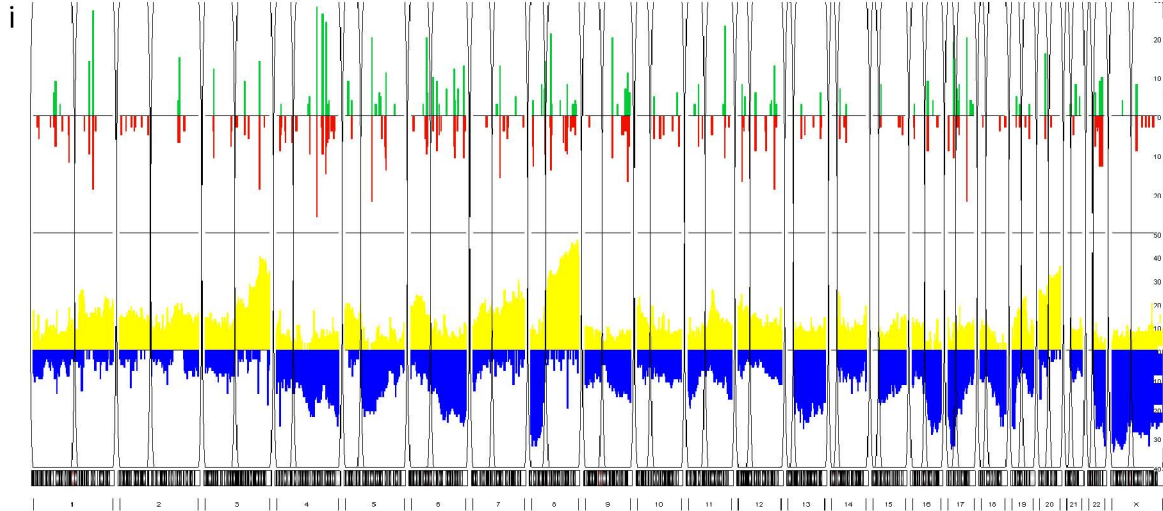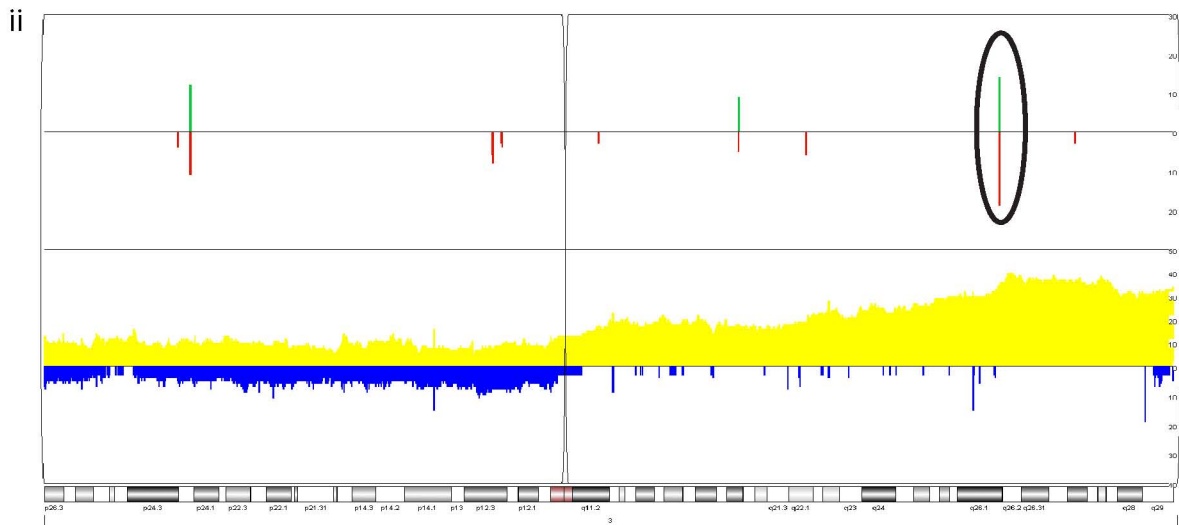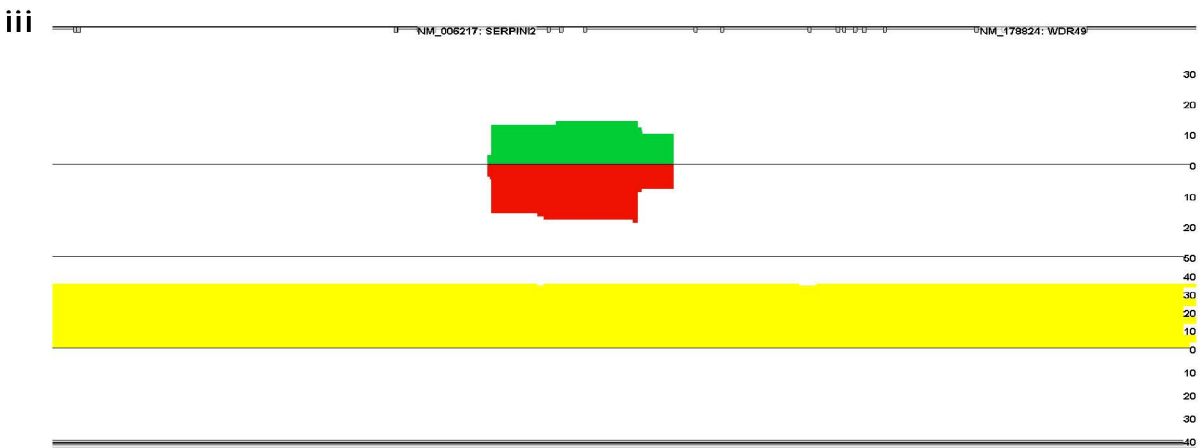

Supplement: Figure S2 — ‘Cytoband collapsing’ and the exclusion of CNPs. (A) Shows the steps taken towards obtaining the copy number regions. The starting data (far left) contains genomic position and copy number information for segmental overlaps. All segments at this step of analysis occur with >40% frequency and have 3 or more copies. Letters a, b, r, s, t, u, v and w refer to genomic start/stop sites in basepairs. Regions are sorted by chromosome, then by genomic start and finally by genomic stop positions. Following this they are annotated with their cytobands and the newly defined “collapsed” region is bounded by the lowest start (a) and highest stop (b) positions and annotated with the cytoband of origin. The ‘a’ and ‘b’ from here carry through to part B of the figure. Regions that span two cytobands are listed as a separate group as shown in Table S4. (B) Shows the rules used to eliminate CNPs from the cytoband regions. Regions such as “Amp 4” are split into two, resulting in more regions after CNP elimination than before. (C) Regions of CNP across the genome and their position in relation to regions of copy number gain relevant to our study. (i) Global changes in normal (n = 57, green = gain and red = loss) and tumour (n = 72, yellow = gain and blue = loss) samples. We define a CNP as a change that occurs in at least 5% of normal samples. CNPs often show both genomic gain and loss at the same locus in normal samples. (ii) All changes on Chromosome 3 and in particular a CNP on 3q26.1 between 168.66 and 168.69 Mbp highlighted by the black oval, observed in >15% of all normal samples. (iii) The 3q26.1 CNP occurs in the middle of a region of copy number gain that we investigate further. This CNP region was removed from the data in accordance with S2-B. (0.55 MB PDF) [file pone.0009983.s008.pdf]

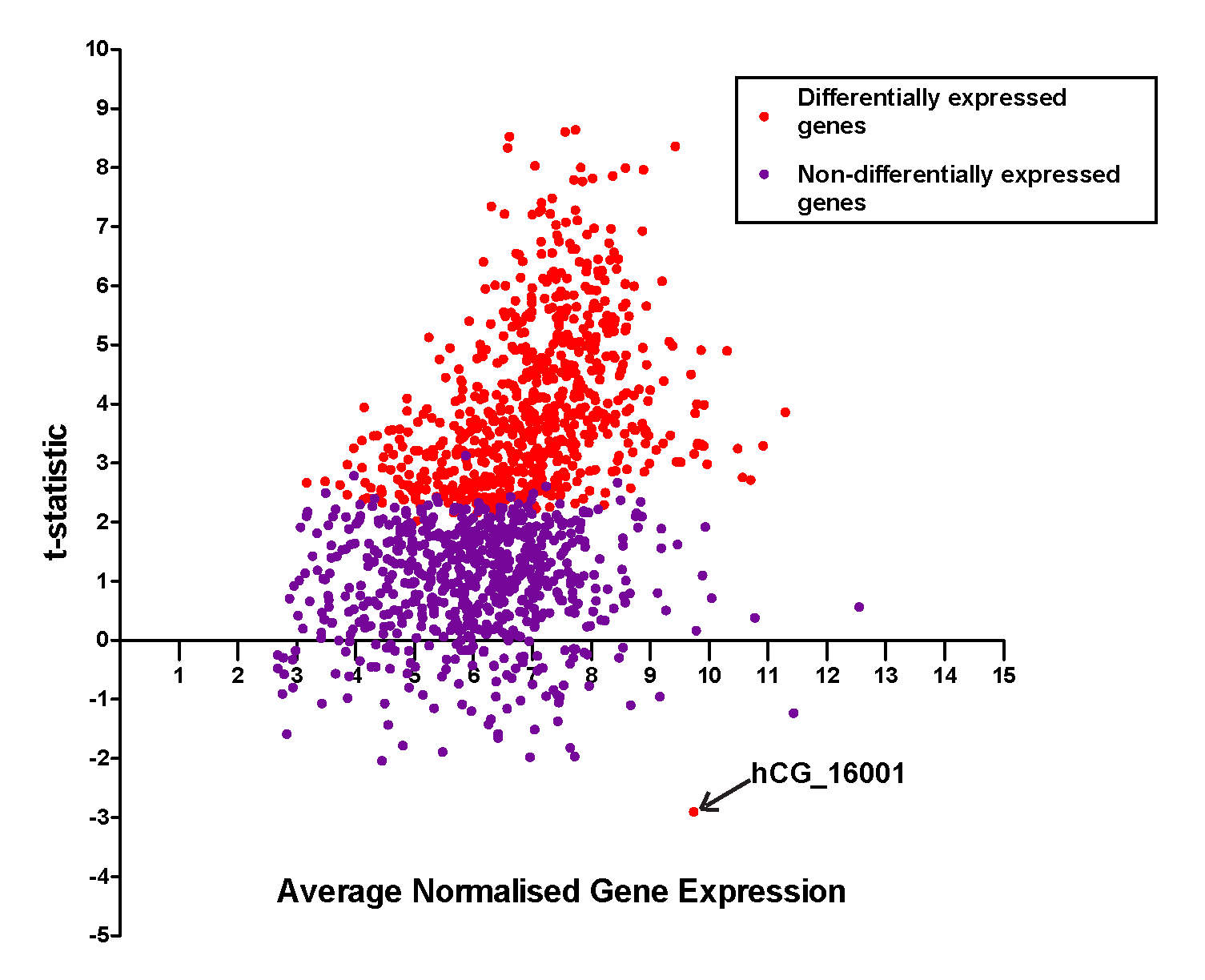

Supplement: Figure S3 — Expression of all genes in regions of frequent copy number gain. This figure displays all genes in 90 regions of copy number change in terms of their average expression and t-statistic, resulting from the test for differential expression for each of these regions between amplified and unamplified samples. Genes showing a significant differential expression are represented by red dots and non-significant genes are represented by purple dots. Only one gene hCG_16001 showed a significant reduction in expression under the influence of copy number gain. This is a ribosomal protein L23a pseudogene 42 (RPL23A42) where RPL23A encodes a ribosomal protein that is a component of the 60S subunit and may be one of the target molecules involved in mediating growth inhibition by interferon. (0.09 MB TIF) [file pone.0009983.s009.tif]

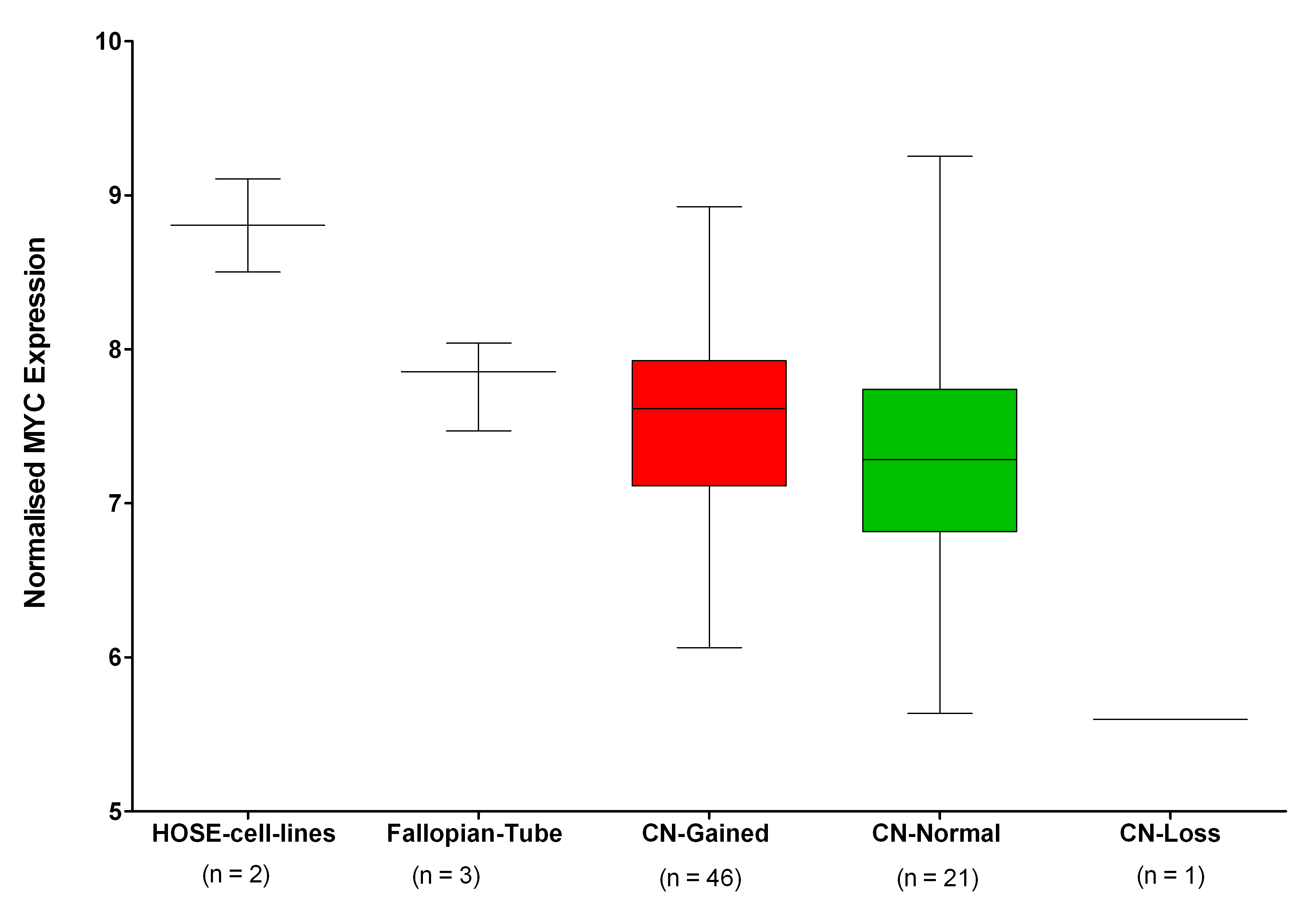

Supplement: Figure S4 — Expression of MYC across various sample groups. RMA normalised expression of MYC based on Gene 1.0 ST array data. No significant differences were found between groups of samples that showed copy number gain in the region and those that did not. (0.15 MB TIF) [file pone.0009983.s010.tif]
